# Coordinated Developmental Remodeling of IGF/FGF–MAPK Signaling and Cytoskeletal Plasticity Coincides with the Loss of Cardiac Regenerative Capacity

**DOI:** 10.3390/cells15100873

**Published:** 2026-05-11

**Authors:** Natalia Kubin, Praveen Gajawada, Thomas Körtl, Andre Schneider, Lu Han, Laura C. Zelarayán, Thomas Braun, Samuel Sossalla, Yeong-Hoon Choi, Manfred Richter

**Affiliations:** 1 Department of Cardiology, Kerckhoff Campus, Justus Liebig University, 61231 Bad Nauheim, Germany; n.kubin@kerckhoff-klinik.de (N.K.); s.sossalla@kerckhoff-klinik.de (S.S.); 2 Medical Clinic I, Department of Cardiology and Angiology, Justus Liebig University, 35392 Giessen, Germany; thomas.koertl@innere.med.uni-giessen.de (T.K.); laura.zelarayan@med.uni-goettingen.de (L.C.Z.); 3 Department of Cardiac Surgery, Kerckhoff Heart Center, 61231 Bad Nauheim, Germany; p.gajawada@kerckhoff-klinik.de (P.G.); y.choi@kerckhoff-klinik.de (Y.-H.C.); 4 Max-Planck-Institute for Heart and Lung Research, 61231 Bad Nauheim, Germany; andre.schneider@mpi-bn.mpg.de (A.S.); lu.han@mpi-bn.mpg.de (L.H.); thomas.braun@mpi-bn.mpg.de (T.B.); 5 Institute of Pharmacology & Toxicology, University Medical Center Göttingen, 37075 Göttingen, Germany; 6 German Centre for Cardiovascular Research (DZHK), partner sites Lower Saxony, 37099 Göttingen, Germany

**Keywords:** cardiac regeneration, regenerative competence, MAPK signaling, B-Raf, IGF/FGF signaling, growth factor receptors, postnatal maturation, cardiomyocytes, cytoskeletal remodeling, receptor plasticity

## Abstract

Postnatal loss of cardiac regenerative capacity coincides with profound remodeling of signaling, structural, and metabolic programs in the developing heart. Here, we profiled Insulin growth factor (IGF)/Fibrobrast growth factor (FGF)/insulin receptors (InsR), Ras/Raf/MEK/ERK pathway components, cytoskeletal markers, and cell-cycle/metabolic proteins in mouse whole-heart tissue at P3, P7, P14, P28, and adulthood. IGF-1R- and IGF-2R-associated signals declined sharply during maturation, whereas InsR changed more modestly. FGFR1-derived immunoreactive species showed a transient early postnatal increase before marked reduction at later stages. These receptor-associated changes paralleled strong decreases in B-Raf, MEK1, and MEK2, together with pronounced loss of MEK1/2 activation-loop phosphorylation. MEK1 Thr292 phosphorylation also declined markedly, identifying a previously unrecognized developmental phosphorylation pattern. Structural maturation was accompanied by stable Actn2 expression, downregulation of immature cytoskeletal markers, increased cytochrome c and myoglobin, and significant loss of Aurora B and phospho-histone H3 in adult hearts. Together, these findings describe a coordinated postnatal maturation program in which signaling, cytoskeletal remodeling, metabolism, and proliferative withdrawal change in parallel. These data are consistent with reduced MAPK pathway activity during maturation and highlighting this signaling as node associated with closure of the neonatal regenerative window.

## 1. Introduction

Postnatal cardiac maturation is a tightly regulated transition from a highly proliferative neonatal myocardium to a terminally differentiated adult heart with limited regenerative capacity. Neonatal mammals, including human infants, can recover structurally and functionally after severe cardiac injury far more effectively than adults [1,2,3,4,5,6,7,8,9,10,11]. In contrast, adult hearts usually respond with scar formation and incomplete functional recovery. This developmental loss of regenerative competence coincides with cardiomyocyte cell-cycle withdrawal, sarcomeric maturation, metabolic remodeling, and extracellular matrix reorganization.

Growth factor receptor signaling pathways are closely linked to these processes. Among them, the insulin/IGF axis and FGFR1-dependent pathways are key upstream components associated with the Ras/Raf/MEK/ERK cascade, a central component of the MAPK signaling pathway. This pathway is associated with cardiomyocyte growth, myofibrillogenesis, and remodeling in a developmental stage-dependent manner [12,13,14,15]. Genetic studies have identified distinct age-dependent roles of insulin, IGF, and FGF receptor signaling in cardiac growth, metabolism, and survival [16,17,18,19,20]. We hypothesized that postnatal loss of cardiac regenerative competence is associated with coordinated downregulation of growth factor receptor/MAPK signaling occurring in parallel with structural, metabolic, and cell-cycle maturation.

The aim of this study was to characterize postnatal developmental changes in growth factor receptors and the Ras/Raf/MEK/ERK signaling axis. We analyzed receptor abundance, Raf isoform dynamics, MEK1/2 expression, and site-specific phosphorylation, and related these changes to markers of structural maturation, metabolic remodeling, and cell-cycle exit.

Previous studies have shown that extracellular factors derived from diseased human myocardium can reactivate MAPK-associated signaling in cultured adult cardiomyocytes and are associated with partial dedifferentiation [2,11,21,22,23].

## 2. Materials and Methods

### 2.1. Tissue Samples

All animal procedures were conducted in accordance with institutional and national guidelines for animal welfare and complied with §4 of the German Animal Welfare Act (TierSchG). As no experimental interventions were performed prior to organ collection, formal ethical approval was not required under local regulations. All procedures were carried out under institutional oversight at the Max Planck Institute for Heart and Lung Research (Bad Nauheim, Germany).

Wild-type *Mus musculus* (C57BL/6J) were bred and maintained in the institute’s in-house animal facility under standardized, species-appropriate conditions. No more than two animals per litter were included per experimental group to limit potential litter effects. Litter was considered a biological variable; however, the study was not powered to assess litter-specific effects. Inclusion criteria were intact cardiac tissue, successful homogenization, and sufficient protein yield. Exclusion criteria included visible tissue damage, insufficient protein concentration, or uneven/incomplete protein transfer during Western blot quality control. Neonatal (postnatal day 3, P3) and adult (8-week-old, P56) mice of both sexes were included, and additional developmental stages (P7, P14, and P28) were analyzed for temporal profiling. Animals were euthanized by cervical dislocation performed by trained personnel in accordance with §4 TierSchG.

Following euthanasia, hearts were rapidly excised, rinsed in ice-cold phosphate-buffered saline (PBS) to remove residual blood, snap-frozen in liquid nitrogen, and stored at −80 °C until further processing. For all analyses, whole hearts were used without dissection into anatomical subregions; thus, all measurements represent composite signals from mixed cardiac cell populations, including cardiomyocytes and non-myocyte cell types.

Tissue processing until homogenization was performed under cold conditions to preserve protein integrity and phosphorylation status. Then, frozen samples were homogenized in power buffer (composition described in the Western blot section) supplemented with protease and phosphatase inhibitors, using a volume adjusted to yield approximately 20 µg protein per µL lysate. Homogenization was performed by probe sonication (five short cycles) to ensure efficient cell lysis while minimizing heat-induced protein degradation. Lysates were centrifuged at ≥12,000× *g* for 5 min to remove insoluble debris, and the supernatant was collected for downstream analyses. Protein concentration was determined using the DC™ Protein Assay Kit II (Bio-Rad, Hercules, CA, USA) according to the manufacturer’s instructions.

Animals were analyzed without sex stratification; sex was not recorded separately, and both sexes were included without subgroup analysis. As the study was designed to assess developmental stage-dependent remodeling rather than sex-specific effects, it was not powered to detect sex differences. Also, blinding was not formally implemented in this study; however, all sample processing and analyses were performed using standardized protocols, which is acknowledged as a limitation.

### 2.2. Western Blots and Immunofluorescence

Immunoreactive proteins were analyzed by immunofluorescence microscopy (THUNDER Imager, Leica Microsystems, Wetzlar, Germany) and by Western blotting using the Bio-Rad ChemiDoc imaging system. Band intensities were quantified using the Bio-Rad Image Lab workflow with identical background subtraction parameters applied consistently within each experiment. Exposure times were selected within the non-saturated linear detection range of the imaging system. Representative uncropped full-length blots and corresponding membrane maps are provided in the Supplementary Figures and are referenced alongside the respective main figures.

To account for the higher muscularity and fibrosis of mature compared with immature hearts, tissue homogenization was performed using a high-strength lysis (“power”) buffer consisting of 0.1 M Tris-HCl, 10% sodium dodecyl sulfate (SDS), 10 mM ethylenediaminetetraacetic acid (EDTA), and 0.15 M dithiothreitol (DTT), pH 8.0. This extraction buffer was selected because it provided efficient and reproducible solubilization of cardiac tissue across all developmental stages, yielding minimal insoluble residue after centrifugation. The same extraction protocol was applied uniformly to all age groups to maximize comparability.

The buffer was supplemented with protease and phosphatase inhibitors (0.3 µM aprotinin, 4.2 µM leupeptin, 2 mM phenylmethylsulfonyl fluoride, 1 mM sodium vanadate, and 20 mM sodium fluoride). Tissue homogenates were sonicated, boiled for 1 min at 100 °C, and centrifuged at 14,000× *g* for 2 min to remove insoluble material.

The lysate supernatant was mixed with 5× Laemmli sample buffer (1 M Tris-HCl, pH 6.8, 10% SDS, 50% glycerol) in a 6:4 ratio, and 15 µg total protein per lane was separated on NuPAGE^®^ 4–12% Bis-Tris gradient gels (Invitrogen, Carlsbad, CA, USA),together with 3 µL molecular weight marker (MultiMark™ Multi-Colored Standard, Invitrogen). Proteins were transferred to nitrocellulose membranes (Amersham, Cytiva, Marlborough, MA, USA).

Total protein staining (RedAlert) was used to assess loading consistency and transfer quality across samples. Membranes with uneven transfer or insufficient quality were excluded from analysis. Densitometric analyses were performed under equal protein loading conditions and are presented as relative values. Housekeeping proteins such as GAPDH were not used as quantitative normalization factors, given potential developmental variability. RedAlert staining served as quality control for loading consistency but was not used as a quantitative densitometric normalization factor. Densitometric analysis of immunoblots was performed using Bio-Rad Image Lab software 6.1. Band intensities were quantified under standardized loading conditions. Data are presented as relative values and reflect semi-quantitative measurements of protein abundance. Given the inherent limitations of immunoblot-based quantification, these analyses are intended to capture relative differences and developmental trends rather than provide precise absolute quantification. Variability across biological replicates should therefore be interpreted with caution.

Immunoreactive proteins on nitrocellulose membranes were visualized using horseradish peroxidase (HRP)-conjugated secondary antibodies and enhanced chemiluminescence detection (SuperSignal™ West Femto, Thermo Scientific, Thermo Fisher Scientific, Waltham, MA, USA). Representative uncropped full-length blots and corresponding membrane maps are provided in the Supplementary Materials.

For phosphoprotein analysis, phospho-signals and corresponding total protein signals were analyzed in parallel; however, exact phospho/total ratio determination was not feasible for certain targets such as MEK1/2 because overlapping electrophoretic migration of closely related isoforms prevented reliable isoform-resolved quantification.

Antibody specificity was supported by manufacturer validation data, expected molecular weight assignment, and prior siRNA-mediated knockdown experiments in cardiomyocyte cultures for selected targets including FGFR1, OSMR, A-Raf, MEK1/2, and ERK1/2. Nevertheless, band identities in whole-heart tissue remain inferential unless directly validated by orthogonal approaches. Accordingly, where direct confirmation was not available, bands were interpreted as immunoreactive species consistent with the expected targets.

The following primary antibodies were used for target detection (catalog numbers in parentheses):

From Cell Signaling Technology, Danvers, MA, USA: IGF-1Rβ [9750], IGF-2R [14364], InsR [23413], FGFR1 [9740], PDGFRβ [3169], H/K/N-Ras [67648], A-Raf [75804], B-Raf [14814], C-Raf [53745], MEK1 [9146], MEK2 [9147], phospho-MEK1 (Thr292) [26975], phospho-MEK1/2 (Ser217/221-Ser221/225) [3958], PKM1/2 [3190], ERK1/2 [4695], phospho-ERK1/2 (Thr202/Tyr204) [4370], troponin I (TnI) [13083], cofilin-1 [5175], profilin 1 [3237], moesin [3150], ezrin [3145], Ezrin/Radixin/Moesin (ERM) [3142], phospho-ERM (Thr567/Thr564/Thr558) [3149], merlin [12888], aurora B [3094], phospho-histone H3 (P-H3) [9701], cytochrome c [4280], myoglobin [25919], Gapdh [5174]. From R&D Systems (R&D Systems, Minneapolis, MN, USA): Mouse oncostatin M receptor (mOSMR) [AF662]. From Invitrogen (Thermo Fisher Scientific, Waltham, MA, USA): Cardiac troponin C (cTnC) [MA1-22698]. From Abcam (Cambridge, United Kingdom): troponin T (TnT) [ab8295], sarcomeric α-actinin-2 (Actn2) [ab137346], non-muscle α-actinin-1 (Actn1) [ab68194], smooth muscle α-actin (Acta2) [ab32575], moesin [ab52490], radixin [ab52495]. From Sigma-Aldrich (Merck KGaA, Darmstadt, Germany): Sarcomeric α-actin (detects both Acta1 and Actc1) [SA-A2172], destrin [SA-D8815]. Gift from Dr. Eppenberger: myomesin (Myom).

### 2.3. Statistical Analysis

Mouse hearts from postnatal day 3 (P3) animals were used as the reference group (set to 100%). Statistical analyses were performed using GraphPad Prism 8 (GraphPad Software, Boston, MA, USA). Data are presented using Student’s *t*-test with mean ± standard error of the mean (SEM) in all figures.

Comparisons across multiple developmental time points were performed using one-way analysis of variance (ANOVA) followed by Tukey’s multiple comparisons test and are provided in the Supplementary Materials. Statistical approaches were applied consistently across all main and supplementary datasets. Statistical significance relative to the P3 reference group is indicated in the figures as follows: *p* < 0.05 (*), *p* < 0.01 (**), and *p* < 0.001 (***), with red asterisks indicated statistically significant.

## 3. Results

### 3.1. Targets and Strategies in the Study Design

To characterize postnatal changes in MEK1/2 expression and phosphorylation within the Ras/Raf/MEK/ERK signaling cascade, we analyzed selected MAPK pathway components together with upstream receptor tyrosine kinases. In parallel, we examined proteins associated with myocardial phenotypes, including markers of contractile organization, differentiation, cytoskeletal remodeling, cell-cycle activity, and metabolic maturation.

**Figure 1:**
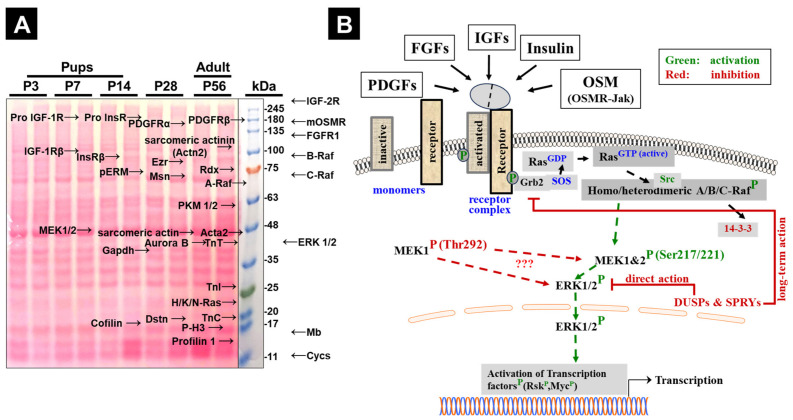
Blotted membrane showing protein separation from postnatally developing hearts and schematic overview of receptor-mediated Ras/Raf/MEK/ERK signaling. (**A**) Representative Western blot membrane of whole-heart lysates from P3, P7, P14, P28, and adult mice. Data are presented as mean ± SEM (*n* = 4 hearts per group; in panel A: *n* = 2). Statistical analysis was performed using Student’s *t*-test and is shown in the results. For completeness, we have included the corresponding ANOVA results in the Supplementary Materials. RedAlert total protein staining is shown to document transfer quality and comparable protein loading across ages. A colored molecular weight marker (kDa) is shown at the right, and approximate protein localizations are indicated. (**B**) Schematic overview of growth factor receptor regulation of the canonical Ras → Raf → MEK → ERK cascade in cardiac cells. Ligand binding to membrane receptors promotes receptor phosphorylation and recruitment of Grb2/SOS, leading to conversion of Ras^GDP^ to Ras^GTP^ and subsequent activation of Raf isoforms, which phosphorylate MEK and propagate MAPK signaling. Proteins and phosphorylation events reported to promote pathway activation are shown in green, whereas inhibitory regulators are shown in red.

Whole-heart lysates were prepared using a high-strength homogenization buffer (“power buffer”) to ensure efficient extraction of sarcomeric, nuclear, and membrane-associated proteins across postnatal stages (Figure 1A). We found empirically that weaker extraction buffers yielded less consistent recovery from more mature hearts, likely reflecting increasing muscularity and tissue complexity during development. The same extraction protocol was therefore used for all age groups. Proteins were separated by SDS-PAGE and transferred to nitrocellulose membranes. Molecular weights (kDa) were estimated using the colored size marker shown on RedAlert-stained membranes and were compared with UniProt annotations, antibody datasheets, and prior antibody specificity testing including siRNA-based validation in cultured cardiomyocytes for selected targets [23,24] (Figure 1A). Where direct molecular confirmation was not available, detected bands were interpreted as immunoreactive species consistent with the expected target proteins.

For all Western blots, samples were loaded in chronological order by age. Postnatal day 3 (P3) served as the reference group (100%) for graphical comparison and analysis by Student’s *t*-test (for one-way ANOVA, see Supplements). Age groups included P3, P7, P14, P28, and 8-week-old young adults (“adult”). This nomenclature is used consistently throughout the text and figure legends. Each time point included *n* = 4 individual hearts. Antibodies are listed in the Section 2. For simplicity, activating phosphorylation sites of MEK1 (Ser217/221) and MEK2 (Ser221/225) are referred to collectively as MEK1/2 (S217/221).

GAPDH is displayed for reference but was not used for normalization due to potential developmental variability. Total protein staining was used to assess loading and transfer quality across samples. Age groups included postnatal day 3 (P3), early postnatal pups at day 7 (P7) and day 14 (P14), an intermediate stage at day 28 (P28), and sexually mature young adults at 8 weeks (adult). This nomenclature is used consistently throughout the text and corresponding figure legends. The number of mice at each time point is *n* = 4. Antibodies used and validated for specificity are listed in the Section 2. Note that we refer to the activating phosphorylation sites of MEK1 (S217/221) and MEK2 (S221/225) collectively as MEK1/2 (S217/221).

### 3.2. Postnatal Cardiac Maturation Is Associated with Marked Downregulation of IGF-1R and IGF-2R Among the Analyzed Receptors

**Figure 2:**
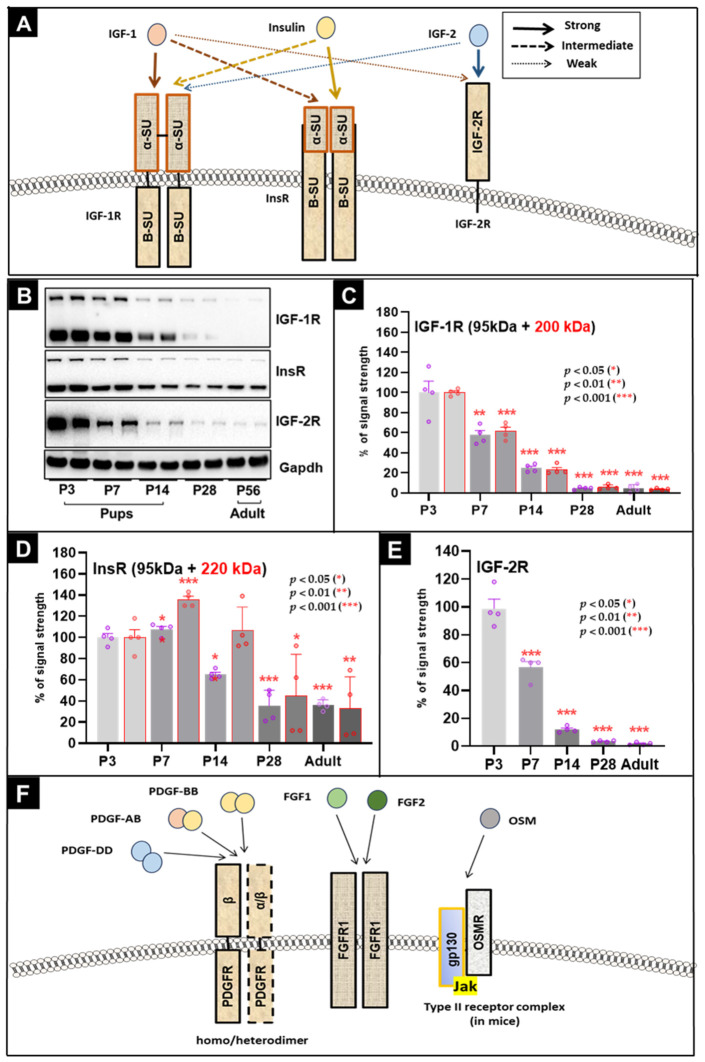

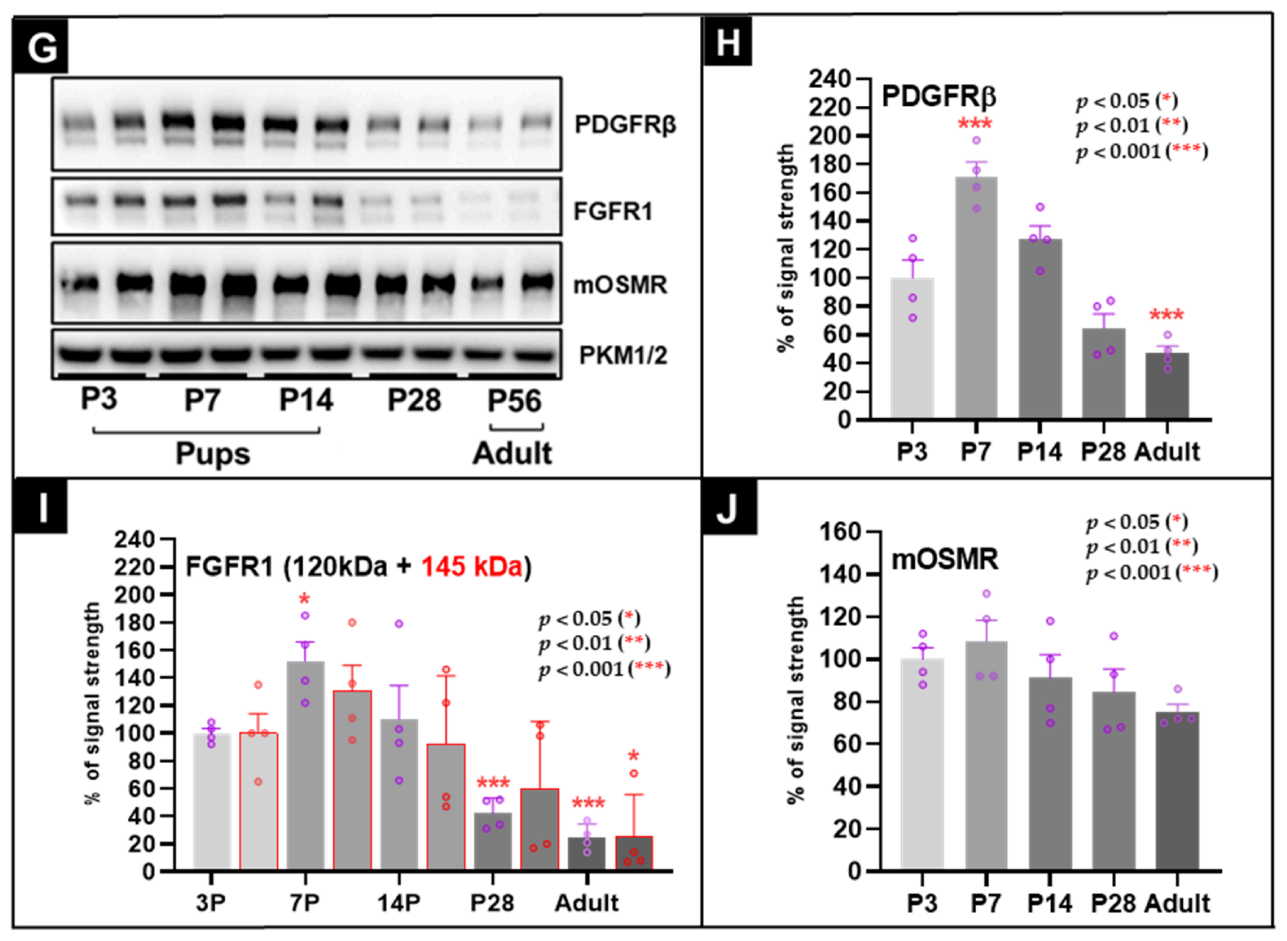
Expression dynamics of IGF-1R, IGF-2R, InsR, PDGFRβ, FGFR1, and OSMR in the mouse heart. (**A**) Scheme of IGF-1, IGF-2, and insulin receptor complexes, approximate molecular weights (kDa), and ligand–receptor interactions. Arrow thickness indicates reported relative ligand-binding strength. α-SU and β-SU indicate receptor subunits. Panel adapted and modified from [35]. (**B**) Representative Western blots of IGF-1R, IGF-2R, and InsR from mouse hearts. Blots were probed with antibodies against the β-subunits of IGF-1R and InsR; therefore, both higher-molecular-weight precursor-associated bands (IGF-1R ~200 kDa; InsR ~220 kDa) and lower-molecular-weight β-subunit-associated bands (~95 kDa) were detected. IGF-2R migrates at ~220 kDa. Molecular weights (kDa) are indicated. (**C**) Quantification of IGF-1R-immunoreactive bands. Both detected bands decline significantly by P7 and remain low thereafter. (**D**) Quantification of InsR-immunoreactive bands. The ~95 kDa band shows a small non-significant increase at P7, whereas the ~220 kDa band increases significantly at P7 and then declines during maturation. (**E**) Quantification of IGF-2R (CI-M6PR) immunoreactive signal. IGF-2R shows the strongest postnatal downregulation, falling to 57% by P7 and to 2% by P56. (**F**) Schematic representation of PDGF, FGF, and OSM receptor complexes and ligand–receptor interactions. PDGFRs form αα, αβ, or ββ dimers. PDGFRβ is activated by PDGF-AB, PDGF-BB, and PDGF-DD as indicated. Selected FGF ligands (FGF1 and FGF2) and FGFR1 are shown. In mice, oncostatin M signals via the type II OSMR/gp130 complex; in humans, OSM can also engage LIFR/gp130 (type I complex;). Panel adapted and modified from [35,36]. (**G**) Representative Western blots for PDGFRβ, FGFR1, and OSMR in mouse heart lysates. (**H**) Quantification of PDGFRβ band intensity. PDGFRβ is transiently increased at P7 and then declines during maturation. (**I**) Quantification of FGFR1-immunoreactive bands. Two FGFR1-associated bands (~145 kDa and ~120 kDa) were detected. Both show an early increase followed by marked decline in adulthood. (**J**) Quantification of mouse OSMR (mOSMR). OSMR expression does not change significantly during normal postnatal maturation. *p* < 0.05 (*), *p* < 0.01 (**), and *p* < 0.001 (***).

Figure 2 summarizes developmental changes in IGF-1R, IGF-2R, and InsR protein expression in the mouse heart. Figure 2A provides a schematic overview based on references [25,26,27], representative immunoblots are shown in Figure 2B, and quantitative analyses are shown in Figure 2C–E.

Using an antibody directed against the IGF-1R β-subunit, two immunoreactive bands were detected: an upper band at approximately 200 kDa and a lower band at approximately 95 kDa. These bands are consistent with the unprocessed IGF-1R precursor (200kDa) and the processed β-subunit (95kDa), respectively. Because this antibody recognizes the β-subunit sequence, both species can be detected. Quantification (Figure 2B,C) showed a pronounced decline in both IGF-1R-immunoreactive bands by P7. Band intensities at P7 were 57.5 ± 4.5% (*p* < 0.05) and 61.5 ± 4.0% (*p* < 0.001) of P3 levels, respectively. In adulthood, the expression declined further to 4.5 ± 1.9% (*p* < 0.01) and 3.75 ± 0.5% (*p* < 0.001) compared to neonatal levels. These data indicate rapid postnatal downregulation of IGF-1R-associated immunoreactive species, with very low signal in the adult heart.

For InsR, an antibody against the β-subunit detected two bands at approximately 220 kDa and 95 kDa, consistent with an unprocessed precursor and a processed β-subunit, respectively (Figure 2B,D). Quantification showed a modest, non-significant increase in the 95 kDa band to 107% at P7 and a significant rise in the 220 kDa band to 135.5 ± 8.0% (*p* < 0.01). Both bands then declined during postnatal maturation, reaching approximately 36.0 ± 4.5% (*p* < 0.001) and 33.0 ± 16.6% (*p* < 0.01) in adult hearts. Thus, InsR showed a transient early increase followed by sustained reduction in both detected receptor-associated species.

Figure 2B,E show the developmental profile of IGF-2R (also termed CI-M6PR). IGF-2R mainly functions as a clearance receptor and does not activate canonical IGF-1R-like signaling. It binds IGF2 and mannose-6-phosphate-tagged ligands and promotes their internalization and lysosomal targeting [28,29,30]. In our analysis, IGF-2R displayed the strongest postnatal decline among the receptors examined. Protein levels decreased to 56.5 ± 4.3% by P7 (*p* < 0.01) and to 1.75 ± 0.5% in adults (*p* < 0.001). These findings are consistent with strong developmental downregulation of IGF-2R-associated signal during postnatal heart maturation.

### 3.3. Biphasic Regulation of PDGFRβ and FGFR1, with Relatively Stable OSMR Expression, During Postnatal Cardiac Development

PDGFRα and PDGFRβ are receptor tyrosine kinases linked to MAPK signaling and are well known regulators of angiogenesis, arteriogenesis, and vascular stabilization during development [31,32,33,34]. Ligand binding promotes the formation of αα, αβ, or ββ receptor dimers (Figure 2F). The PDGFRβ signal increased transiently during the first postnatal week, reaching 171.5 ± 16.31% at P7 (*p* < 0.001). It then declined progressively to 47.0 ± 13.75% in adult hearts (*p* < 0.001). Given the use of whole-heart lysates, this pattern likely reflects changes in vascular-associated and other non-myocyte cell populations as well as overall cardiac maturation. 

The FGF/FGFR system also regulates vessel formation and cardiomyocyte growth, but interpretation is complicated by alternative splicing, post-translational modification, and promiscuous receptor dimerization [34,37]. Because FGFR1 and its ligands FGF1/FGF2 have documented roles in vascular and myocardial growth [34,38], we analyzed FGFR1 by Western blot (Figure 2G,I). Two FGFR1-associated bands were detected at approximately 145 kDa and 120 kDa. Both bands were abolished in prior siRNA knockdown experiments in cardiomyocyte cultures, supporting their assignment to FGFR1-derived immunoreactive species. However, direct distinction between specific isoforms and other molecular forms cannot be made from the present tissue blots alone. The 145 kDa band was approximately three times as intense as the 120 kDa band. Both bands showed an early upward trend at P7, which reached significance for the 120 kDa band (152.0 ± 14.36%, *p* < 0.05) but not for the 145 kDa band (130.5 ± 23.4%). Thereafter, both declined markedly. In adulthood, signal intensities were reduced to 24.75 ± 5.99% (*p* < 0.01) and 25.0 ± 21.02% (*p* < 0.05), respectively. These data are consistent with transient early enrichment of FGFR1-associated species followed by marked postnatal downregulation.

In contrast, OSMR showed no significant developmental regulation (Figure 2G,J). In mice, the OSMR/gp130 complex can activate multiple pathways, including MAPK, PI3K/Akt, p38, and Hippo signaling, and is often linked to pathological remodeling rather than normal postnatal maturation [21,24,39,40,41]. The absence of major developmental change in our dataset is therefore consistent with a more limited role for OSMR under physiological postnatal conditions.

Taken together, these results show receptor-specific developmental regulation. PDGFRβ- and FGFR1-associated signals display transient early increases followed by marked adult reduction, whereas OSMR remains comparatively stable.

### 3.4. H/K/N-Ras Changes Modestly, Whereas B-Raf Is Strongly Downregulated During Postnatal Heart Maturation

**Figure 3:**
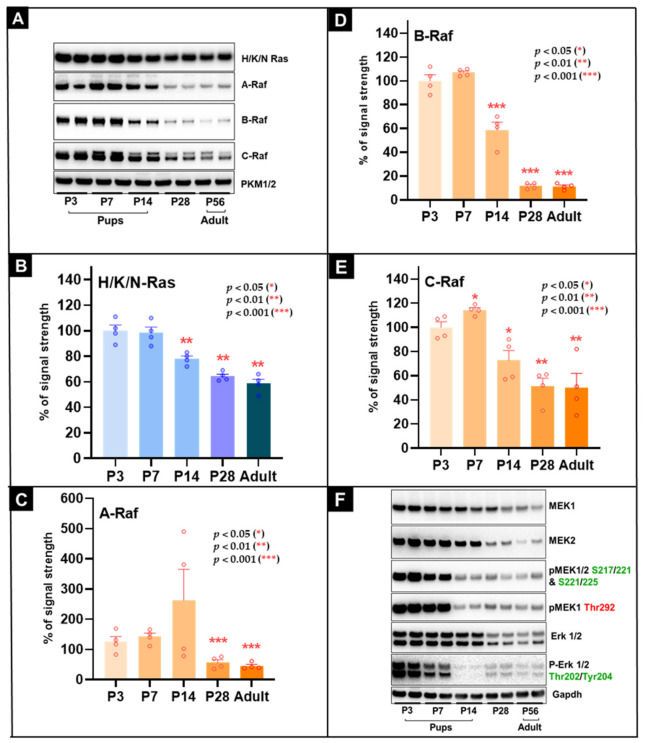

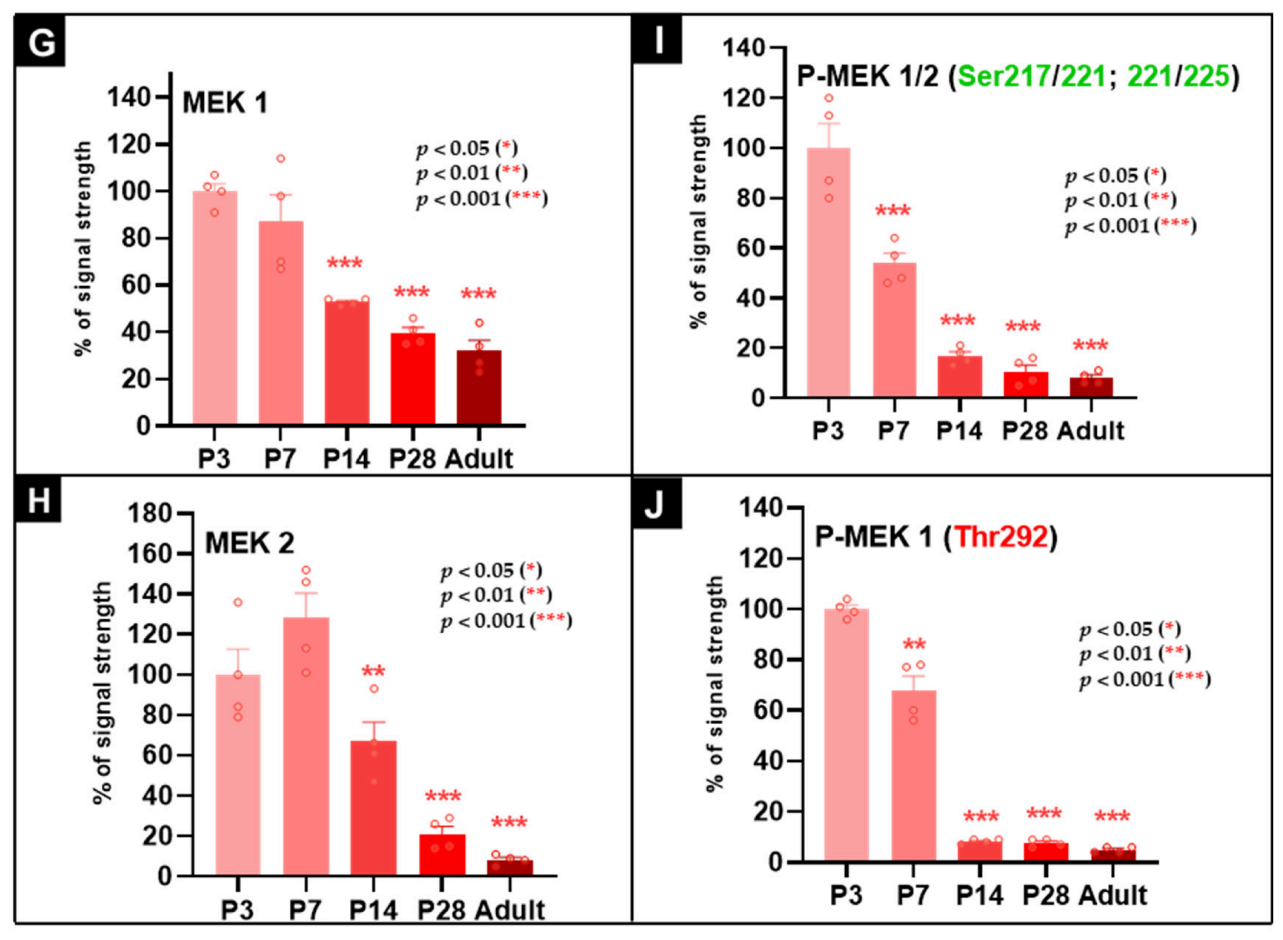
Postnatal reduction in B-Raf expression is accompanied by decreased MEK1/2 abundance and reduced regulatory phosphorylation in the mouse heart. (**A**) Representative Western blots for pan-Ras (recognizing H-, K-, and N-Ras), A-Raf, B-Raf, and C-Raf in mouse hearts at the indicated postnatal ages; quantification is shown in (**B**–**E**). (**B**) Ras signal declines significantly during maturation. (**C**) A-Raf shows a variable, non-significant increase at P14 followed by reduction in adults. (**D**) B-Raf is markedly downregulated during postnatal maturation, reaching ~12% of early postnatal levels by P28 and P56. (**E**) C-Raf shows a modest increase at P7 followed by reduction at later stages. (**F**) Representative Western blots for total MEK1, total MEK2, phospho-MEK1/2 (Ser217/221 for MEK1 and Ser221/225 for MEK2), phospho-MEK1 (Thr292), ERK1/2, and phospho-ERK1/2. (**G**) Total MEK1 declines significantly during maturation and reaches 32% in adult hearts. (**H**) Total MEK2 is significantly reduced by P28 and declines further to 8.3% in adults. (**I**) Phospho-MEK1/2 decreases markedly during maturation, reaching 8% in adults. (**J**) Phospho-MEK1 (Thr292) also declines sharply, reaching 5% in adults. Phosphorylation changes are presented as relative phosphorylation patterns and are consistent with reduced MAPK pathway activity during postnatal maturation. *p* < 0.05 (*), *p* < 0.01 (**), and *p* < 0.001 (***).

To assess developmental changes in the Ras/Raf/MEK/ERK module after birth, we measured H/K/N-Ras and the three Raf isoforms by Western blot in mouse hearts (Figure 3A; quantification in Figure 3B–E). The H/K/N-Ras signal was highest in the early postnatal period and declined gradually with maturation. By P14, Ras levels were 78.0 ± 5.1% (*p* < 0.01) and fell further to 58.0 ± 5.7% in adult hearts (*p* < 0.01) (Figure 3B). The antibody used does not distinguish between H-Ras, K-Ras, and N-Ras. The three Raf isoforms displayed distinct developmental profiles. A-Raf increased to 262.8 ± 103.8% at P14, but this change was not statistically significant because of substantial inter-sample variability. The signal then declined to 45.0 ± 18.6% in adults (*p* < 0.001) (Figure 3C). B-Raf showed the strongest and earliest reduction. By P14, B-Raf had fallen to 58.8 ± 8.3% (*p* < 0.001), and by P28 and adulthood the signal was reduced to approximately 12% of baseline (11.8 ± 5.3% and 11.3 ± 5.3%; both *p* < 0.001) (Figure 3D). C-Raf showed a modest early increase at P7 (114.3 ± 5.0%; *p* = 0.003), followed by decline to 51.0 ± 8.3% at P28 (*p* < 0.01) and 51.0 ± 12.6% in adults (*p* < 0.01) (Figure 3E).

Overall, these data indicate isoform-specific developmental regulation within the Ras/Raf module. Among the Raf kinases, B-Raf displayed the most pronounced postnatal reduction, whereas A-Raf was more variable and C-Raf declined more moderately.

### 3.5. Postnatal Loss of B-Raf and IGF-1R/IGF-2R Parallels Strong Reduction in MEK1/2 Phosphorylation, Including the Regulatory Thr292 Site of MEK1

MEK1 and MEK2 are central components of the Ras/Raf/MEK/ERK cascade (Figure 1B). To assess how this module changes after birth, we analyzed total MEK1 and MEK2 protein together with two classes of regulatory phosphorylation: activating phosphorylation at Ser217/221 (MEK1) and Ser221/225 (MEK2), detected collectively by the phospho-MEK1/2 antibody, and phosphorylation at the regulatory MEK1 Thr292 site. Representative blots are shown in Figure 3F and quantification in Figure 3G–J.

Total MEK1 protein remained relatively stable during the first postnatal week, then decreased to 52.8 ± 0.8% by P14 (*p* < 0.001) and to 32.0 ± 5.6% in adult hearts (*p* < 0.001) (Figure 3G). MEK2 followed a similar but delayed pattern, declining significantly by P28 to 21.0 ± 3.5% (*p* < 0.001) and to 8.3 ± 1.3% in adults (*p* < 0.001) (Figure 3H). Phosphorylation detected by the phospho-MEK1/2 activation-loop antibody declined even more strongly than total MEK protein, reaching 8.0 ± 1.3% in adults (*p* < 0.001; Figure 3I). Phosphorylation at MEK1 Thr292 also decreased markedly, reaching 5.0 ± 0.6% in adults (*p* < 0.001; Figure 3J). Thus, the reduction in phosphorylation signal exceeded the reduction in total MEK protein. For example, total MEK1 remained at 32% of P3 levels in adults, whereas phospho-MEK1/2 and phospho-Thr292 were reduced to 8% and 5%, respectively. The relatively high phosphorylation signal at P3 and the pronounced decline in phospho-Thr292 during maturation were notable observations. Because closely migrating MEK isoforms limit precise phospho/total assignment, these data are interpreted as relative phosphorylation patterns rather than direct measures of kinase activity. ERK1/2 and phospho-ERK1/2 also declined postnatally (Figure 3F), with ERK1 and ERK2 migrating at approximately 44 kDa and 42 kDa, respectively.

In summary, both MEK abundance and MEK-associated phosphorylation signals were strongly reduced during postnatal maturation. These changes are consistent with reduced MAPK pathway activity during transition from the neonatal to the mature heart.

### 3.6. Sarcomere Maturation from Neonatal to Adult Myocardium: Sharper Striation, Stable Actn2, Increased Sarcomeric Actin Signal, and Developmental Regulation of Troponin-Associated Proteins

**Figure 4:**
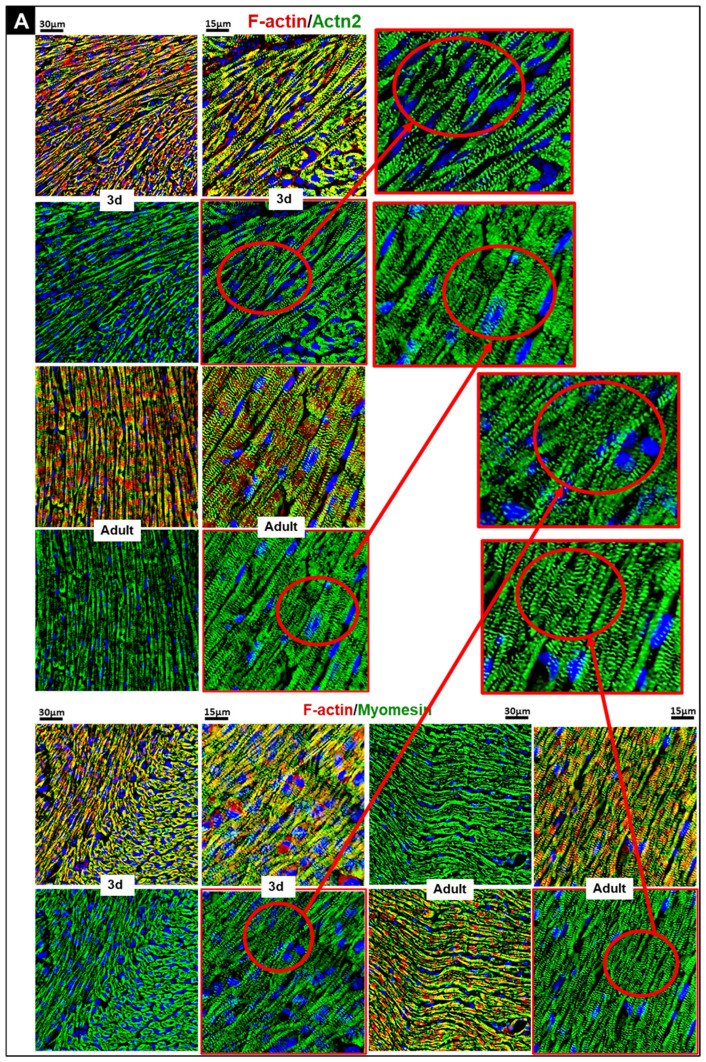

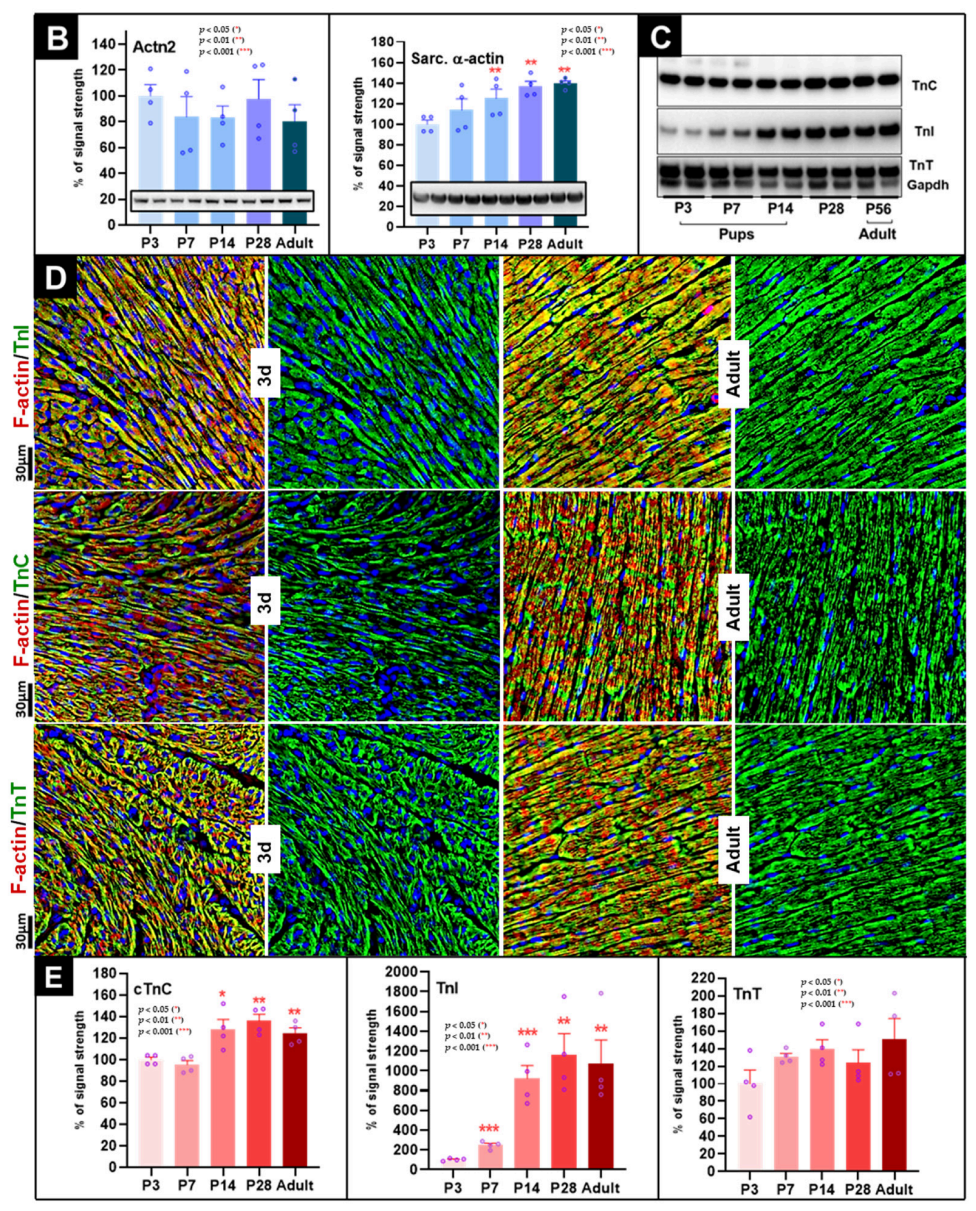
Enhanced sarcomeric striation with stable Actn2, increased sarcomeric α-actin signal, and developmental expression of troponin-associated proteins in the myocardium. (**A**) Representative immunofluorescence images of left-ventricular myocardium from P3 and adult mice. Samples were co-stained for Actn2 and myomesin (green) and filamentous actin (phalloidin; red); nuclei were counterstained with DAPI (blue). Neonatal myocardium shows less sharply defined sarcomeric organization, whereas adult cardiomyocytes display more regular cross-striation consistent with mature sarcomere architecture. (**B**) Representative Western blots and quantification of Actn2 and sarcomeric α-actin across postnatal ages. Actn2 shows no significant change during maturation. Sarcomeric α-actin-associated signal increases progressively, reaching 140% in adulthood. (**C**) Western blots of TnC, TnI, and TnT. P3 samples serve as the reference baseline (100%). (**D**) Representative fluorescence images of P3 and adult mouse heart tissue stained for TnC, TnI, and TnT (green). F-actin is shown in red and nuclei in blue (DAPI). The scale bar represents 30 µm. Neonatal tissue shows less regular sarcomeric organization than adult myocardium. (**E**) Quantification (*n* = 4 hearts). TnC increases significantly by day 14 and remains moderately elevated. TnI-associated signal rises strongly during maturation, whereas TnT shows only modest postnatal variation. Because the TnI antibody is not isoform-specific, this increase should be interpreted with caution. *p* < 0.05 (*), *p* < 0.01 (**), and *p* < 0.001 (***).

To assess postnatal maturation of sarcomeric organization, we examined the localization of key structural proteins, Actn2 and myomesin, in P3 and adult mouse hearts by fluorescence microscopy (Figure 4A). In parallel, Actn2 and sarcomeric α-actin were analyzed by Western blot (Figure 4B). Actn2 is a Z-disk actin-crosslinking protein required for sarcomere assembly and structural stability [42], whereas myomesin localizes to the M-line and links thick filaments [43].

In neonatal hearts, Actn2 staining appeared more diffuse and less sharply organized, and myomesin showed lower periodicity along sarcomeres, consistent with immature myofibrillar organization (Figure 4A). In adult tissue, cardiomyocytes displayed more regular cross-striation and more sharply aligned Actn2- and myomesin-associated structures, consistent with mature sarcomere organization.

Western blot analysis showed that Actn2 protein abundance remained essentially stable across postnatal development and did not differ significantly from P3 levels. In contrast, the sarcomeric α-actin signal increased progressively, reaching 139.8 ± 4.0% in adult hearts (*p* < 0.001) (Figure 4B). The antibody used recognizes both skeletal and cardiac α-actin isoforms, therefore this increase may reflect altered isoform composition, altered epitope recognition, or increased total signal intensity rather than a simple increase in one specific sarcomeric α-actin species.

We next analyzed troponin C (TnC), troponin I (TnI), and troponin T (TnT) by immunoblotting and fluorescence microscopy (Figure 4C–E). TnC increased modestly during postnatal development, reaching 128.3 ± 9.3% at P14 (*p* < 0.05) and remaining elevated in adults (124.3 ± 5.7%, *p* < 0.01). TnT showed only limited change, with a modest non-significant increase at P7 (130.8 ± 4.5%) and no consistent further increase thereafter.

By contrast, the TnI antibody yielded a large progressive increase in signal intensity. TnI-associated signal was elevated by P7 (247.3 ± 19.6%, *p* < 0.001) and peaked at P28 (1166 ± 209%, *p* < 0.05). Because TnC and TnT changed only modestly, a more than 10-fold increase in total TnI protein would be biologically unexpected given troponin complex stoichiometry. The most plausible explanations are developmental isoform switching and/or assay-dependent antibody behavior, including differences in epitope accessibility [44]. The antibody used is not isoform-specific, and therefore the observed TnI signal cannot be interpreted as a direct quantitative measure of one defined TnI isoform. Accordingly, the large increase in TnI-associated signal is interpreted cautiously.

Together, these data show progressive maturation of sarcomeric organization during postnatal development. TnC and TnT change modestly, whereas the large rise in the TnI-associated signal is most likely influenced by isoform switching and antibody recognition characteristics rather than by a simple quantitative increase in total TnI abundance.

### 3.7. Coordinated Downregulation of Dedifferentiation-Associated Markers and Actin-Regulatory Proteins During Postnatal Cardiac Maturation

**Figure 5:**
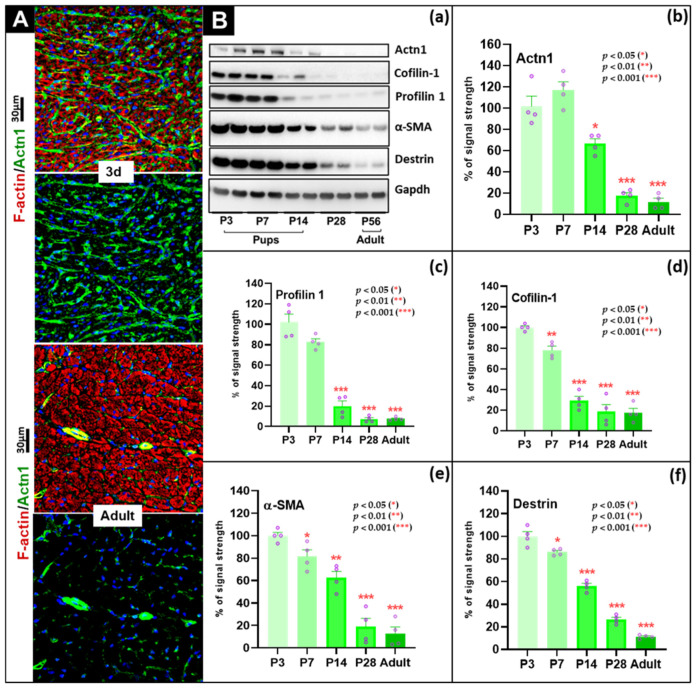
Postnatal downregulation of dedifferentiation-associated markers and actin-regulating proteins during cardiac maturation. (**A**) Fluorescence microscopy images of mouse myocardium stained for non-muscle α-actinin-1 (Actn1; green). F-actin is visualized in red and nuclei in blue (DAPI). The scale bar represents 30 µm. P3 myocardium shows broad Actn1 distribution, whereas in adult myocardium signal is strongly reduced in the myocardial compartment and largely retained in vascular structures. (**B**) Western blots (**a**) and quantification (**b**–**f**) of Actn1 and actin-regulatory proteins. (**b**) Actn1 shows a transient rise at P7 followed by marked decline in adulthood. (**c**) Profilin-1 decreases significantly from P14 onward. (**d**) Cofilin-1 declines significantly during postnatal maturation. (**e**) Acta2 declines significantly during maturation. (**f**) Destrin levels also decrease significantly, reaching low adult levels. *p* < 0.05 (*), *p* < 0.01 (**), and *p* < 0.001 (***).

To evaluate the transition from a more plastic neonatal phenotype to a mature differentiated state, we analyzed proteins previously associated with cytoskeletal remodeling and dedifferentiation, including destrin, non-muscle α-actinin-1 (Actn1), profilin-1, cofilin-1, and α-smooth muscle actin (Acta2), by immunofluorescence (Figure 5A) and Western blotting (Figure 5B). Fluorescence microscopy of whole-heart sections showed clear differences between P3 and adult myocardium. In P3 tissue, Actn1 immunoreactivity was broadly distributed, with enrichment at cell–cell contacts, perivascular regions, and within the cytoplasm, consistent with a more dynamic immature cytoskeletal organization. In adult hearts, Actn1 signal was markedly reduced in the myocardium and was largely restricted to vascular structures (Figure 5A). This pattern is consistent with the whole-heart nature of the samples and indicates that non-myocyte cells contribute to the detected signal. Immunoblotting showed coordinated postnatal decline of these proteins. Actn1 showed a transient non-significant increase at P7 (117 ± 12.5%), followed by marked reduction to 11.8 ± 10.3% in adults (*p* < 0.001) (Figure 5B(b)). Profilin-1 decreased to 20.0 ± 4.9% by P14 (*p* < 0.001) and to 7.5 ± 0.9% in adults (*p* < 0.001) (Figure 5B(c)). Cofilin-1 fell to 78.0 ± 4.2% at P7 (*p* < 0.01) and to 17.3 ± 4.6% in adults (*p* < 0.001) (Figure 5B(d)). Acta2 declined to 81.5 ± 6.6% by P7 (*p* < 0.05) and to 12.8 ± 6.5% in adult hearts (*p* < 0.001) (Figure 5B(e)). Destrin showed a similar pattern, decreasing to 86.0 ± 4.4% at P7 (*p* < 0.05) and to 11.3 ± 4.3% in adults (*p* < 0.001) (Figure 5B(f)).

These proteins have been linked previously to immature or remodeling-associated cytoskeletal states and are reported to reappear in pathological settings in rat, mouse, and human myocardium [2,11,14,22,24,45,46]. In the present dataset, their coordinated reduction coincided with increasing sarcomeric organization during maturation. Because the measurements were obtained from whole-heart homogenates, these changes should be interpreted as whole-heart developmental patterns rather than exclusively cardiomyocyte-specific events. In summary, the data show a robust postnatal decline in several cytoskeletal and actin-regulatory proteins associated with immature or remodeling-prone states, consistent with reduced structural plasticity in the mature heart.

### 3.8. Developmental Downregulation of ERM Family Members and Merlin Parallels Terminal Maturation of the Postnatal Heart

**Figure 6:**
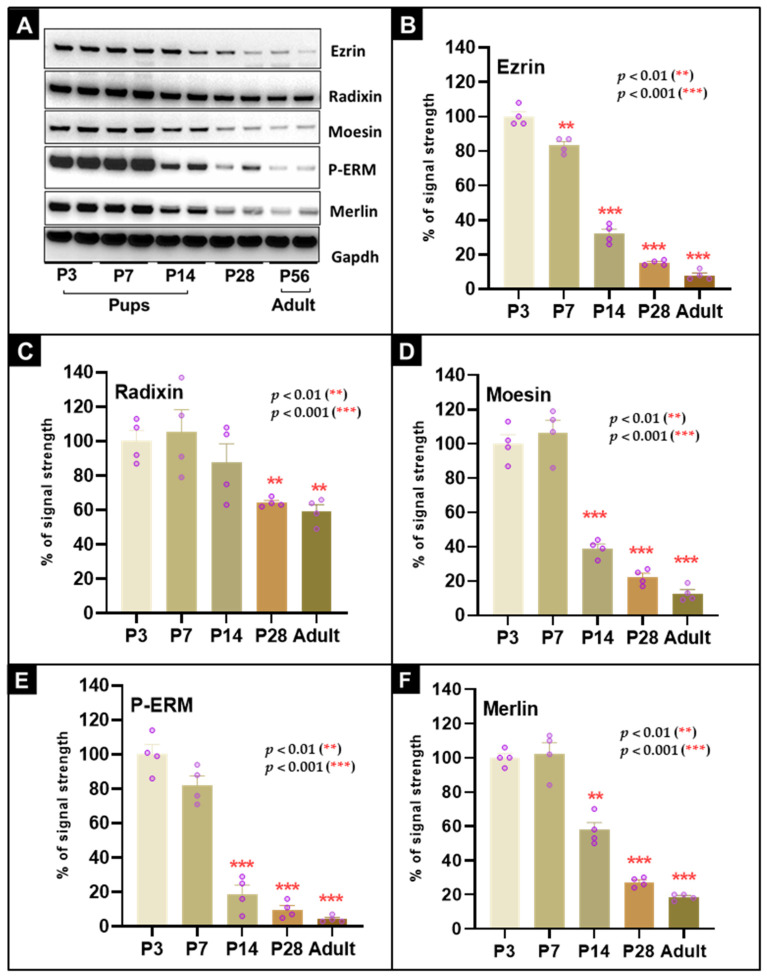
Expression and phosphorylation-associated activation state of cardiac ERM proteins decline. (**A**) Western blots showing expression of ezrin, radixin, moesin, phospho-ERM (Thr567 in ezrin, Thr564 in radixin, Thr558 in moesin), and merlin during mouse postnatal development. (**B**) Ezrin decreases significantly from P7 onward and reaches 8% in adulthood. (**C**) Radixin declines more moderately and remains detectable in adult hearts. (**D**) Moesin decreases significantly from P14 onward and reaches low adult levels. (**E**) Phospho-ERM signal declines strongly during maturation. (**F**) Merlin decreases significantly during postnatal development. *p* < 0.01 (**), and *p* < 0.001 (***).

To assess the developmental regulation of the membrane–cytoskeleton linker family, we measured the expression and phosphorylation of ezrin, radixin, moesin, and the ERM-related protein merlin by immunoblotting (Figure 6A) with quantification in Figure 6B–F.

Ezrin declined early during postnatal maturation. By P7, ezrin levels had fallen to 83.3 ± 3.6% (*p* < 0.01) and then decreased progressively to 8.0 ± 1.3% in adults (*p* < 0.001) (Figure 6B). Radixin showed a later and more moderate decline, reaching 64.3 ± 6.3% at P28 (*p* < 0.01) and 59.3 ± 7.3% in adults (*p* < 0.01) (Figure 6C). Moesin remained relatively stable during the first postnatal week and then fell markedly to 39.0 ± 5.9% at P14 (*p* < 0.001) and to 12.8 ± 5.8% in adults (*p* < 0.001) (Figure 6D).

Because ERM activation is commonly associated with C-terminal threonine phosphorylation, we also analyzed phospho-ERM using an antibody recognizing the conserved phosphorylated threonine residue in ezrin, radixin, and moesin. Phospho-ERM signal remained relatively stable through P7 but then declined steeply to 19.0 ± 7.8% at P14 (*p* < 0.001) and to 4.3 ± 2.2% in adults (*p* < 0.001) (Figure 6E). Merlin was progressively downregulated as well, decreasing to 57.8 ± 5.0% at P14 (*p* = 0.01) and to 18.5 ± 2.6% in adults (*p* < 0.001) (Figure 6F).

Collectively, these data show coordinated developmental downregulation of ERM family proteins, phospho-ERM, and merlin during postnatal maturation. Radixin was retained to a greater extent than ezrin or moesin.

### 3.9. Increases in Myoglobin and Cytochrome c Occur in Parallel with Marked Reduction in Cell-Cycle-Associated Proteins During Postnatal Heart Development

**Figure 7:**
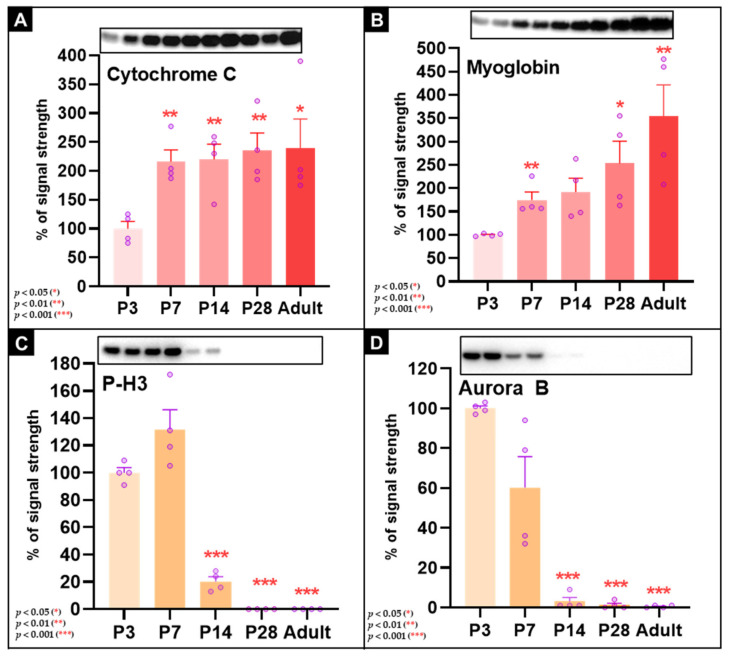
Increased cytochrome c and myoglobin expression and reduced Aurora B and phospho-histone H3 mark postnatal cardiac maturation. Western blot analysis and quantification of cytochrome c (**A**) and myoglobin (**B**) show increased expression during development. Cytochrome c rises significantly from P7 onward and remains elevated in adulthood. Myoglobin also increases progressively and reaches its highest levels in adults. Western blot and quantification of Aurora B (**C**) and phospho-histone H3 (**D**) show marked reduction in mitosis-associated signals during maturation. *p* < 0.05 (*), *p* < 0.01 (**), and *p* < 0.001 (***).

To assess metabolic and proliferative remodeling during postnatal maturation, we analyzed cytochrome c, myoglobin, Aurora B kinase, and phospho-histone H3 (P-H3; Ser10) by Western blot (Figure 7). Cytochrome c, a key mitochondrial electron transport protein, increased markedly by P7 to 216.0 ± 20.76% (*p* < 0.01) and remained elevated in adults at 239.3 ± 50.61% (*p* < 0.05) (Figure 7A). Myoglobin also increased progressively, reaching 174.8 ± 17.26% at P7 (*p* < 0.01) and 354.3 ± 67.37% in adults (*p* < 0.01) (Figure 7B). These changes are consistent with increased oxidative and oxygen-handling capacity during postnatal maturation.

In contrast, markers associated with mitotic activity declined sharply. The Aurora B signal decreased rapidly and was almost undetectable in adult hearts (*p* < 0.001) (Figure 7C). Phospho-histone H3 also became undetectable in adult myocardium (*p* < 0.001) (Figure 7D). These changes are consistent with marked reduction in proliferative activity during maturation.

Taken together, the data indicate a coordinated postnatal shift in which proteins associated with mitochondrial and oxygen-handling functions increase, whereas mitosis-associated proteins decrease to very low or undetectable levels.

## 4. Discussion

The postnatal transition of the mammalian myocardium is characterized by rapid and coordinated maturation. During this period, proliferative, metabolically flexible, and structurally plastic neonatal tissue is converted into a terminally differentiated organ optimized for mechanical efficiency but with markedly reduced regenerative competence. In the present study, we mapped developmental changes in receptor expression, MAPK-cascade components, cytoskeletal regulators, metabolic markers, and cell-cycle-associated proteins across early postnatal maturation. The data describe a coordinated developmental program in which signaling, structural organization, metabolism, and proliferative state change in parallel. Since the study was performed using whole-heart homogenates, findings should be interpreted as organ-level patterns rather than cardiomyocyte-specific mechanisms.

A central observation of this study is that multiple growth factor receptor- and MAPK-associated signals are strongly reduced during postnatal maturation. Several of these developmental changes also resemble patterns that we and others have previously described in adult cardiomyocytes exposed to cytokines, growth factors, or endothelial-derived morphogens, as well as in remodeling and failing hearts [2,11,22,23,24,38,45,47,48,49,50]. This similarity is consistent with the concept that pathways suppressed during normal maturation may become re-engaged during fetal-like remodeling in disease. However, the present data are descriptive and correlative and do not establish that these developmental changes directly govern regenerative competence or dedifferentiation.

### 4.1. Developmental Reduction in Upstream Receptor-Associated Signaling

Across the neonatal period, we observed marked developmental downregulation of several receptor systems linked to Ras/Raf/MEK/ERK signaling. The IGF axis showed the strongest decline. IGF-1R-associated signals were reduced to less than 5% of neonatal levels by adulthood, and IGF-2R showed an even more pronounced decline. These patterns are consistent with the established role of IGF receptor signaling in early cardiomyocyte growth, myocardial size regulation, and developmental remodeling [14,29,51,52,53,54,55]. In this context, the strong postnatal reduction in IGF-1R- and IGF-2R-associated signals is consistent with reduced growth factor responsiveness during maturation. By contrast, InsR-associated bands declined less markedly, suggesting that insulin-dependent metabolic functions may remain more preserved than IGF-linked developmental signaling during the postnatal transition [56].

FGFR1 showed a more complex developmental profile. Rather than declining monotonically, FGFR1-associated bands increased transiently during the first postnatal week and then decreased strongly at later stages. This pattern is consistent with a temporally restricted role of FGF-associated signaling during early postnatal growth and remodeling [24,38,45,47,57,58,59,60]. However, because the present study used whole-heart lysates and band identities cannot be resolved definitively as specific isoforms in this tissue context, these findings are best interpreted as developmental changes in FGFR1-derived immunoreactive species rather than direct proof of isoform-specific regulation.

PDGFRβ also displayed a biphasic pattern, with transient early elevation followed by decline in adulthood. Given the known enrichment of PDGFRβ in vascular-associated and other non-myocyte populations, this signal likely reflects developmental remodeling of non-cardiomyocyte compartments as well as whole-heart maturation. In contrast, OSMR remained relatively stable across development. This is consistent with its more established association with pathological and inflammatory signaling than with normal postnatal maturation [14,21,24,61].

Taken together, these data indicate a broad developmental reconfiguration of receptor-associated inputs to the Ras/MAPK network. Rather than reflecting loss of a single dominant receptor, the postnatal decline in pathway-associated signaling appears to involve multiple receptor systems with distinct temporal profiles. These coordinated changes are consistent with reduced trophic and mitogenic input during cardiac maturation.

### 4.2. Restructuring of the MAPK Cascade and the MEK1 Thr292 Phosphorylation Pattern

Downstream of these receptor changes, we observed coordinated developmental remodeling of the Ras/Raf/MEK/ERK module. Ras-associated signal declined only modestly, although isoform-specific regulation could not be resolved because the antibody detects H-, K-, and N-Ras together. In contrast, the Raf kinases showed more distinct isoform-specific profiles. Among them, B-Raf displayed the strongest reduction, falling to approximately 10–12% of early postnatal levels by P28 and adulthood. A-Raf and C-Raf also declined, but less uniformly. Because B-Raf is a potent activator of MEK1/2 and has been implicated in cardiomyocyte signaling and remodeling [14,23,24,62,63], its pronounced developmental reduction is consistent with major restructuring of MAPK-associated signaling during postnatal maturation. Total MEK1 and MEK2 protein also decreased during maturation. Importantly, phosphorylation detected at the MEK1/2 activation-loop site and at MEK1 Thr292 declined even more strongly than total MEK protein. These findings indicate that developmental changes in phosphorylation-associated signals exceed changes in total protein abundance. However, phosphorylation measurements in the present study should be interpreted with caution. Exact phospho/total ratio determination was not feasible for certain targets, particularly MEK1/2, because closely related isoforms show strongly overlapping migration and phospho-specific antibodies detect combined signals. Accordingly, these data are best interpreted as relative phosphorylation patterns that are consistent with altered MAPK pathway activity, rather than as direct quantitative measures of kinase output.

Collectively, these data indicate a multi-level reduction in MAPK pathway activity, including B-Raf depletion, reduced activation-loop phosphorylation, and loss of MEK1 Thr292 phosphorylation. This layered change in MAPK architecture aligns tightly with the window of cardiomyocyte cell-cycle withdrawal and the transition to structural maturation, consistent with MAPK pathway restructuring as a central feature associated with postnatal myocardial development. The developmental pattern of MEK1 Thr292 phosphorylation was particularly notable. The Thr292-associated signal was high in the early neonatal period and declined sharply during postnatal maturation. This observation is unexpected because Thr292 has often been discussed as a regulatory or inhibitory site in other contexts, and the close parallel between Thr292 and activation-loop phosphorylation was not anticipated. At present, the functional significance of this pattern in the heart remains unresolved. The data identify Thr292 phosphorylation as a developmentally regulated feature of the neonatal myocardium, but they do not establish whether it contributes to proliferation, cytokinesis, feedback regulation, or another aspect of postnatal remodeling. This finding therefore represents a descriptive observation that requires targeted mechanistic investigation.

Overall, the MAPK cascade undergoes pronounced developmental restructuring during postnatal maturation. This includes strong reduction in B-Raf, substantial loss of MEK-associated phosphorylation, and a coordinated decline in the Thr292 signal. Together, these changes suggest reduced cardiomyocyte plasticity during maturation.

### 4.3. Cytoskeletal Consolidation and Reduced Structural Plasticity

The signaling changes described above occur in parallel with marked cytoskeletal and sarcomeric remodeling. During postnatal maturation, sarcomeric organization becomes more regular and sharply aligned, whereas several proteins associated with immature or remodeling-prone cytoskeletal states decline strongly. Actn2 remained relatively stable, which is consistent with its structural role in the Z-disk [64]. In contrast, Actn1, Acta2, cofilin-1, profilin-1, and destrin were all reduced during maturation. Actn1 also showed a marked shift in localization, becoming largely restricted to vascular structures in adult tissue. These findings are consistent with the concept that whole-heart maturation is associated with the loss of a more dynamic cytoskeletal program and progressive stabilization of contractile architecture. Transforming growth factor-β (TGF-β), a central regulator of cardiac cytoskeletal remodeling and myofibroblast activation, is closely linked to Acta2 induction and actin reorganization, consistent with the concept that developmental suppression of plasticity-associated cytoskeletal proteins may parallel pathways reactivated during pathological remodeling [65,66].

ERM proteins and merlin showed a similar developmental trend. Ezrin, moesin, phospho-ERM, and merlin declined strongly, whereas radixin remained more readily detectable in adulthood. Because ERM proteins link cortical actin to the plasma membrane, reduction in total ERM and phospho-ERM signals is consistent with lower membrane–cytoskeleton remodeling capacity in the mature heart. At the same time, these observations should not be overinterpreted mechanistically. The present data show strong temporal association between ERM family downregulation, actin-regulatory protein loss, and structural maturation, but they do not directly test whether these proteins functionally control cytokinesis, membrane remodeling, or regenerative competence in postnatal cardiomyocytes.

Notably, cytoskeletal consolidation and metabolic reprogramming coincide temporally with the decline in regenerative competence. Increased sarcomeric organization and reduced cytoskeletal plasticity are likely to impose structural constraints on cytokinesis, while the shift toward oxidative metabolism may promote cell-cycle arrest through mitochondrial signaling pathways. Together, these changes are consistent with a progressive restriction of cardiomyocyte plasticity during postnatal maturation.

In this framework, our findings are consistent with a model in which progressive reduction in actin-remodeling proteins and membrane–cytoskeleton linkers accompany the establishment of a mechanically robust, low-plasticity adult myocardial phenotype.

### 4.4. Metabolic Maturation and Cell-Cycle Withdrawal

The structural changes observed here occur in parallel with metabolic maturation and near-complete loss of mitotic markers. Cytochrome c and myoglobin increased strongly after birth, consistent with increasing mitochondrial and oxygen-handling capacity during maturation [67]. In contrast, Aurora B and phospho-histone H3 declined to very low or undetectable levels by adulthood, indicating marked reduction in proliferative activity. These findings are in line with the well-established postnatal shift from a more glycolytic and proliferative neonatal state to an oxidative, hypertrophic, and terminally differentiated adult phenotype.

Cytoskeletal consolidation and metabolic reprogramming are both recognized contributors to postnatal cardiomyocyte cell-cycle exit. Increased sarcomeric organization imposes structural constraints that are incompatible with cytokinesis, whereas metabolic maturation toward oxidative phosphorylation is associated with changes in redox balance, mitochondrial activity, and signaling state that may reinforce cell-cycle arrest. In the present study, these structural and metabolic changes occur in parallel with reduced receptor-associated and MAPK-associated signaling. This does not establish causality, but it supports a model in which signaling and structural, metabolic, and proliferative maturation converge during closure of the neonatal regenerative window.

### 4.5. Technical and Interpretive Limitations

Several limitations should be considered when interpreting these findings. First, all analyses were performed on whole-heart homogenates. This approach is suitable for developmental profiling at the organ level, but it does not resolve the cellular origin of the measured signals. Consequently, proteins such as PDGFRβ, OSMR, Acta2, Actn1, and ERM family members may reflect contributions from fibroblasts, endothelial cells, vascular smooth muscle cells, immune cells, and cardiomyocytes in varying proportions. Future studies using cardiomyocyte isolation, histological co-localization, or single-cell approaches will be necessary to define cell type-specific contributions more precisely.

Second, interpretation of band identities is limited by the nature of antibody-based detection. Band assignments were supported by expected molecular weight, manufacturer validation data, published reference information, and prior siRNA-based specificity testing for selected targets. However, direct molecular validation of individual immunoreactive species in the present tissue context was not performed. This is particularly relevant for proteins with multiple isoforms or precursor/processed forms, including IGF-1R, InsR, FGFR1, and TnI. Definitive distinction among isoforms, post-translationally modified variants, and processing states would require orthogonal validation approaches such as isoform-specific antibodies, deglycosylation analysis, immunoprecipitation, or mass spectrometry.

Third, the present study relies primarily on semi-quantitative immunoblotting and immunofluorescence. These methods provide robust descriptive information but do not replace direct functional assays of proliferation, regeneration, or pathway perturbation. Accordingly, the present data identify developmental associations and signaling patterns, but they do not establish which of these changes are causally required for regenerative competence.

Fourth, comparisons across postnatal stages are complicated by developmental changes in tissue composition. Neonatal and adult hearts differ substantially in myofibrillar density, extracellular matrix content, membrane composition, and relative proportions of non-myocyte populations. Although all samples were processed using the same high-strength extraction conditions, these compositional differences remain an inherent limitation when interpreting absolute protein abundance across ages.

Each biological replicate represents an individual animal (*n* = 4 per group). Although clear developmental trajectories are observed for many proteins, this sample size constrains statistical power and increases sensitivity to biological variability in certain targets, including A-Raf and some receptor measurements. We note that developmental variation in housekeeping protein expression, together with age-dependent differences in cardiac tissue composition, represents an inherent limitation for quantitative protein comparisons across postnatal stages. This dataset identifies developmental trends and candidate pathways but does not provide mechanistic proof.

**Figure 8:**
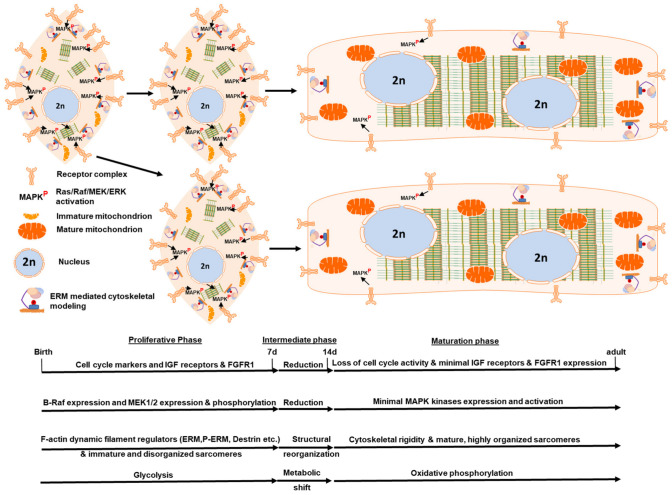
Cardiomyocyte-centered schematic of postnatal cardiac maturation. This schematic summarizes the principal developmental transitions identified in this study within a cardiomyocyte-centered framework across postnatal maturation. Three distinct phases are delineated: (i) Proliferative phase (P1–P7): Characterized by high expression of cell-cycle-associated genes and elevated levels of insulin-like growth factor (IGF) receptors and fibroblast growth factor receptor 1 (FGFR1), consistent with active proliferative signaling. In parallel, components of the MAPK cascade, including B-Raf and MEK1/2, display increased expression and phosphorylation. The cytoskeleton is dominated by a highly dynamic non-sarcomeric actin network, with abundant actin-binding and remodeling proteins (e.g., ERM proteins, phosphorylated ERM, destrin/cofilin), while sarcomeres remain immature and poorly organized. Cellular metabolism is predominantly glycolytic. (ii) Intermediate phase (P7–P14): Marked by a progressive reduction in cell-cycle-associated gene expression and decreased abundance of IGF receptors, FGFR1, and MAPK pathway components, consistent with attenuation of proliferative signaling. This phase is characterized by extensive cytoskeletal remodeling, including a transition from a dynamic non-sarcomeric actin network toward increased filament stabilization and progressive sarcomere assembly and alignment. In parallel, cardiomyocytes undergo a metabolic shift from glycolysis toward oxidative phosphorylation. (iii) Maturation phase (P14–adult): Defined by loss of cell-cycle activity and low levels of IGF receptors, FGFR1, and MAPK pathway components. The cytoskeleton is dominated by a stabilized, rigid architecture with reduced actin filament turnover and fully developed, highly organized sarcomeres. The non-sarcomeric actin network is markedly reduced, consistent with decreased cytoskeletal plasticity. Energy metabolism is primarily supported by mitochondrial oxidative phosphorylation. Together, this model highlights coordinated, but not necessarily causally linked, changes in proliferative signaling, MAPK pathway activity, cytoskeletal organization—particularly the transition from dynamic non-sarcomeric to stable sarcomeric structures—and metabolic maturation during postnatal cardiomyocyte development. This schematic represents a conceptual framework and does not imply that all measured signals originate exclusively from cardiomyocytes.

Taken together, these observations can be integrated into a schematic model of postnatal cardiomyocyte maturation (Figure 8), which highlights the coordinated progression of signaling, cytoskeletal, and metabolic changes. Early postnatal cardiomyocytes exhibit a profile consistent with proliferative competence, accompanied by a highly dynamic non-sarcomeric actin cytoskeleton and immature sarcomeric organization. This state transitions through an intermediate phase marked by attenuation of proliferative signatures and extensive cytoskeletal remodeling, most notably the shift from a dynamic actin network toward increased filament stabilization and progressive sarcomere assembly. In the mature state, cardiomyocytes display a rigid, highly ordered cytoskeletal architecture with reduced actin turnover and fully developed sarcomeres, alongside a predominantly oxidative metabolic profile. Importantly, this framework emphasizes that cytoskeletal remodeling-particularly the transition from non-sarcomeric to sarcomeric organization-represents a central and coordinated feature of postnatal maturation, rather than a secondary consequence of other processes.

## 5. Summary and Conclusions

In summary, our temporal profiling of postnatal mouse heart maturation identifies coordinated developmental changes at the levels of receptor-associated signaling, the Ras/Raf/MEK/ERK cascade, cytoskeletal organization, metabolism, and cell-cycle-associated proteins. As integrated in the schematic model (Figure 8), these changes reflect a transition from a neonatal state characterized by higher structural plasticity, a prominent non-sarcomeric actin cytoskeleton, and stronger growth factor receptor/MAPK-associated signaling toward a mature myocardial state with a stabilized, sarcomere-dominated architecture, increased oxidative capacity, and markedly reduced proliferative activity. Among these findings, the developmental decline in MEK1 Thr292-associated phosphorylation represents a notable and previously underexplored observation; however, its functional significance remains unresolved. These results define candidate developmental patterns associated with the closure of the neonatal regenerative window but do not distinguish causality. Notably, several aspects of this maturation program inversely resemble features observed in dedifferentiating or failing adult myocardium, supporting the concept that postnatally suppressed pathways may be re-engaged under pathological conditions. Defining whether and how such pathways can be modulated in a controlled and transient manner may be critical for future strategies aimed at restoring reparative competence without compromising myocardial integrity.

## Data Availability

The original contributions presented in this study are included in the article/Supplementary Materials. Further inquiries can be directed to the corresponding author.

## Supplementary Materials

The following supporting information can be downloaded at: https://www.mdpi.com/article/10.3390/cells15100873/s1, Table S1: One-way ANOVA analysis of IGF-1R expression. Table S2: One-way ANOVA analysis of InsR expression. Table S3: One-way ANOVA analysis of IGF-2R expression. Table S4: One-way ANOVA analysis of PDGFRb expression. Table S5: One-way ANOVA analysis of FGFR1 expression. Table S6: One-way ANOVA analysis of OSMR expression. Table S7: One-way ANOVA analysis of H/K/N Ras expression. Table S8: One-way ANOVA analysis of A-Raf expression. Table S9: One-way ANOVA analysis of B-Raf expression. Table S10: One-way ANOVA analysis of C-Raf expression. Table S11: One-way ANOVA analysis of MEK1 expression. Table S12: One-way ANOVA analysis of MEK2 expression. Table S13: One-way ANOVA analysis of MEK1/2 (S217/221) expression. Table S14: One-way ANOVA analysis of MEK1 (Thr292) expression. Table S15: One-way ANOVA analysis of Actn2 expression. Table S16: One-way ANOVA analysis of Sarcomeric actin expression. Table S17: One-way ANOVA analysis of TnC expression. Table S18: One-way ANOVA analysis of TnI expression. Table S19: One-way ANOVA analysis of TnT expression. Table S20: One-way ANOVA analysis of Actn1 expression. Table S21: One-way ANOVA analysis of Profilin 1 expression. Table S22: One-way ANOVA analysis of Cofilin 1 expression. Table S23: One-way ANOVA analysis of α-SMA expression. Table S24: One-way ANOVA analysis of Destrin expression. Table S25: One-way ANOVA analysis of Ezrin expression. Table S26: One-way ANOVA analysis of Radixin expression. Table S27: One-way ANOVA analysis of Moesin expression. Table S28: One-way ANOVA analysis of P-ERM expression. Table S29: One-way ANOVA analysis of Merlin expression. Table S30: One-way ANOVA analysis of Cytochrome c expression. Table S31: One-way ANOVA analysis of Mb expression. Table S32: One-way ANOVA analysis of P-H3 expression. Table S33: One-way ANOVA analysis of Aurora B expression. Figure S1: Uncropped Western blot corresponding to Figure 2B,G. Figure S2: Uncropped Western blot corresponding to Figure 3A. Figure S3: Uncropped Western blot corresponding to Figure 3F. Figure S4: Uncropped Western blot corresponding to Figure 4. Figure S5: Uncropped Western blot corresponding to Figure 5. Figure S6: Uncropped Western blot corresponding to Figure 6. Figure S7: Uncropped Western blot corresponding to Figure 7.
