# Supplementary material for: Coordinated Developmental Remodeling of IGF/FGF–MAPK Signaling and Cytoskeletal Plasticity Coincides with the Loss of Cardiac Regenerative Capacity
Source: Cells. 2026 May 11;15(10):873. doi: 10.3390/cells15100873 (PMC13204347; doi:10.3390/cells15100873)

IGF-1R	95 kDa					
Tukey's multiple comparisons test	Mean Diff,	95,00% CI of diff,	Significan	Summary	Adjusted P Value	
P3 vs. P7	42,5	18,18 to 66,82	Yes	***	0,0006	A-C
P3 vs. P14	75,25	50,93 to 99,57	Yes	****	<0,0001	A-E
P3 vs. P28	95,25	70,93 to 119,6	Yes	****	<0,0001	A-G
P3 vs. Adult	95,5	71,18 to 119,8	Yes	****	<0,0001	A-I
P7 vs. P14	32,75	8,426 to 57,07	Yes	**	0,0064	C-E
P7 vs. P28	52,75	28,43 to 77,07	Yes	****	<0,0001	C-G
P7 vs. Adult	53	28,68 to 77,32	Yes	****	<0,0001	C-I
P14 vs. P28	20	-4,324 to 44,32	No	ns	0,1337	E-G
P14 vs. Adult	20,25	-4,074 to 44,57	No	ns	0,1266	E-I
P28 vs. Adult	0,25	-24,07 to 24,57	No	ns	>0,9999	G-I

Test details	Mean 1	Mean 2	Mean Diff,	SE of diff,	n1	n2	q	DF
P3 vs. P7	100	57,5	42,5	7,877	4	4	7,63	15
P3 vs. P14	100	24,75	75,25	7,877	4	4	13,51	15
P3 vs. P28	100	4,75	95,25	7,877	4	4	17,1	15
P3 vs. Adult	100	4,5	95,5	7,877	4	4	17,15	15
P7 vs. P14	57,5	24,75	32,75	7,877	4	4	5,88	15
P7 vs. P28	57,5	4,75	52,75	7,877	4	4	9,47	15
P7 vs. Adult	57,5	4,5	53	7,877	4	4	9,515	15
P14 vs. P28	24,75	4,75	20	7,877	4	4	3,591	15
P14 vs. Adult	24,75	4,5	20,25	7,877	4	4	3,636	15
P28 vs. Adult	4,75	4,5	0,25	7,877	4	4	0,04488	15

	P3	P7	P14	P28	Adult
Number of values	4	4	4	4	4
Minimum	71	49	21	3	0
25% Percentile	78,25	49,75	21,25	3,5	0,75
Median	101,5	56	24,5	5	4,5
75% Percentile	120,3	66,75	28,5	5,75	8,25
Maximum	126	69	29	6	9
Mean	100	57,5	24,75	4,75	4,5
Std. Deviation	22,55	8,963	3,862	1,258	3,873
Std. Error of Mean	11,28	4,481	1,931	0,6292	1,936
Lower 95% CI	64,11	43,24	18,6	2,748	-1,663
Upper 95% CI	135,9	71,76	30,9	6,752	10,66

IGF-1R	200 kDa					
Tukey's multiple comparisons test	Mean Diff,	95,00% CI of diff,	Significan	Summary	Adjusted P Value	
P3 vs. P7	38,5	28,53 to 48,47	Yes	****	<0,0001	B-D
P3 vs. P14	77	67,03 to 86,97	Yes	****	<0,0001	B-F
P3 vs. P28	94,25	84,28 to 104,2	Yes	****	<0,0001	B-H
P3 vs. Adult	96,25	86,28 to 106,2	Yes	****	<0,0001	B-J
P7 vs. P14	38,5	28,53 to 48,47	Yes	****	<0,0001	D-F
P7 vs. P28	55,75	45,78 to 65,72	Yes	****	<0,0001	D-H
P7 vs. Adult	57,75	47,78 to 67,72	Yes	****	<0,0001	D-J
P14 vs. P28	17,25	7,284 to 27,22	Yes	***	0,0007	F-H
P14 vs. Adult	19,25	9,284 to 29,22	Yes	***	0,0002	F-J
P28 vs. Adult	2	-7,966 to 11,97	No	ns	0,9696	H-J

Test details	Mean 1	Mean 2	Mean Diff,	SE of diff,	n1	n2	q	DF
P3 vs. P7	100	61,5	38,5	3,227	4	4	16,87	15
P3 vs. P14	100	23	77	3,227	4	4	33,74	15
P3 vs. P28	100	5,75	94,25	3,227	4	4	41,3	15
P3 vs. Adult	100	3,75	96,25	3,227	4	4	42,17	15
P7 vs. P14	61,5	23	38,5	3,227	4	4	16,87	15
P7 vs. P28	61,5	5,75	55,75	3,227	4	4	24,43	15
P7 vs. Adult	61,5	3,75	57,75	3,227	4	4	25,3	15
P14 vs. P28	23	5,75	17,25	3,227	4	4	7,559	15
P14 vs. Adult	23	3,75	19,25	3,227	4	4	8,435	15
P28 vs. Adult	5,75	3,75	2	3,227	4	4	0,8764	15

	P3	P7	P14	P28	Adult
Number of values	4	4	4	4	4
Minimum	96	52	20	4	3
25% Percentile	96,75	53,5	20,25	4,25	3
Median	100	62	21	5	3,5
75% Percentile	103,3	69	27,75	8	4,75
Maximum	104	70	30	9	5
Mean	100	61,5	23	5,75	3,75
Std. Deviation	3,367	8,062	4,69	2,217	0,9574
Std. Error of Mean	1,683	4,031	2,345	1,109	0,4787
Lower 95% CI	94,64	48,67	15,54	2,222	2,227
Upper 95% CI	105,4	74,33	30,46	9,278	5,273

Table S1: One-way ANOVA analysis of IGF-1R expression.

InsR	95 kDa					
**Tukey's multiple comparisons test**	**Mean Diff,**	**95,00% CI of diff,**	**Significant?**	**Summary**	**Adjusted P Value**	
P3 vs. P7	-7,5	-25,84 to 10,84	No	ns	0,7163	A-C
P3 vs. P14	35	16,66 to 53,34	Yes	***	0,0002	A-E
P3 vs. P28	64,75	46,41 to 83,09	Yes	****	<0,0001	A-G
P3 vs. Adult	64	45,66 to 82,34	Yes	****	<0,0001	A-I
P7 vs. P14	42,5	24,16 to 60,84	Yes	****	<0,0001	C-E
P7 vs. P28	72,25	53,91 to 90,59	Yes	****	<0,0001	C-G
P7 vs. Adult	71,5	53,16 to 89,84	Yes	****	<0,0001	C-I
P14 vs. P28	29,75	11,41 to 48,09	Yes	**	0,0012	E-G
P14 vs. Adult	29	10,66 to 47,34	Yes	**	0,0016	E-I
P28 vs. Adult	-0,75	-19,09 to 17,59	No	ns	>0,9999	G-I

Test details	Mean 1	Mean 2	Mean Diff,	SE of diff,	n1	n2	q	DF
P3 vs. P7	100	107,5	-7,5	5,938	4	4	1,786	15
P3 vs. P14	100	65	35	5,938	4	4	8,336	15
P3 vs. P28	100	35,25	64,75	5,938	4	4	15,42	15
P3 vs. Adult	100	36	64	5,938	4	4	15,24	15
P7 vs. P14	107,5	65	42,5	5,938	4	4	10,12	15
P7 vs. P28	107,5	35,25	72,25	5,938	4	4	17,21	15
P7 vs. Adult	107,5	36	71,5	5,938	4	4	17,03	15
P14 vs. P28	65	35,25	29,75	5,938	4	4	7,086	15
P14 vs. Adult	65	36	29	5,938	4	4	6,907	15
P28 vs. Adult	35,25	36	-0,75	5,938	4	4	0,1786	15

	**3d**	**P3**	**P7**	**P14**	**P28**
Number of values	4	4	4	4	4
Minimum	91	99	61	21	30
25% Percentile	93	101,5	61,5	21,75	31,25
Median	100	109,5	64	35	36
75% Percentile	107	111,5	69,5	49	40,75
Maximum	109	112	71	50	42
Mean	100	107,5	65	35,25	36
Std. Deviation	7,394	5,802	4,32	14,86	4,967
Std. Error of Mean	3,697	2,901	2,16	7,432	2,483
Lower 95% CI	88,23	98,27	58,13	11,6	28,1
Upper 95% CI	111,8	116,7	71,87	58,9	43,9

InsR	220 kDa					
**Tukey's multiple comparisons test**	**Mean Diff,**	**95,00% CI of diff,**	**Significant?**	**Summary**	**Adjusted P Value**	
P3 vs. P7	-35,5	-90,63 to 19,63	No	ns	0,3173	B-D
P3 vs. P14	-6,75	-61,88 to 48,38	No	ns	0,9952	B-F
P3 vs. P28	55,5	0,3746 to 110,6	Yes	*	0,0481	B-H
P3 vs. Adult	67	11,87 to 122,1	Yes	*	0,0141	B-J
P7 vs. P14	28,75	-26,38 to 83,88	No	ns	0,5133	D-F
P7 vs. P28	91	35,87 to 146,1	Yes	**	0,0011	D-H
P7 vs. Adult	102,5	47,37 to 157,6	Yes	***	0,0003	D-J
P14 vs. P28	62,25	7,125 to 117,4	Yes	*	0,0235	F-H
P14 vs. Adult	73,75	18,62 to 128,9	Yes	**	0,0067	F-J
P28 vs. Adult	11,5	-43,63 to 66,63	No	ns	0,9651	H-J

Test details	Mean 1	Mean 2	Mean Diff,	SE of diff,	n1	n2	q	DF
P3 vs. P7	100	135,5	-35,5	17,85	4	4	2,812	15
P3 vs. P14	100	106,8	-6,75	17,85	4	4	0,5347	15
P3 vs. P28	100	44,5	55,5	17,85	4	4	4,397	15
P3 vs. Adult	100	33	67	17,85	4	4	5,308	15
P7 vs. P14	135,5	106,8	28,75	17,85	4	4	2,278	15
P7 vs. P28	135,5	44,5	91	17,85	4	4	7,209	15
P7 vs. Adult	135,5	33	102,5	17,85	4	4	8,12	15
P14 vs. P28	106,8	44,5	62,25	17,85	4	4	4,931	15
P14 vs. Adult	106,8	33	73,75	17,85	4	4	5,842	15
P28 vs. Adult	44,5	33	11,5	17,85	4	4	0,911	15

	**P3**	**P7**	**P14**	**P28**	**Adult**
Number of values	4	4	4	4	4
Minimum	82	130	92	12	8
25% Percentile	86,5	130	92,5	12	8,25
Median	100	134,5	98	37	27,5
75% Percentile	113,5	142	129,8	84,5	63,25
Maximum	118	143	139	92	69
Mean	100	135,5	106,8	44,5	33
Std. Deviation	14,7	6,557	21,93	39,48	29,81
Std. Error of Mean	7,348	3,279	10,96	19,74	14,91
Lower 95% CI	76,61	125,1	71,85	-18,31	-14,44
Upper 95% CI	123,4	145,9	141,6	107,3	80,44

Table S2: One-way ANOVA analysis of InsR expression.

IGF-2R

Tukey's multiple comparisons test	Mean Diff,	95,00% CI	Significant?	Summary	Adjusted P Value	
P3 vs. P7	42,25	47 to 58,03	Yes	****	<0,0001	A-B
P3 vs. P14	86,75	97 to 102,5	Yes	****	<0,0001	A-C
P3 vs. P28	95,25	47 to 111,0	Yes	****	<0,0001	A-D
P3 vs. Adult	97	22 to 112,8	Yes	****	<0,0001	A-E
P7 vs. P14	44,5	72 to 60,28	Yes	****	<0,0001	B-C
P7 vs. P28	53	22 to 68,78	Yes	****	<0,0001	B-D
P7 vs. Adult	54,75	97 to 70,53	Yes	****	<0,0001	B-E
P14 vs. P28	8,5	31 to 24,28	No	ns	0,4833	C-D
P14 vs. Adult	10,25	31 to 26,03	No	ns	0,3097	C-E
P28 vs. Adult	1,75	03 to 17,53	No	ns	0,9967	D-E

Test details	Mean 1	Mean 2	Mean Diff,	SE of diff,	n1	n2	q	DF
P3 vs. P7	98,75	56,5	42,25	5,11	4	4	11,69	15
P3 vs. P14	98,75	12	86,75	5,11	4	4	24,01	15
P3 vs. P28	98,75	3,5	95,25	5,11	4	4	26,36	15
P3 vs. Adult	98,75	1,75	97	5,11	4	4	26,84	15
P7 vs. P14	56,5	12	44,5	5,11	4	4	12,31	15
P7 vs. P28	56,5	3,5	53	5,11	4	4	14,67	15
P7 vs. Adult	56,5	1,75	54,75	5,11	4	4	15,15	15
P14 vs. P28	12	3,5	8,5	5,11	4	4	2,352	15
P14 vs. Adult	12	1,75	10,25	5,11	4	4	2,836	15
P28 vs. Adult	3,5	1,75	1,75	5,11	4	4	0,4843	15

	**P3**	**P7**	**P14**	**P28**	**Adult**
Number of values	4	4	4	4	4
Minimum	86	44	10	3	1
25% Percentile	88,25	48	10,25	3	1
Median	95,5	60	11,5	3,5	1,5
75% Percentile	112,5	61,5	14,25	4	2,75
Maximum	118	62	15	4	3
Mean	98,75	56,5	12	3,5	1,75
Std. Deviation	13,6	8,386	2,16	0,5774	0,9574
Std. Error of Mean	6,799	4,193	1,08	0,2887	0,4787
Lower 95% CI	77,11	43,16	8,563	2,581	0,2265
Upper 95% CI	120,4	69,84	15,44	4,419	3,273

Table S3: One-way ANOVA analysis of IGF-2R expression.

PDGFRb

Tukey's multiple comparisons test	Mean Diff,	95,00% CI of diff,	Significan	Summary	Adjusted P Value	
P3 vs. P7	-71,5	-114,1 to -28,87	Yes	***	0,0009	A-B
P3 vs. P14	-27,5	-70,13 to 15,13	No	ns	0,3158	A-C
P3 vs. P28	35,25	-7,384 to 77,88	No	ns	0,1305	A-D
P3 vs. Adult	53	10,37 to 95,63	Yes	*	0,0119	A-E
P7 vs. P14	44	1,366 to 86,63	Yes	*	0,0415	B-C
P7 vs. P28	106,8	64,12 to 149,4	Yes	****	<0,0001	B-D
P7 vs. Adult	124,5	81,87 to 167,1	Yes	****	<0,0001	B-E
P14 vs. P28	62,75	20,12 to 105,4	Yes	**	0,003	C-D
P14 vs. Adult	80,5	37,87 to 123,1	Yes	***	0,0003	C-E
P28 vs. Adult	17,75	-24,88 to 60,38	No	ns	0,7035	D-E

Test details	Mean 1	Mean 2	Mean Diff,	SE of diff,	n1	n2	q	DF
P3 vs. P7	100	171,5	-71,5	13,81	4	4	7,324	15
P3 vs. P14	100	127,5	-27,5	13,81	4	4	2,817	15
P3 vs. P28	100	64,75	35,25	13,81	4	4	3,611	15
P3 vs. Adult	100	47	53	13,81	4	4	5,429	15
P7 vs. P14	171,5	127,5	44	13,81	4	4	4,507	15
P7 vs. P28	171,5	64,75	106,8	13,81	4	4	10,93	15
P7 vs. Adult	171,5	47	124,5	13,81	4	4	12,75	15
P14 vs. P28	127,5	64,75	62,75	13,81	4	4	6,427	15
P14 vs. Adult	127,5	47	80,5	13,81	4	4	8,246	15
P28 vs. Adult	64,75	47	17,75	13,81	4	4	1,818	15

	P3	P7	P14	P28	Adult
Number of values	4	4	4	4	4
Minimum	72	149	105	46	36
25% Percentile	75,5	152,8	110,5	46,75	37,75
Median	100	170	127,5	64,5	46
75% Percentile	124,5	191,8	144,5	83	57,25
Maximum	128	197	150	84	60
Mean	100	171,5	127,5	64,75	47
Std. Deviation	25,56	20,27	18,38	20,02	10,17
Std. Error of Mean	12,78	10,14	9,188	10,01	5,083
Lower 95% CI	59,33	139,2	98,26	32,89	30,82
Upper 95% CI	140,7	203,8	156,7	96,61	63,18

Table S4: One-way ANOVA analysis of PDGFRb expression.

FGFR1	120 kDa					
**Tukey's multiple comparisons tes**	**Mean Diff,**	**95,00% CI of diff,**	**Significant?**	**Summary**	**Adjusted P Value**	
P3 vs. P7	-52,25	-109,1 to 4,577	No	ns	0,0789	A-C
P3 vs. P14	-10,25	-67,08 to 46,58	No	ns	0,9793	A-E
P3 vs. P28	58	1,173 to 114,8	Yes	*	0,0444	A-G
P3 vs. Adult	75,25	18,42 to 132,1	Yes	**	0,0073	A-I
P7 vs. P14	42	-14,83 to 98,83	No	ns	0,2041	C-E
P7 vs. P28	110,3	53,42 to 167,1	Yes	***	0,0002	C-G
P7 vs. Adult	127,5	70,67 to 184,3	Yes	****	<0,0001	C-I
P14 vs. P28	68,25	11,42 to 125,1	Yes	*	0,0153	E-G
P14 vs. Adult	85,5	28,67 to 142,3	Yes	**	0,0025	E-I
P28 vs. Adult	17,25	-39,58 to 74,08	No	ns	0,8779	G-I

Test details	Mean 1	Mean 2	Mean Diff,	SE of diff,	n1	n2	q	DF
P3 vs. P7	100	152,3	-52,25	18,4	4	4	4,015	15
P3 vs. P14	100	110,3	-10,25	18,4	4	4	0,7877	15
P3 vs. P28	100	42	58	18,4	4	4	4,457	15
P3 vs. Adult	100	24,75	75,25	18,4	4	4	5,783	15
P7 vs. P14	152,3	110,3	42	18,4	4	4	3,228	15
P7 vs. P28	152,3	42	110,3	18,4	4	4	8,472	15
P7 vs. Adult	152,3	24,75	127,5	18,4	4	4	9,798	15
P14 vs. P28	110,3	42	68,25	18,4	4	4	5,245	15
P14 vs. Adult	110,3	24,75	85,5	18,4	4	4	6,57	15
P28 vs. Adult	42	24,75	17,25	18,4	4	4	1,326	15

	**3d**	**P3**	**P7**	**P14**	**P28**
Number of values	4	4	4	4	4
Minimum	92	122	66	31	14
25% Percentile	93,25	126	72,75	31,75	15,75
Median	100	151	98	42,5	24
75% Percentile	106,8	179,8	160	51,75	34,5
Maximum	108	185	179	52	37
Mean	100	152,3	110,3	42	24,75
Std. Deviation	6,976	27,86	48,42	11,05	9,743
Std. Error of Mean	3,488	13,93	24,21	5,523	4,871
Lower 95% CI	88,9	107,9	33,2	24,42	9,247
Upper 95% CI	111,1	196,6	187,3	59,58	40,25

FGFR1	145 kDa					
**Tukey's multiple comparisons tes**	**Mean Diff,**	**95,00% CI of diff,**	**Significant?**	**Summary**	**Adjusted P Value**	
P3 vs. P7	-30,5	-117,3 to 56,32	No	ns	0,8115	B-D
P3 vs. P14	7,75	-79,07 to 94,57	No	ns	0,9986	B-F
P3 vs. P28	39,75	-47,07 to 126,6	No	ns	0,6287	B-H
P3 vs. Adult	75	-11,82 to 161,8	No	ns	0,1071	B-J
P7 vs. P14	38,25	-48,57 to 125,1	No	ns	0,6601	D-F
P7 vs. P28	70,25	-16,57 to 157,1	No	ns	0,1432	D-H
P7 vs. Adult	105,5	18,68 to 192,3	Yes	*	0,0141	D-J
P14 vs. P28	32	-54,82 to 118,8	No	ns	0,7844	F-H
P14 vs. Adult	67,25	-19,57 to 154,1	No	ns	0,171	F-J
P28 vs. Adult	35,25	-51,57 to 122,1	No	ns	0,7216	H-J

Test details	Mean 1	Mean 2	Mean Diff,	SE of diff,	n1	n2	q	DF
P3 vs. P7	100	130,5	-30,5	28,12	4	4	1,534	15
P3 vs. P14	100	92,25	7,75	28,12	4	4	0,3898	15
P3 vs. P28	100	60,25	39,75	28,12	4	4	1,999	15
P3 vs. Adult	100	25	75	28,12	4	4	3,773	15
P7 vs. P14	130,5	92,25	38,25	28,12	4	4	1,924	15
P7 vs. P28	130,5	60,25	70,25	28,12	4	4	3,534	15
P7 vs. Adult	130,5	25	105,5	28,12	4	4	5,307	15
P14 vs. P28	92,25	60,25	32	28,12	4	4	1,61	15
P14 vs. Adult	92,25	25	67,25	28,12	4	4	3,383	15
P28 vs. Adult	60,25	25	35,25	28,12	4	4	1,773	15

	**P3**	**P7**	**P14**	**P28**	**Adult**
Number of values	4	4	4	4	4
Minimum	65	95	47	17	7
25% Percentile	73,75	99	48,75	17,75	7,25
Median	100	123,5	88	59	11
75% Percentile	126,3	169	140	104	56,75
Maximum	135	180	146	106	71
Mean	100	130,5	92,25	60,25	25
Std. Deviation	28,58	37,06	49,28	48,33	30,82
Std. Error of Mean	14,29	18,53	24,64	24,17	15,41
Lower 95% CI	54,53	71,52	13,84	-16,66	-24,04
Upper 95% CI	145,5	189,5	170,7	137,2	74,04

Table S5: One-way ANOVA analysis of FGFR1 expression.

OSMR

Tukey's multiple comparisons test	Mean Diff,	95,00% CI of diff,	Significan	Summary	Adjusted P Value	
P3 vs. P7	-8,5	-46,25 to 29,25	No	ns	0,9544	A-B
P3 vs. P14	8,75	-29,00 to 46,50	No	ns	0,9496	A-C
P3 vs. P28	15,25	-22,50 to 53,00	No	ns	0,7252	A-D
P3 vs. Adult	25	-12,75 to 62,75	No	ns	0,2928	A-E
P7 vs. P14	17,25	-20,50 to 55,00	No	ns	0,6304	B-C
P7 vs. P28	23,75	-14,00 to 61,50	No	ns	0,3383	B-D
P7 vs. Adult	33,5	-4,254 to 71,25	No	ns	0,0942	B-E
P14 vs. P28	6,5	-31,25 to 44,25	No	ns	0,9826	C-D
P14 vs. Adult	16,25	-21,50 to 54,00	No	ns	0,6784	C-E
P28 vs. Adult	9,75	-28,00 to 47,50	No	ns	0,9274	D-E

Test details	Mean 1	Mean 2	Mean Diff,	SE of diff,	n1	n2	q	DF
P3 vs. P7	100	108,5	-8,5	12,23	4	4	0,9832	15
P3 vs. P14	100	91,25	8,75	12,23	4	4	1,012	15
P3 vs. P28	100	84,75	15,25	12,23	4	4	1,764	15
P3 vs. Adult	100	75	25	12,23	4	4	2,892	15
P7 vs. P14	108,5	91,25	17,25	12,23	4	4	1,995	15
P7 vs. P28	108,5	84,75	23,75	12,23	4	4	2,747	15
P7 vs. Adult	108,5	75	33,5	12,23	4	4	3,875	15
P14 vs. P28	91,25	84,75	6,5	12,23	4	4	0,7519	15
P14 vs. Adult	91,25	75	16,25	12,23	4	4	1,88	15
P28 vs. Adult	84,75	75	9,75	12,23	4	4	1,128	15

	P3	P7	P14	P28	Adult
Number of values	4	4	4	4	4
Minimum	88	92	70	67	70
25% Percentile	89,5	92	71,75	67,25	70,5
Median	100	105,5	88,5	80,5	72
75% Percentile	110,5	128	113,5	106,5	82,5
Maximum	112	131	118	111	86
Mean	100	108,5	91,25	84,75	75
Std. Deviation	10,95	19,67	21,96	21,23	7,394
Std. Error of Mean	5,477	9,836	10,98	10,62	3,697
Lower 95% CI	82,57	77,2	56,31	50,96	63,23
Upper 95% CI	117,4	139,8	126,2	118,5	86,77

Table S6: One-way ANOVA analysis of OSMR expression.

H/K/N Ras

Tukey's multiple comparisor	Mean Diff,	95,00% CI of diff,	Significan	Summary	Adjusted P Value	
P3 vs. P7	1,75	-13,84 to 17,34	No	ns	0,9965	A-B
P3 vs. P14	22	6,412 to 37,59	Yes	**	0,0043	A-C
P3 vs. P28	35,75	20,16 to 51,34	Yes	****	<0,0001	A-D
P3 vs. Adult	41,5	25,91 to 57,09	Yes	****	<0,0001	A-E
P7 vs. P14	20,25	4,662 to 35,84	Yes	**	0,0085	B-C
P7 vs. P28	34	18,41 to 49,59	Yes	****	<0,0001	B-D
P7 vs. Adult	39,75	24,16 to 55,34	Yes	****	<0,0001	B-E
P14 vs. P28	13,75	-1,838 to 29,34	No	ns	0,097	C-D
P14 vs. Adult	19,5	3,912 to 35,09	Yes	*	0,0114	C-E
P28 vs. Adult	5,75	-9,838 to 21,34	No	ns	0,784	D-E

Test details	Mean 1	Mean 2	Mean Diff,	SE of diff,	n1	n2	q	DF
P3 vs. P7	100	98,25	1,75	5,048	4	4	0,4903	15
P3 vs. P14	100	78	22	5,048	4	4	6,163	15
P3 vs. P28	100	64,25	35,75	5,048	4	4	10,02	15
P3 vs. Adult	100	58,5	41,5	5,048	4	4	11,63	15
P7 vs. P14	98,25	78	20,25	5,048	4	4	5,673	15
P7 vs. P28	98,25	64,25	34	5,048	4	4	9,525	15
P7 vs. Adult	98,25	58,5	39,75	5,048	4	4	11,14	15
P14 vs. P28	78	64,25	13,75	5,048	4	4	3,852	15
P14 vs. Adult	78	58,5	19,5	5,048	4	4	5,463	15
P28 vs. Adult	64,25	58,5	5,75	5,048	4	4	1,611	15

	P3	P7	P14	P28	Adult
Number of values	4	4	4	4	4
Minimum	89	88	72	61	49
25% Percentile	91,25	89,75	73,25	61,25	51,25
Median	100	97,5	78,5	63,5	59,5
75% Percentile	108,8	107,5	82,25	68	64,75
Maximum	111	110	83	69	66
Mean	100	98,25	78	64,25	58,5
Std. Deviation	9,129	9,251	4,69	3,594	7,141
Std. Error of Mean	4,564	4,626	2,345	1,797	3,571
Lower 95% CI	85,47	83,53	70,54	58,53	47,14
Upper 95% CI	114,5	113	85,46	69,97	69,86

Table S7: One-way ANOVA analysis of H/K/N Ras expression.

A-Raf

Tukey's multiple compa	Mean Diff,	95,00% CI of diff,	Significant	Summary	Adjusted P Value			
P3 vs. P7	-17,25	-222,5 to 188,0	No	ns	0,9989	A-B		
P3 vs. P14	-137,8	-343,0 to 67,50	No	ns	0,2812	A-C		
P3 vs. P28	69	-136,3 to 274,3	No	ns	0,834	A-D		
P3 vs. Adult	80	-125,3 to 285,3	No	ns	0,7495	A-E		
P7 vs. P14	-120,5	-325,8 to 84,75	No	ns	0,4022	B-C		
P7 vs. P28	86,25	-119,0 to 291,5	No	ns	0,6966	B-D		
P7 vs. Adult	97,25	-108,0 to 302,5	No	ns	0,5996	B-E		
P14 vs. P28	206,8	1,499 to 412,0	Yes	*	0,0479	C-D		
P14 vs. Adult	217,8	12,50 to 423,0	Yes	*	0,0351	C-E		
P28 vs. Adult	11	-194,3 to 216,3	No	ns	0,9998	D-E		
Test details	Mean 1	Mean 2	Mean Diff,	SE of diff,	n1	n2	q	DF
P3 vs. P7	125	142,3	-17,25	66,47	4	4	0,367	15
P3 vs. P14	125	262,8	-137,8	66,47	4	4	2,931	15
P3 vs. P28	125	56	69	66,47	4	4	1,468	15
P3 vs. Adult	125	45	80	66,47	4	4	1,702	15
P7 vs. P14	142,3	262,8	-120,5	66,47	4	4	2,564	15
P7 vs. P28	142,3	56	86,25	66,47	4	4	1,835	15
P7 vs. Adult	142,3	45	97,25	66,47	4	4	2,069	15
P14 vs. P28	262,8	56	206,8	66,47	4	4	4,399	15
P14 vs. Adult	262,8	45	217,8	66,47	4	4	4,633	15
P28 vs. Adult	56	45	11	66,47	4	4	0,234	15

	P3	P7	P14	P28	Adult
Number of values	4	4	4	4	4
Minimum	83	111	78	35	38
25% Percentile	91,5	118,3	84	37,25	38,25
Median	124	146	241,5	56,5	40,5
75% Percentile	159,5	162,5	462,8	74,25	56,25
Maximum	169	166	490	76	61
Mean	125	142,3	262,8	56	45
Std. Deviation	35,59	23,39	204,6	19,61	10,8
Std. Error of Mean	17,8	11,69	102,3	9,806	5,401
Lower 95% CI	68,37	105	-62,83	24,79	27,81
Upper 95% CI	181,6	179,5	588,3	87,21	62,19

Table S8: One-way ANOVA analysis of A-Raf expression.

B-Raf

Tukey's multiple comparisons test	Mean Diff,	95,00% CI of diff,	Significant	Summary	Adjusted P Value			
P3 vs. P7	-7	-23,88 to 9,878	No	ns	0,7063	A-B		
P3 vs. P14	41,25	24,37 to 58,13	Yes	****	<0,0001	A-C		
P3 vs. P28	88,25	71,37 to 105,1	Yes	****	<0,0001	A-D		
P3 vs. Adult	88,75	71,87 to 105,6	Yes	****	<0,0001	A-E		
P7 vs. P14	48,25	31,37 to 65,13	Yes	****	<0,0001	B-C		
P7 vs. P28	95,25	78,37 to 112,1	Yes	****	<0,0001	B-D		
P7 vs. Adult	95,75	78,87 to 112,6	Yes	****	<0,0001	B-E		
P14 vs. P28	47	30,12 to 63,88	Yes	****	<0,0001	C-D		
P14 vs. Adult	47,5	30,62 to 64,38	Yes	****	<0,0001	C-E		
P28 vs. Adult	0,5	-16,38 to 17,38	No	ns	>0,9999	D-E		
Test details	Mean 1	Mean 2	Mean Diff,	SE of diff,	n1	n2	q	DF
P3 vs. P7	100	107	-7	5,466	4	4	1,811	15
P3 vs. P14	100	58,75	41,25	5,466	4	4	10,67	15
P3 vs. P28	100	11,75	88,25	5,466	4	4	22,83	15
P3 vs. Adult	100	11,25	88,75	5,466	4	4	22,96	15
P7 vs. P14	107	58,75	48,25	5,466	4	4	12,48	15
P7 vs. P28	107	11,75	95,25	5,466	4	4	24,64	15
P7 vs. Adult	107	11,25	95,75	5,466	4	4	24,77	15
P14 vs. P28	58,75	11,75	47	5,466	4	4	12,16	15
P14 vs. Adult	58,75	11,25	47,5	5,466	4	4	12,29	15
P28 vs. Adult	11,75	11,25	0,5	5,466	4	4	0,1294	15

	P3	P7	P14	P28	Adult
Number of values	4	4	4	4	4
Minimum	88	104	40	9	8
25% Percentile	90	104,3	45,25	9,25	8,75
Median	100	107	62,5	12	11,5
75% Percentile	110	109,8	68,5	14	13,5
Maximum	112	110	70	14	14
Mean	100	107	58,75	11,75	11,25
Std. Deviation	10,33	2,944	13,05	2,63	2,5
Std. Error of Mean	5,164	1,472	6,524	1,315	1,25
Lower 95% CI	83,57	102,3	37,99	7,565	7,272
Upper 95% CI	116,4	111,7	79,51	15,93	15,23

Table S9: One-way ANOVA analysis of B-Raf expression.

C-Raf

Tukey's multiple comparisons test	Mean Diff,	95,00% CI of diff,	Significant?	Summary	Adjusted P Value			
P3 vs. P7	-14,25	-47,07 to 18,57	No	ns	0,6716	A-B		
P3 vs. P14	27,5	-5,318 to 60,32	No	ns	0,123	A-C		
P3 vs. P28	49	16,18 to 81,82	Yes	**	0,0027	A-D		
P3 vs. Adult	49,75	16,93 to 82,57	Yes	**	0,0023	A-E		
P7 vs. P14	41,75	8,932 to 74,57	Yes	*	0,01	B-C		
P7 vs. P28	63,25	30,43 to 96,07	Yes	***	0,0002	B-D		
P7 vs. Adult	64	31,18 to 96,82	Yes	***	0,0002	B-E		
P14 vs. P28	21,5	-11,32 to 54,32	No	ns	0,3021	C-D		
P14 vs. Adult	22,25	-10,57 to 55,07	No	ns	0,2726	C-E		
P28 vs. Adult	0,75	-32,07 to 33,57	No	ns	>0,9999	D-E		
Test details	Mean 1	Mean 2	Mean Diff,	SE of diff,	n1	n2	q	DF
P3 vs. P7	100	114,3	-14,25	10,63	4	4	1,896	15
P3 vs. P14	100	72,5	27,5	10,63	4	4	3,659	15
P3 vs. P28	100	51	49	10,63	4	4	6,52	15
P3 vs. Adult	100	50,25	49,75	10,63	4	4	6,62	15
P7 vs. P14	114,3	72,5	41,75	10,63	4	4	5,556	15
P7 vs. P28	114,3	51	63,25	10,63	4	4	8,417	15
P7 vs. Adult	114,3	50,25	64	10,63	4	4	8,516	15
P14 vs. P28	72,5	51	21,5	10,63	4	4	2,861	15
P14 vs. Adult	72,5	50,25	22,25	10,63	4	4	2,961	15
P28 vs. Adult	51	50,25	0,75	10,63	4	4	0,0998	15

	P3	P7	P14	P28	Adult
Number of values	4	4	4	4	4
Minimum	91	109	57	31	27
25% Percentile	91,5	110,3	57,5	36,25	30,5
Median	100	114,5	71,5	56	46
75% Percentile	108,5	118	88,5	60,75	74,25
Maximum	109	119	90	61	82
Mean	100	114,3	72,5	51	50,25
Std. Deviation	9,309	4,113	16,94	13,93	23,34
Std. Error of Mean	4,655	2,056	8,471	6,964	11,67
Lower 95% CI	85,19	107,7	45,54	28,84	13,11
Upper 95% CI	114,8	120,8	99,46	73,16	87,39

Table S10: One-way ANOVA analysis of C-Raf expression.

MEK1

Tukey's multiple comparisons test	Mean Diff,	95,00% CI of diff,	Significan	Summary	Adjusted P Value	
P3 vs. P7	12,75	-12,53 to 38,03	No	ns	0,544	A-B
P3 vs. P14	47,25	21,97 to 72,53	Yes	***	0,0003	A-C
P3 vs. P28	60,5	35,22 to 85,78	Yes	****	<0,0001	A-D
P3 vs. Adult	68	42,72 to 93,28	Yes	****	<0,0001	A-E
P7 vs. P14	34,5	9,221 to 59,78	Yes	**	0,0057	B-C
P7 vs. P28	47,75	22,47 to 73,03	Yes	***	0,0003	B-D
P7 vs. Adult	55,25	29,97 to 80,53	Yes	****	<0,0001	B-E
P14 vs. P28	13,25	-12,03 to 38,53	No	ns	0,5087	C-D
P14 vs. Adult	20,75	-4,529 to 46,03	No	ns	0,1347	C-E
P28 vs. Adult	7,5	-17,78 to 32,78	No	ns	0,8863	D-E

Test details	Mean 1	Mean 2	Mean Diff,	SE of diff,	n1	n2	q	DF
P3 vs. P7	100	87,25	12,75	8,186	4	4	2,203	15
P3 vs. P14	100	52,75	47,25	8,186	4	4	8,163	15
P3 vs. P28	100	39,5	60,5	8,186	4	4	10,45	15
P3 vs. Adult	100	32	68	8,186	4	4	11,75	15
P7 vs. P14	87,25	52,75	34,5	8,186	4	4	5,96	15
P7 vs. P28	87,25	39,5	47,75	8,186	4	4	8,249	15
P7 vs. Adult	87,25	32	55,25	8,186	4	4	9,545	15
P14 vs. P28	52,75	39,5	13,25	8,186	4	4	2,289	15
P14 vs. Adult	52,75	32	20,75	8,186	4	4	3,585	15
P28 vs. Adult	39,5	32	7,5	8,186	4	4	1,296	15

	P3	P7	P14	P28	Adult
Number of values	4	4	4	4	4
Minimum	91	67	51	35	23
25% Percentile	93,25	67,75	51,25	35,25	24
Median	101	84	53	38,5	30,5
75% Percentile	105,8	110	54	44,75	41,5
Maximum	107	114	54	46	44
Mean	100	87,25	52,75	39,5	32
Std. Deviation	6,683	22,65	1,5	5,066	9,201
Std. Error of Mean	3,342	11,32	0,75	2,533	4,601
Lower 95% CI	89,37	51,21	50,36	31,44	17,36
Upper 95% CI	110,6	123,3	55,14	47,56	46,64

Table S11: One-way ANOVA analysis of MEK1 expression.

Tukey's multiple comparisons test	Mean Diff,	95,00% CI of diff,	Significan	Summary	Adjusted P Value	
P3 vs. P7	-28,25	-68,72 to 12,22	No	ns	0,2484	A-B
P3 vs. P14	33	-7,467 to 73,47	No	ns	0,1385	A-C
P3 vs. P28	78,75	38,28 to 119,2	Yes	***	0,0002	A-D
P3 vs. Adult	91,5	51,03 to 132,0	Yes	****	<0,0001	A-E
P7 vs. P14	61,25	20,78 to 101,7	Yes	**	0,0024	B-C
P7 vs. P28	107	66,53 to 147,5	Yes	****	<0,0001	B-D
P7 vs. Adult	119,8	79,28 to 160,2	Yes	****	<0,0001	B-E
P14 vs. P28	45,75	5,283 to 86,22	Yes	*	0,0233	C-D
P14 vs. Adult	58,5	18,03 to 98,97	Yes	**	0,0035	C-E
P28 vs. Adult	12,75	-27,72 to 53,22	No	ns	0,8631	D-E

Test details	Mean 1	Mean 2	Mean Diff,	SE of diff,	n1	n2	q	DF
P3 vs. P7	99,75	128	-28,25	13,11	4	4	3,049	15
P3 vs. P14	99,75	66,75	33	13,11	4	4	3,561	15
P3 vs. P28	99,75	21	78,75	13,11	4	4	8,498	15
P3 vs. Adult	99,75	8,25	91,5	13,11	4	4	9,874	15
P7 vs. P14	128	66,75	61,25	13,11	4	4	6,61	15
P7 vs. P28	128	21	107	13,11	4	4	11,55	15
P7 vs. Adult	128	8,25	119,8	13,11	4	4	12,92	15
P14 vs. P28	66,75	21	45,75	13,11	4	4	4,937	15
P14 vs. Adult	66,75	8,25	58,5	13,11	4	4	6,313	15
P28 vs. Adult	21	8,25	12,75	13,11	4	4	1,376	15

	P3	P7	P14	P28	Adult
Number of values	4	4	4	4	4
Minimum	79	101	47	14	5
25% Percentile	80,25	104	50,5	14,25	5,75
Median	92	129,5	63,5	20,5	8,5
75% Percentile	127	150,5	86,25	28,25	10,5
Maximum	136	152	93	29	11
Mean	99,75	128	66,75	21	8,25
Std. Deviation	25,77	24,86	19,26	7,616	2,5
Std. Error of Mean	12,89	12,43	9,63	3,808	1,25
Lower 95% CI	58,74	88,44	36,1	8,882	4,272
Upper 95% CI	140,8	167,6	97,4	33,12	12,23

Table S12: One-way ANOVA analysis of MEK2 expression.

P-MEK 1/2 (S217/221)

Tukey's multiple comparisons test	Mean Diff,	95,00% CI of diff,	Significant	Summary	Adjusted P Value			
P3 vs. P7	46,25	24,51 to 67,99	Yes	****	<0,0001	A-B		
P3 vs. P14	83,25	61,51 to 105,0	Yes	****	<0,0001	A-C		
P3 vs. P28	89,5	67,76 to 111,2	Yes	****	<0,0001	A-D		
P3 vs. Adult	92	70,26 to 113,7	Yes	****	<0,0001	A-E		
P7 vs. P14	37	15,26 to 58,74	Yes	***	0,0008	B-C		
P7 vs. P28	43,25	21,51 to 64,99	Yes	***	0,0002	B-D		
P7 vs. Adult	45,75	24,01 to 67,49	Yes	****	<0,0001	B-E		
P14 vs. P28	6,25	-15,49 to 27,99	No	ns	0,897	C-D		
P14 vs. Adult	8,75	-12,99 to 30,49	No	ns	0,7276	C-E		
P28 vs. Adult	2,5	-19,24 to 24,24	No	ns	0,9962	D-E		
Test details	Mean 1	Mean 2	Mean Diff,	SE of diff,	n1	n2	q	DF
P3 vs. P7	100	53,75	46,25	7,039	4	4	9,292	15
P3 vs. P14	100	16,75	83,25	7,039	4	4	16,73	15
P3 vs. P28	100	10,5	89,5	7,039	4	4	17,98	15
P3 vs. Adult	100	8	92	7,039	4	4	18,48	15
P7 vs. P14	53,75	16,75	37	7,039	4	4	7,434	15
P7 vs. P28	53,75	10,5	43,25	7,039	4	4	8,689	15
P7 vs. Adult	53,75	8	45,75	7,039	4	4	9,191	15
P14 vs. P28	16,75	10,5	6,25	7,039	4	4	1,256	15
P14 vs. Adult	16,75	8	8,75	7,039	4	4	1,758	15
P28 vs. Adult	10,5	8	2,5	7,039	4	4	0,5023	15

	P3	P7	P14	P28	Adult
Number of values	4	4	4	4	4
Minimum	80	46	13	5	6
25% Percentile	81,75	46,5	13,5	5,5	6
Median	100	52,5	16,5	10,5	7,5
75% Percentile	118,3	62,25	20,25	15,5	10,5
Maximum	120	64	21	16	11
Mean	100	53,75	16,75	10,5	8
Std. Deviation	19,48	8,342	3,5	5,323	2,449
Std. Error of Mean	9,738	4,171	1,75	2,661	1,225
Lower 95% CI	69,01	40,48	11,18	2,03	4,102
Upper 95% CI	131	67,02	22,32	18,97	11,9

Table S13: One-way ANOVA analysis of MEK1/2 (S217/221) expression.

P-MEK 1 (Thr292)

Tukey's multiple comparisons tes	Mean Diff,	95,00% CI of diff,	Significant	Summary	Adjusted P Value			
P3 vs. P7	32,25	20,47 to 44,03	Yes	****	<0,0001	A-B		
P3 vs. P14	91,75	79,97 to 103,5	Yes	****	<0,0001	A-C		
P3 vs. P28	92,25	80,47 to 104,0	Yes	****	<0,0001	A-D		
P3 vs. Adult	95	83,22 to 106,8	Yes	****	<0,0001	A-E		
P7 vs. P14	59,5	47,72 to 71,28	Yes	****	<0,0001	B-C		
P7 vs. P28	60	48,22 to 71,78	Yes	****	<0,0001	B-D		
P7 vs. Adult	62,75	50,97 to 74,53	Yes	****	<0,0001	B-E		
P14 vs. P28	0,5	-11,28 to 12,28	No	ns	>0,9999	C-D		
P14 vs. Adult	3,25	-8,525 to 15,03	No	ns	0,9097	C-E		
P28 vs. Adult	2,75	-9,025 to 14,53	No	ns	0,9483	D-E		
Test details	Mean 1	Mean 2	Mean Diff,	SE of diff,	n1	n2	q	DF
P3 vs. P7	100	67,75	32,25	3,813	4	4	11,96	15
P3 vs. P14	100	8,25	91,75	3,813	4	4	34,03	15
P3 vs. P28	100	7,75	92,25	3,813	4	4	34,21	15
P3 vs. Adult	100	5	95	3,813	4	4	35,23	15
P7 vs. P14	67,75	8,25	59,5	3,813	4	4	22,07	15
P7 vs. P28	67,75	7,75	60	3,813	4	4	22,25	15
P7 vs. Adult	67,75	5	62,75	3,813	4	4	23,27	15
P14 vs. P28	8,25	7,75	0,5	3,813	4	4	0,1854	15
P14 vs. Adult	8,25	5	3,25	3,813	4	4	1,205	15
P28 vs. Adult	7,75	5	2,75	3,813	4	4	1,02	15

	P3	P7	P14	P28	Adult
Number of values	4	4	4	4	4
Minimum	96	56	7	6	4
25% Percentile	96,75	57	7,25	6,25	4
Median	100	68,5	8,5	8	5
75% Percentile	103,3	77,75	9	9	6
Maximum	104	78	9	9	6
Mean	100	67,75	8,25	7,75	5
Std. Deviation	3,367	11,38	0,9574	1,5	1,155
Std. Error of Mean	1,683	5,692	0,4787	0,75	0,5774
Lower 95% CI	94,64	49,64	6,727	5,363	3,163
Upper 95% CI	105,4	85,86	9,773	10,14	6,837

Table S14: One-way ANOVA analysis of MEK1 (Thr292) expression.

Actn2

Tukey's multiple comparisons test	Mean Diff,	95,00% CI of diff,	Significant?	Summary	Adjusted P Value	
P3 vs. P7	16,25	-39,28 to 71,78	No	ns	0,8911	A-B
P3 vs. P14	17	-38,53 to 72,53	No	ns	0,8746	A-C
P3 vs. P28	2,25	-53,28 to 57,78	No	ns	>0,9999	A-D
P3 vs. Adult	19,75	-35,78 to 75,28	No	ns	0,8048	A-E
P7 vs. P14	0,75	-54,78 to 56,28	No	ns	>0,9999	B-C
P7 vs. P28	-14	-69,53 to 41,53	No	ns	0,9329	B-D
P7 vs. Adult	3,5	-52,03 to 59,03	No	ns	0,9996	B-E
P14 vs. P28	-14,75	-70,28 to 40,78	No	ns	0,9203	C-D
P14 vs. Adult	2,75	-52,78 to 58,28	No	ns	0,9999	C-E
P28 vs. Adult	17,5	-38,03 to 73,03	No	ns	0,863	D-E

Test details	Mean 1	Mean 2	Mean Diff,	SE of diff,	n1	n2	q	DF
P3 vs. P7	100	83,75	16,25	17,98	4	4	1,278	15
P3 vs. P14	100	83	17	17,98	4	4	1,337	15
P3 vs. P28	100	97,75	2,25	17,98	4	4	0,177	15
P3 vs. Adult	100	80,25	19,75	17,98	4	4	1,553	15
P7 vs. P14	83,75	83	0,75	17,98	4	4	0,05899	15
P7 vs. P28	83,75	97,75	-14	17,98	4	4	1,101	15
P7 vs. Adult	83,75	80,25	3,5	17,98	4	4	0,2753	15
P14 vs. P28	83	97,75	-14,75	17,98	4	4	1,16	15
P14 vs. Adult	83	80,25	2,75	17,98	4	4	0,2163	15
P28 vs. Adult	97,75	80,25	17,5	17,98	4	4	1,376	15

	P3	P7	P14	P28	Adult
Number of values	4	4	4	4	4
Minimum	79	56	62	67	57
25% Percentile	83	56,5	66,25	69,5	58,25
Median	100	80	81,5	100	75,5
75% Percentile	117	114,8	101,3	123,8	107
Maximum	121	119	107	124	113
Mean	100	83,75	83	97,75	80,25
Std. Deviation	17,63	31,67	18,57	30,02	25,97
Std. Error of Mean	8,813	15,83	9,283	15,01	12,98
Lower 95% CI	71,95	33,36	53,46	49,99	38,93
Upper 95% CI	128	134,1	112,5	145,5	121,6

Table S15: One-way ANOVA analysis of Actn2 expression.

Sarcomeric Actin

Tukey's multiple comparisons test	Mean Diff,	95,00% CI of diff,	Significant?	Summary	Adjusted P Value			
P3 vs. P7	-13,75	-44,12 to 16,62	No	ns	0,6381	A-B		
P3 vs. P14	-25	-55,37 to 5,373	No	ns	0,1331	A-C		
P3 vs. P28	-36,5	-66,87 to -6,127	Yes	*	0,0153	A-D		
P3 vs. Adult	-39,75	-70,12 to -9,377	Yes	**	0,008	A-E		
P7 vs. P14	-11,25	-41,62 to 19,12	No	ns	0,7815	B-C		
P7 vs. P28	-22,75	-53,12 to 7,623	No	ns	0,1943	B-D		
P7 vs. Adult	-26	-56,37 to 4,373	No	ns	0,1117	B-E		
P14 vs. P28	-11,5	-41,87 to 18,87	No	ns	0,7681	C-D		
P14 vs. Adult	-14,75	-45,12 to 15,62	No	ns	0,578	C-E		
P28 vs. Adult	-3,25	-33,62 to 27,12	No	ns	0,9971	D-E		
Test details	Mean 1	Mean 2	Mean Diff,	SE of diff,	n1	n2	q	DF
P3 vs. P7	100	113,8	-13,75	9,836	4	4	1,977	15
P3 vs. P14	100	125	-25	9,836	4	4	3,594	15
P3 vs. P28	100	136,5	-36,5	9,836	4	4	5,248	15
P3 vs. Adult	100	139,8	-39,75	9,836	4	4	5,715	15
P7 vs. P14	113,8	125	-11,25	9,836	4	4	1,617	15
P7 vs. P28	113,8	136,5	-22,75	9,836	4	4	3,271	15
P7 vs. Adult	113,8	139,8	-26	9,836	4	4	3,738	15
P14 vs. P28	125	136,5	-11,5	9,836	4	4	1,653	15
P14 vs. Adult	125	139,8	-14,75	9,836	4	4	2,121	15
P28 vs. Adult	136,5	139,8	-3,25	9,836	4	4	0,4673	15

	P3	P7	P14	P28	Adult
Number of values	4	4	4	4	4
Minimum	93	94	109	128	133
25% Percentile	94	94,75	109,3	128,3	134,8
Median	100	111,5	123,5	134	140,5
75% Percentile	106	135	142,3	147,3	144
Maximum	107	138	144	150	145
Mean	100	113,8	125	136,5	139,8
Std. Deviation	6,218	21,67	18,13	10,28	4,992
Std. Error of Mean	3,109	10,83	9,065	5,14	2,496
Lower 95% CI	90,11	79,27	96,15	120,1	131,8
Upper 95% CI	109,9	148,2	153,8	152,9	147,7

Table S16: One-way ANOVA analysis of Sarcomeric actin expression.

TnC									
**Tukey's multiple comparisons test**	**Mean Diff,**	**95,00% CI of diff,**	**Significant?**	**Summary**	**Adjusted P Value**				
P3 vs. P7	4,5	-20,69 to 29,69	No	ns	0,9801	A-B			
P3 vs. P14	-28,25	-53,44 to -3,059	Yes	*	0,0246	A-C			
P3 vs. P28	-36	-61,19 to -10,81	Yes	**	0,0039	A-D			
P3 vs. Adult	-24,25	-49,44 to 0,9407	No	ns	0,0619	A-E			
P7 vs. P14	-32,75	-57,94 to -7,559	Yes	**	0,0085	B-C			
P7 vs. P28	-40,5	-65,69 to -15,31	Yes	**	0,0014	B-D			
P7 vs. Adult	-28,75	-53,94 to -3,559	Yes	*	0,0219	B-E			
P14 vs. P28	-7,75	-32,94 to 17,44	No	ns	0,8727	C-D			
P14 vs. Adult	4	-21,19 to 29,19	No	ns	0,9871	C-E			
P28 vs. Adult	11,75	-13,44 to 36,94	No	ns	0,613	D-E			
Test details	Mean 1	Mean 2	Mean Diff,	SE of diff,	n1	n2	q	DF	
P3 vs. P7	100	95,5	4,5	8,158	4	4	0,7801	15	
P3 vs. P14	100	128,3	-28,25	8,158	4	4	4,897	15	
P3 vs. P28	100	136	-36	8,158	4	4	6,241	15	
P3 vs. Adult	100	124,3	-24,25	8,158	4	4	4,204	15	
P7 vs. P14	95,5	128,3	-32,75	8,158	4	4	5,677	15	
P7 vs. P28	95,5	136	-40,5	8,158	4	4	7,021	15	
P7 vs. Adult	95,5	124,3	-28,75	8,158	4	4	4,984	15	
P14 vs. P28	128,3	136	-7,75	8,158	4	4	1,344	15	
P14 vs. Adult	128,3	124,3	4	8,158	4	4	0,6934	15	
P28 vs. Adult	136	124,3	11,75	8,158	4	4	2,037	15	

	**P3**	**P7**	**P14**	**P28**	**Adult**
Number of values	4	4	4	4	4
Minimum	96	88	109	123	114
25% Percentile	96	88,5	112,3	124,3	114,8
Median	100	95,5	126	137	123,5
75% Percentile	104	102,5	146,5	146,8	134,5
Maximum	104	103	152	147	136
Mean	100	95,5	128,3	136	124,3
Std. Deviation	4,619	7,594	18,04	12,3	10,47
Std. Error of Mean	2,309	3,797	9,022	6,151	5,234
Lower 95% CI	92,65	83,42	99,54	116,4	107,6
Upper 95% CI	107,3	107,6	157	155,6	140,9

Table S17: One-way ANOVA analysis of TnC expression.

TnI								
**Tukey's multiple comparisons test**	**Mean Diff,**	**95,00% CI of diff,**	**Significant?**	**Summary**	**Adjusted P Value**			
P3 vs. P7	-145,3	-819,0 to 528,5	No	ns	0,9608	A-B		
P3 vs. P14	-819,8	-1493 to -146,0	Yes	*	0,0139	A-C		
P3 vs. P28	-1064	-1738 to -390,3	Yes	**	0,0016	A-D		
P3 vs. Adult	-971	-1645 to -297,3	Yes	**	0,0036	A-E		
P7 vs. P14	-674,5	-1348 to -0,7790	Yes	*	0,0497	B-C		
P7 vs. P28	-918,8	-1592 to -245,0	Yes	**	0,0058	B-D		
P7 vs. Adult	-825,8	-1499 to -152,0	Yes	*	0,0132	B-E		
P14 vs. P28	-244,3	-918,0 to 429,5	No	ns	0,7941	C-D		
P14 vs. Adult	-151,3	-825,0 to 522,5	No	ns	0,9549	C-E		
P28 vs. Adult	93	-580,7 to 766,7	No	ns	0,9924	D-E		
Test details	Mean 1	Mean 2	Mean Diff,	SE of diff,	n1	n2	q	DF
P3 vs. P7	102	247,3	-145,3	218,2	4	4	0,9415	15
P3 vs. P14	102	921,8	-819,8	218,2	4	4	5,314	15
P3 vs. P28	102	1166	-1064	218,2	4	4	6,897	15
P3 vs. Adult	102	1073	-971	218,2	4	4	6,294	15
P7 vs. P14	247,3	921,8	-674,5	218,2	4	4	4,372	15
P7 vs. P28	247,3	1166	-918,8	218,2	4	4	5,955	15
P7 vs. Adult	247,3	1073	-825,8	218,2	4	4	5,352	15
P14 vs. P28	921,8	1166	-244,3	218,2	4	4	1,583	15
P14 vs. Adult	921,8	1073	-151,3	218,2	4	4	0,9804	15
P28 vs. Adult	1166	1073	93	218,2	4	4	0,6028	15

	**P3**	**P7**	**P14**	**P28**	**Adult**
Number of values	4	4	4	4	4
Minimum	85	195	670	811	761
25% Percentile	88,5	208	692,3	841,3	780,5
Median	103,5	253	876,5	1051	873
75% Percentile	114	280,8	1197	1606	1566
Maximum	116	288	1264	1752	1785
Mean	102	247,3	921,8	1166	1073
Std. Deviation	13,29	38,85	266	418	478,4
Std. Error of Mean	6,646	19,43	133	209	239,2
Lower 95% CI	80,85	185,4	498,5	500,9	311,8
Upper 95% CI	123,1	309,1	1345	1831	1834

Table S18: One-way ANOVA analysis of TnI expression.

TnT								
**Tukey's multiple comparisons test**	**Mean Diff,**	**95,00% CI of di**	**Significant?**	**Summary**	**Adjusted P Value**			
P3 vs. P7	-30,75	-96,28 to 34,78	No	ns	0,6079	A-B		
P3 vs. P14	-39,5	-105,0 to 26,03	No	ns	0,3775	A-C		
P3 vs. P28	-23,75	-89,28 to 41,78	No	ns	0,7942	A-D		
P3 vs. Adult	-50,75	-116,3 to 14,78	No	ns	0,1711	A-E		
P7 vs. P14	-8,75	-74,28 to 56,78	No	ns	0,9933	B-C		
P7 vs. P28	7	-58,53 to 72,53	No	ns	0,9971	B-D		
P7 vs. Adult	-20	-85,53 to 45,53	No	ns	0,8758	B-E		
P14 vs. P28	15,75	-49,78 to 81,28	No	ns	0,9429	C-D		
P14 vs. Adult	-11,25	-76,78 to 54,28	No	ns	0,9828	C-E		
P28 vs. Adult	-27	-92,53 to 38,53	No	ns	0,7111	D-E		
Test details	Mean 1	Mean 2	Mean Diff,	SE of diff,	n1	n2	q	DF
P3 vs. P7	100	130,8	-30,75	21,22	4	4	2,049	15
P3 vs. P14	100	139,5	-39,5	21,22	4	4	2,632	15
P3 vs. P28	100	123,8	-23,75	21,22	4	4	1,583	15
P3 vs. Adult	100	150,8	-50,75	21,22	4	4	3,382	15
P7 vs. P14	130,8	139,5	-8,75	21,22	4	4	0,5831	15
P7 vs. P28	130,8	123,8	7	21,22	4	4	0,4665	15
P7 vs. Adult	130,8	150,8	-20	21,22	4	4	1,333	15
P14 vs. P28	139,5	123,8	15,75	21,22	4	4	1,05	15
P14 vs. Adult	139,5	150,8	-11,25	21,22	4	4	0,7497	15
P28 vs. Adult	123,8	150,8	-27	21,22	4	4	1,799	15

	**P3**	**P7**	**P14**	**P28**	**Adult**
Number of values	4	4	4	4	4
Minimum	62	124	122	104	111
25% Percentile	71	124,5	123,3	105,3	111,3
Median	100	129	134	111,5	144,5
75% Percentile	129	138,8	161,3	154,5	196,5
Maximum	138	141	168	168	203
Mean	100	130,8	139,5	123,8	150,8
Std. Deviation	31,07	7,632	20,63	29,78	46,55
Std. Error of Mean	15,53	3,816	10,32	14,89	23,28
Lower 95% CI	50,56	118,6	106,7	76,36	76,68
Upper 95% CI	149,4	142,9	172,3	171,1	224,8

Table S19: One-way ANOVA analysis of TnT expression.

Actn1

Tukey's multiple comparisons test	Mean Diff,	95,00% CI of diff,	Significant?	Summary	Adjusted P Value			
P3 vs. P7	-15,5	-42,98 to 11,98	No	ns	0,4399	A-B		
P3 vs. P14	35	7,521 to 62,48	Yes	**	0,0099	A-C		
P3 vs. P28	84	56,52 to 111,5	Yes	****	<0,0001	A-D		
P3 vs. Adult	89,75	62,27 to 117,2	Yes	****	<0,0001	A-E		
P7 vs. P14	50,5	23,02 to 77,98	Yes	***	0,0004	B-C		
P7 vs. P28	99,5	72,02 to 127,0	Yes	****	<0,0001	B-D		
P7 vs. Adult	105,3	77,77 to 132,7	Yes	****	<0,0001	B-E		
P14 vs. P28	49	21,52 to 76,48	Yes	***	0,0005	C-D		
P14 vs. Adult	54,75	27,27 to 82,23	Yes	***	0,0002	C-E		
P28 vs. Adult	5,75	-21,73 to 33,23	No	ns	0,9647	D-E		
Test details	Mean 1	Mean 2	Mean Diff,	SE of diff,	n1	n2	q	DF
P3 vs. P7	101,5	117	-15,5	8,899	4	4	2,463	15
P3 vs. P14	101,5	66,5	35	8,899	4	4	5,562	15
P3 vs. P28	101,5	17,5	84	8,899	4	4	13,35	15
P3 vs. Adult	101,5	11,75	89,75	8,899	4	4	14,26	15
P7 vs. P14	117	66,5	50,5	8,899	4	4	8,025	15
P7 vs. P28	117	17,5	99,5	8,899	4	4	15,81	15
P7 vs. Adult	117	11,75	105,3	8,899	4	4	16,73	15
P14 vs. P28	66,5	17,5	49	8,899	4	4	7,787	15
P14 vs. Adult	66,5	11,75	54,75	8,899	4	4	8,701	15
P28 vs. Adult	17,5	11,75	5,75	8,899	4	4	0,9138	15

	P3	P7	P14	P28	Adult
Number of values	4	4	4	4	4
Minimum	86	98	55	9	6
25% Percentile	88	101,8	57	11,25	6
Median	95	117,5	68,5	19	10,5
75% Percentile	121,5	131,8	74	22,25	18,75
Maximum	130	135	74	23	20
Mean	101,5	117	66,5	17,5	11,75
Std. Deviation	19,49	15,56	9,256	6,028	6,946
Std. Error of Mean	9,743	7,778	4,628	3,014	3,473
Lower 95% CI	70,49	92,25	51,77	7,909	0,697
Upper 95% CI	132,5	141,8	81,23	27,09	22,8

Table S20: One-way ANOVA analysis of Actn1 expression.

Profilin 1

Tukey's multiple comparisons test	Mean Diff,	95,00% CI	Significant	Summary	Adjusted P Value	
P3 vs. P7	19,5	79 to 39,20	No	ns	0,053	A-B
P3 vs. P14	82	30 to 101,7	Yes	****	<0,0001	A-C
P3 vs. P28	94,75	05 to 114,4	Yes	****	<0,0001	A-D
P3 vs. Adult	94,5	80 to 114,2	Yes	****	<0,0001	A-E
P7 vs. P14	62,5	80 to 82,20	Yes	****	<0,0001	B-C
P7 vs. P28	75,25	55 to 94,95	Yes	****	<0,0001	B-D
P7 vs. Adult	75	30 to 94,70	Yes	****	<0,0001	B-E
P14 vs. P28	12,75	48 to 32,45	No	ns	0,3127	C-D
P14 vs. Adult	12,5	98 to 32,20	No	ns	0,3304	C-E
P28 vs. Adult	-0,25	95 to 19,45	No	ns	>0,9999	D-E

Test details	Mean 1	Mean 2	Mean Diff,	SE of diff,	n1	n2	q	DF
P3 vs. P7	102	82,5	19,5	6,379	4	4	4,323	15
P3 vs. P14	102	20	82	6,379	4	4	18,18	15
P3 vs. P28	102	7,25	94,75	6,379	4	4	21,01	15
P3 vs. Adult	102	7,5	94,5	6,379	4	4	20,95	15
P7 vs. P14	82,5	20	62,5	6,379	4	4	13,86	15
P7 vs. P28	82,5	7,25	75,25	6,379	4	4	16,68	15
P7 vs. Adult	82,5	7,5	75	6,379	4	4	16,63	15
P14 vs. P28	20	7,25	12,75	6,379	4	4	2,827	15
P14 vs. Adult	20	7,5	12,5	6,379	4	4	2,771	15
P28 vs. Adult	7,25	7,5	-0,25	6,379	4	4	0,05542	15

	P3	P7	P14	P28	Adult
Number of values	4	4	4	4	4
Minimum	88	75	9	5	6
25% Percentile	88,25	76,5	10,25	5	6,25
Median	100,5	82	21	6,5	7
75% Percentile	117,3	89	28,75	10,25	9,25
Maximum	119	91	29	11	10
Mean	102	82,5	20	7,25	7,5
Std. Deviation	15,85	6,608	10,03	2,872	1,732
Std. Error of Mean	7,927	3,304	5,017	1,436	0,866
Lower 95% CI	76,77	71,99	4,035	2,68	4,744
Upper 95% CI	127,2	93,01	35,97	11,82	10,26

Table S21: One-way ANOVA analysis of Profilin 1 expression.

Cofilin 1

Tukey's multiple comparisons test	Mean Diff,	95,00% CI of diff,	Significant?	Summary	Adjusted P Value	
P3 vs. P7	22	2,424 to 41,58	Yes	*	0,0242	A-B
P3 vs. P14	71	51,42 to 90,58	Yes	****	<0,0001	A-C
P3 vs. P28	81,5	61,92 to 101,1	Yes	****	<0,0001	A-D
P3 vs. Adult	82,75	63,17 to 102,3	Yes	****	<0,0001	A-E
P7 vs. P14	49	29,42 to 68,58	Yes	****	<0,0001	B-C
P7 vs. P28	59,5	39,92 to 79,08	Yes	****	<0,0001	B-D
P7 vs. Adult	60,75	41,17 to 80,33	Yes	****	<0,0001	B-E
P14 vs. P28	10,5	-9,076 to 30,08	No	ns	0,4872	C-D
P14 vs. Adult	11,75	-7,826 to 31,33	No	ns	0,3816	C-E
P28 vs. Adult	1,25	-18,33 to 20,83	No	ns	0,9996	D-E

Test details	Mean 1	Mean 2	Mean Diff,	SE of diff,	n1	n2	q	DF
P3 vs. P7	100	78	22	6,34	4	4	4,908	15
P3 vs. P14	100	29	71	6,34	4	4	15,84	15
P3 vs. P28	100	18,5	81,5	6,34	4	4	18,18	15
P3 vs. Adult	100	17,25	82,75	6,34	4	4	18,46	15
P7 vs. P14	78	29	49	6,34	4	4	10,93	15
P7 vs. P28	78	18,5	59,5	6,34	4	4	13,27	15
P7 vs. Adult	78	17,25	60,75	6,34	4	4	13,55	15
P14 vs. P28	29	18,5	10,5	6,34	4	4	2,342	15
P14 vs. Adult	29	17,25	11,75	6,34	4	4	2,621	15
P28 vs. Adult	18,5	17,25	1,25	6,34	4	4	0,2788	15

	**P3**	**P7**	**P14**	**P28**	**Adult**
Number of values	4	4	4	4	4
Minimum	96	70	20	6	8
25% Percentile	97	70,75	21,25	7	10
Median	100	78	28	16	16
75% Percentile	103	85,25	37,75	32,5	25,75
Maximum	104	86	40	36	29
Mean	100	78	29	18,5	17,25
Std. Deviation	3,266	7,703	8,602	13,5	8,694
Std. Error of Mean	1,633	3,851	4,301	6,752	4,347
Lower 95% CI	94,8	65,74	15,31	-2,986	3,416
Upper 95% CI	105,2	90,26	42,69	39,99	31,08

Table S22: One-way ANOVA analysis of Cofilin 1 expression.

α-SMA									
**Tukey's multiple comparisons test**	**Mean Diff,**	**95,00% CI of diff,**	**Significant?**	**Summary**	**Adjusted P Value**				
P3 vs. P7	18,5	-6,735 to 43,73	No	ns	0,2101	A-B			
P3 vs. P14	37,5	12,27 to 62,73	Yes	**	0,0028	A-C			
P3 vs. P28	81,25	56,02 to 106,5	Yes	****	<0,0001	A-D			
P3 vs. Adult	87,25	62,02 to 112,5	Yes	****	<0,0001	A-E			
P7 vs. P14	19	-6,235 to 44,23	No	ns	0,1906	B-C			
P7 vs. P28	62,75	37,52 to 87,98	Yes	****	<0,0001	B-D			
P7 vs. Adult	68,75	43,52 to 93,98	Yes	****	<0,0001	B-E			
P14 vs. P28	43,75	18,52 to 68,98	Yes	***	0,0007	C-D			
P14 vs. Adult	49,75	24,52 to 74,98	Yes	***	0,0002	C-E			
P28 vs. Adult	6	-19,23 to 31,23	No	ns	0,945	D-E			
Test details	Mean 1	Mean 2	Mean Diff,	SE of diff,	n1	n2	q	DF	
P3 vs. P7	100	81,5	18,5	8,172	4	4	3,201	15	
P3 vs. P14	100	62,5	37,5	8,172	4	4	6,49	15	
P3 vs. P28	100	18,75	81,25	8,172	4	4	14,06	15	
P3 vs. Adult	100	12,75	87,25	8,172	4	4	15,1	15	
P7 vs. P14	81,5	62,5	19	8,172	4	4	3,288	15	
P7 vs. P28	81,5	18,75	62,75	8,172	4	4	10,86	15	
P7 vs. Adult	81,5	12,75	68,75	8,172	4	4	11,9	15	
P14 vs. P28	62,5	18,75	43,75	8,172	4	4	7,571	15	
P14 vs. Adult	62,5	12,75	49,75	8,172	4	4	8,609	15	
P28 vs. Adult	18,75	12,75	6	8,172	4	4	1,038	15	

	**P3**	**P7**	**P14**	**P28**	**Adult**
Number of values	4	4	4	4	4
Minimum	93	68	48	5	3
25% Percentile	94,75	70	50,75	5,75	3,25
Median	100	81,5	64,5	16,5	10
75% Percentile	105,3	93	72,25	34	25
Maximum	107	95	73	37	28
Mean	100	81,5	62,5	18,75	12,75
Std. Deviation	5,715	11,9	11,39	15,02	11,76
Std. Error of Mean	2,858	5,951	5,694	7,51	5,879
Lower 95% CI	90,91	62,56	44,38	-5,149	-5,96
Upper 95% CI	109,1	100,4	80,62	42,65	31,46

Table S23: One-way ANOVA analysis of α-SMA expression.

Destrin								
**Tukey's multiple comparisons test**	**Mean Diff,**	**95,00% CI of diff,**	**Significant?**	**Summary**	**Adjusted P Value**			
P3 vs. P7	18,5	-6,735 to 43,73	No	ns	0,2101	A-B		
P3 vs. P14	37,5	12,27 to 62,73	Yes	**	0,0028	A-C		
P3 vs. P28	81,25	56,02 to 106,5	Yes	****	<0,0001	A-D		
P3 vs. Adult	87,25	62,02 to 112,5	Yes	****	<0,0001	A-E		
P7 vs. P14	19	-6,235 to 44,23	No	ns	0,1906	B-C		
P7 vs. P28	62,75	37,52 to 87,98	Yes	****	<0,0001	B-D		
P7 vs. Adult	68,75	43,52 to 93,98	Yes	****	<0,0001	B-E		
P14 vs. P28	43,75	18,52 to 68,98	Yes	***	0,0007	C-D		
P14 vs. Adult	49,75	24,52 to 74,98	Yes	***	0,0002	C-E		
P28 vs. Adult	6	-19,23 to 31,23	No	ns	0,945	D-E		
Test details	Mean 1	Mean 2	Mean Diff,	SE of diff,	n1	n2	q	DF
P3 vs. P7	100	81,5	18,5	8,172	4	4	3,201	15
P3 vs. P14	100	62,5	37,5	8,172	4	4	6,49	15
P3 vs. P28	100	18,75	81,25	8,172	4	4	14,06	15
P3 vs. Adult	100	12,75	87,25	8,172	4	4	15,1	15
P7 vs. P14	81,5	62,5	19	8,172	4	4	3,288	15
P7 vs. P28	81,5	18,75	62,75	8,172	4	4	10,86	15
P7 vs. Adult	81,5	12,75	68,75	8,172	4	4	11,9	15
P14 vs. P28	62,5	18,75	43,75	8,172	4	4	7,571	15
P14 vs. Adult	62,5	12,75	49,75	8,172	4	4	8,609	15
P28 vs. Adult	18,75	12,75	6	8,172	4	4	1,038	15

	**P3**	**P7**	**P14**	**P28**	**Adult**
Number of values	4	4	4	4	4
Minimum	93	68	48	5	3
25% Percentile	94,75	70	50,75	5,75	3,25
Median	100	81,5	64,5	16,5	10
75% Percentile	105,3	93	72,25	34	25
Maximum	107	95	73	37	28
Mean	100	81,5	62,5	18,75	12,75
Std. Deviation	5,715	11,9	11,39	15,02	11,76
Std. Error of Mean	2,858	5,951	5,694	7,51	5,879
Lower 95% CI	90,91	62,56	44,38	-5,149	-5,96
Upper 95% CI	109,1	100,4	80,62	42,65	31,46

Table S24: One-way ANOVA analysis of Destrin expression.

Ezrin

Tukey's multiple comparisons test	Mean Diff,	95,00% CI of diff,	Significant?	Summary	Adjusted P Value	
P3 vs. P7	16,75	7,358 to 26,14	Yes	***	0,0005	A-B
P3 vs. P14	68	58,61 to 77,39	Yes	****	<0,0001	A-C
P3 vs. P28	84,75	75,36 to 94,14	Yes	****	<0,0001	A-D
P3 vs. Adult	92	82,61 to 101,4	Yes	****	<0,0001	A-E
P7 vs. P14	51,25	41,86 to 60,64	Yes	****	<0,0001	B-C
P7 vs. P28	68	58,61 to 77,39	Yes	****	<0,0001	B-D
P7 vs. Adult	75,25	65,86 to 84,64	Yes	****	<0,0001	B-E
P14 vs. P28	16,75	7,358 to 26,14	Yes	***	0,0005	C-D
P14 vs. Adult	24	14,61 to 33,39	Yes	****	<0,0001	C-E
P28 vs. Adult	7,25	-2,142 to 16,64	No	ns	0,1733	D-E

Test details	Mean 1	Mean 2	Mean Diff,	SE of diff,	n1	n2	q	DF
P3 vs. P7	100	83,25	16,75	3,041	4	4	7,789	15
P3 vs. P14	100	32	68	3,041	4	4	31,62	15
P3 vs. P28	100	15,25	84,75	3,041	4	4	39,41	15
P3 vs. Adult	100	8	92	3,041	4	4	42,78	15
P7 vs. P14	83,25	32	51,25	3,041	4	4	23,83	15
P7 vs. P28	83,25	15,25	68	3,041	4	4	31,62	15
P7 vs. Adult	83,25	8	75,25	3,041	4	4	34,99	15
P14 vs. P28	32	15,25	16,75	3,041	4	4	7,789	15
P14 vs. Adult	32	8	24	3,041	4	4	11,16	15
P28 vs. Adult	15,25	8	7,25	3,041	4	4	3,371	15

	P3	P7	P14	P28	Adult
Number of values	4	4	4	4	4
Minimum	96	78	26	14	6
25% Percentile	96	78,75	26,75	14	6
Median	98	84	32	15	7
75% Percentile	106	87	37,25	16,75	11
Maximum	108	87	38	17	12
Mean	100	83,25	32	15,25	8
Std. Deviation	5,657	4,5	5,477	1,5	2,828
Std. Error of Mean	2,828	2,25	2,739	0,75	1,414
Lower 95% CI	91	76,09	23,28	12,86	3,499
Upper 95% CI	109	90,41	40,72	17,64	12,5

Table S25: One-way ANOVA analysis of Ezrin expression.

Radixin

Tukey's multiple comparisons test	Mean Diff,	95,00% CI of diff	Significant?	Summary	Adjusted P Value			
P3 vs. P7	-5,5	-41,62 to 30,62	No	ns	0,989	A-B		
P3 vs. P14	12,5	-23,62 to 48,62	No	ns	0,8195	A-C		
P3 vs. P28	35,75	-0,3734 to 71,87	No	ns	0,0531	A-D		
P3 vs. Adult	40,75	4,627 to 76,87	Yes	*	0,0236	A-E		
P7 vs. P14	18	-18,12 to 54,12	No	ns	0,555	B-C		
P7 vs. P28	41,25	5,127 to 77,37	Yes	*	0,0218	B-D		
P7 vs. Adult	46,25	10,13 to 82,37	Yes	**	0,0095	B-E		
P14 vs. P28	23,25	-12,87 to 59,37	No	ns	0,3178	C-D		
P14 vs. Adult	28,25	-7,873 to 64,37	No	ns	0,1646	C-E		
P28 vs. Adult	5	-31,12 to 41,12	No	ns	0,9923	D-E		
Test details	Mean 1	Mean 2	Mean Diff,	SE of diff,	n1	n2	q	DF
P3 vs. P7	100	105,5	-5,5	11,7	4	4	0,6649	15
P3 vs. P14	100	87,5	12,5	11,7	4	4	1,511	15
P3 vs. P28	100	64,25	35,75	11,7	4	4	4,322	15
P3 vs. Adult	100	59,25	40,75	11,7	4	4	4,926	15
P7 vs. P14	105,5	87,5	18	11,7	4	4	2,176	15
P7 vs. P28	105,5	64,25	41,25	11,7	4	4	4,987	15
P7 vs. Adult	105,5	59,25	46,25	11,7	4	4	5,591	15
P14 vs. P28	87,5	64,25	23,25	11,7	4	4	2,811	15
P14 vs. Adult	87,5	59,25	28,25	11,7	4	4	3,415	15
P28 vs. Adult	64,25	59,25	5	11,7	4	4	0,6045	15

	P3	P7	P14	P28	Adult
Number of values	4	4	4	4	4
Minimum	87	79	63	62	49
25% Percentile	88,25	82	66	62,25	51,25
Median	100	103	89,5	63,5	61
75% Percentile	111,8	131,5	107	67	65,5
Maximum	113	137	108	68	66
Mean	100	105,5	87,5	64,25	59,25
Std. Deviation	12,46	25,79	21,98	2,63	7,632
Std. Error of Mean	6,232	12,89	10,99	1,315	3,816
Lower 95% CI	80,17	64,47	52,53	60,07	47,11
Upper 95% CI	119,8	146,5	122,5	68,43	71,39

Table S26: One-way ANOVA analysis of Radixin expression.

Moesin

Tukey's multiple comparisons test	Mean Diff,	95,00% CI of diff,	Significant?	Summary	Adjusted P Value	
P3 vs. P7	-6,5	-25,87 to 12,87	No	ns	0,8348	A-B
P3 vs. P14	61	41,63 to 80,37	Yes	****	<0,0001	A-C
P3 vs. P28	77,75	58,38 to 97,12	Yes	****	<0,0001	A-D
P3 vs. Adult	87,25	67,88 to 106,6	Yes	****	<0,0001	A-E
P7 vs. P14	67,5	48,13 to 86,87	Yes	****	<0,0001	B-C
P7 vs. P28	84,25	64,88 to 103,6	Yes	****	<0,0001	B-D
P7 vs. Adult	93,75	74,38 to 113,1	Yes	****	<0,0001	B-E
P14 vs. P28	16,75	-2,620 to 36,12	No	ns	0,1066	C-D
P14 vs. Adult	26,25	6,880 to 45,62	Yes	**	0,0061	C-E
P28 vs. Adult	9,5	-9,870 to 28,87	No	ns	0,5693	D-E

Test details	Mean 1	Mean 2	Mean Diff,	SE of diff,	n1	n2	q	DF
P3 vs. P7	100	106,5	-6,5	6,273	4	4	1,465	15
P3 vs. P14	100	39	61	6,273	4	4	13,75	15
P3 vs. P28	100	22,25	77,75	6,273	4	4	17,53	15
P3 vs. Adult	100	12,75	87,25	6,273	4	4	19,67	15
P7 vs. P14	106,5	39	67,5	6,273	4	4	15,22	15
P7 vs. P28	106,5	22,25	84,25	6,273	4	4	18,99	15
P7 vs. Adult	106,5	12,75	93,75	6,273	4	4	21,14	15
P14 vs. P28	39	22,25	16,75	6,273	4	4	3,776	15
P14 vs. Adult	39	12,75	26,25	6,273	4	4	5,918	15
P28 vs. Adult	22,25	12,75	9,5	6,273	4	4	2,142	15

	P3	P7	P14	P28	Adult
Number of values	4	4	4	4	4
Minimum	87	86	32	17	9
25% Percentile	89,75	91,25	33,75	17,75	9,25
Median	100	110,5	40	22,5	11,5
75% Percentile	110,3	117,8	43,25	26,5	17,5
Maximum	113	119	44	27	19
Mean	100	106,5	39	22,25	12,75
Std. Deviation	10,74	14,53	5,099	4,573	4,5
Std. Error of Mean	5,37	7,263	2,55	2,287	2,25
Lower 95% CI	82,91	83,39	30,89	14,97	5,589
Upper 95% CI	117,1	129,6	47,11	29,53	19,91

Table S27: One-way ANOVA analysis of Moesin expression.

P-ERM

Tukey's multiple comparisons tes	Mean Diff,	95,00% CI of diff,	Significant?	Summary	Adjusted P Value	
P3 vs. P7	17,75	-1,170 to 36,67	No	ns	0,0711	A-B
P3 vs. P14	81	62,08 to 99,92	Yes	****	<0,0001	A-C
P3 vs. P28	90,25	71,33 to 109,2	Yes	****	<0,0001	A-D
P3 vs. Adult	95,75	76,83 to 114,7	Yes	****	<0,0001	A-E
P7 vs. P14	63,25	44,33 to 82,17	Yes	****	<0,0001	B-C
P7 vs. P28	72,5	53,58 to 91,42	Yes	****	<0,0001	B-D
P7 vs. Adult	78	59,08 to 96,92	Yes	****	<0,0001	B-E
P14 vs. P28	9,25	-9,670 to 28,17	No	ns	0,5721	C-D
P14 vs. Adult	14,75	-4,170 to 33,67	No	ns	0,1667	C-E
P28 vs. Adult	5,5	-13,42 to 24,42	No	ns	0,8934	D-E

Test details	Mean 1	Mean 2	Mean Diff,	SE of diff,	n1	n2	q	DF
P3 vs. P7	100	82,25	17,75	6,127	4	4	4,097	15
P3 vs. P14	100	19	81	6,127	4	4	18,7	15
P3 vs. P28	100	9,75	90,25	6,127	4	4	20,83	15
P3 vs. Adult	100	4,25	95,75	6,127	4	4	22,1	15
P7 vs. P14	82,25	19	63,25	6,127	4	4	14,6	15
P7 vs. P28	82,25	9,75	72,5	6,127	4	4	16,73	15
P7 vs. Adult	82,25	4,25	78	6,127	4	4	18	15
P14 vs. P28	19	9,75	9,25	6,127	4	4	2,135	15
P14 vs. Adult	19	4,25	14,75	6,127	4	4	3,404	15
P28 vs. Adult	9,75	4,25	5,5	6,127	4	4	1,269	15

	P3	P7	P14	P28	Adult
Number of values	4	4	4	4	4
Minimum	86	71	6	5	3
25% Percentile	89,25	72,25	8,5	5,5	3
Median	100	82	20,5	9	3,5
75% Percentile	110,8	92,5	28	14,75	6,25
Maximum	114	94	29	16	7
Mean	100	82,25	19	9,75	4,25
Std. Deviation	11,46	10,59	10,23	4,856	1,893
Std. Error of Mean	5,73	5,297	5,115	2,428	0,9465
Lower 95% CI	81,76	65,39	2,721	2,023	1,238
Upper 95% CI	118,2	99,11	35,28	17,48	7,262

Table S28: One-way ANOVA analysis of P-ERM expression.

Merlin								
**Tukey's multiple comparisons test**	**Mean Diff,**	**95,00% CI of diff,**	**Significant?**	**Summary**	**Adjusted P Value**			
P3 vs. P7	-2,25	-18,66 to 14,16	No	ns	0,9926	A-B		
P3 vs. P14	42,25	25,84 to 58,66	Yes	****	<0,0001	A-C		
P3 vs. P28	72,75	56,34 to 89,16	Yes	****	<0,0001	A-D		
P3 vs. Adult	81,5	65,09 to 97,91	Yes	****	<0,0001	A-E		
P7 vs. P14	44,5	28,09 to 60,91	Yes	****	<0,0001	B-C		
P7 vs. P28	75	58,59 to 91,41	Yes	****	<0,0001	B-D		
P7 vs. Adult	83,75	67,34 to 100,2	Yes	****	<0,0001	B-E		
P14 vs. P28	30,5	14,09 to 46,91	Yes	***	0,0003	C-D		
P14 vs. Adult	39,25	22,84 to 55,66	Yes	****	<0,0001	C-E		
P28 vs. Adult	8,75	-7,660 to 25,16	No	ns	0,4927	D-E		
Test details	Mean 1	Mean 2	Mean Diff,	SE of diff,	n1	n2	q	DF
P3 vs. P7	100	102,3	-2,25	5,314	4	4	0,5988	15
P3 vs. P14	100	57,75	42,25	5,314	4	4	11,24	15
P3 vs. P28	100	27,25	72,75	5,314	4	4	19,36	15
P3 vs. Adult	100	18,5	81,5	5,314	4	4	21,69	15
P7 vs. P14	102,3	57,75	44,5	5,314	4	4	11,84	15
P7 vs. P28	102,3	27,25	75	5,314	4	4	19,96	15
P7 vs. Adult	102,3	18,5	83,75	5,314	4	4	22,29	15
P14 vs. P28	57,75	27,25	30,5	5,314	4	4	8,117	15
P14 vs. Adult	57,75	18,5	39,25	5,314	4	4	10,45	15
P28 vs. Adult	27,25	18,5	8,75	5,314	4	4	2,329	15

	**P3**	**P7**	**P14**	**P28**	**Adult**
Number of values	4	4	4	4	4
Minimum	94	84	50	24	16
25% Percentile	95,5	88,5	50,75	24,5	16,5
Median	100	106	55,5	27,5	19
75% Percentile	104,5	112,3	67	29,75	20
Maximum	106	113	70	30	20
Mean	100	102,3	57,75	27,25	18,5
Std. Deviation	4,899	13,02	8,808	2,754	1,915
Std. Error of Mean	2,449	6,511	4,404	1,377	0,9574
Lower 95% CI	92,2	81,53	43,73	22,87	15,45
Upper 95% CI	107,8	123	71,77	31,63	21,55

Table S29: One-way ANOVA analysis of Merlin expression.

Cytochrome c

Tukey's multiple comparisons test	Mean Diff,	95,00% CI of diff,	Significant?	Summary	Adjusted P Value			
P3 vs. P7	-116	-250,9 to 18,93	No	ns	0,1095	A-B		
P3 vs. P14	-119,8	-254,7 to 15,18	No	ns	0,0942	A-C		
P3 vs. P28	-135,3	-270,2 to -0,3177	Yes	*	0,0493	A-D		
P3 vs. Adult	-139,3	-274,2 to -4,318	Yes	*	0,0416	A-E		
P7 vs. P14	-3,75	-138,7 to 131,2	No	ns	>0,9999	B-C		
P7 vs. P28	-19,25	-154,2 to 115,7	No	ns	0,9914	B-D		
P7 vs. Adult	-23,25	-158,2 to 111,7	No	ns	0,9825	B-E		
P14 vs. P28	-15,5	-150,4 to 119,4	No	ns	0,9962	C-D		
P14 vs. Adult	-19,5	-154,4 to 115,4	No	ns	0,9909	C-E		
P28 vs. Adult	-4	-138,9 to 130,9	No	ns	>0,9999	D-E		
Test details	Mean 1	Mean 2	Mean Diff,	SE of diff,	n1	n2	q	DF
P3 vs. P7	100	216	-116	43,7	4	4	3,754	15
P3 vs. P14	100	219,8	-119,8	43,7	4	4	3,876	15
P3 vs. P28	100	235,3	-135,3	43,7	4	4	4,377	15
P3 vs. Adult	100	239,3	-139,3	43,7	4	4	4,507	15
P7 vs. P14	216	219,8	-3,75	43,7	4	4	0,1214	15
P7 vs. P28	216	235,3	-19,25	43,7	4	4	0,623	15
P7 vs. Adult	216	239,3	-23,25	43,7	4	4	0,7525	15
P14 vs. P28	219,8	235,3	-15,5	43,7	4	4	0,5016	15
P14 vs. Adult	219,8	239,3	-19,5	43,7	4	4	0,6311	15
P28 vs. Adult	235,3	239,3	-4	43,7	4	4	0,1295	15

	P3	P7	P14	P28	Adult
Number of values	4	4	4	4	4
Minimum	75	187	142	185	175
25% Percentile	77	189,3	164	188,5	178,8
Median	100	200	239	217,5	196
75% Percentile	123	258,8	256,3	299,8	343
Maximum	125	277	259	321	390
Mean	100	216	219,8	235,3	239,3
Std. Deviation	24,68	41,26	53,19	61,08	101,1
Std. Error of Mean	12,34	20,63	26,6	30,54	50,55
Lower 95% CI	60,72	150,4	135,1	138,1	78,37
Upper 95% CI	139,3	281,6	304,4	332,4	400,1

Table S30: One-way ANOVA analysis of Cytochrome c expression.

Mb								
**Tukey's multiple comparisons test**	**Mean Diff,**	**95,00% CI of diff,**	**Significant?**	**Summary**	**Adjusted P Value**			
P3 vs. P7	-74,75	-249,0 to 99,49	No	ns	0,6809	A-B		
P3 vs. P14	-92	-266,2 to 82,24	No	ns	0,5018	A-C		
P3 vs. P28	-153,5	-327,7 to 20,74	No	ns	0,0976	A-D		
P3 vs. Adult	-254,3	-428,5 to -80,01	Yes	**	0,0033	A-E		
P7 vs. P14	-17,25	-191,5 to 157,0	No	ns	0,9979	B-C		
P7 vs. P28	-78,75	-253,0 to 95,49	No	ns	0,6394	B-D		
P7 vs. Adult	-179,5	-353,7 to -5,265	Yes	*	0,042	B-E		
P14 vs. P28	-61,5	-235,7 to 112,7	No	ns	0,8089	C-D		
P14 vs. Adult	-162,3	-336,5 to 11,99	No	ns	0,0739	C-E		
P28 vs. Adult	-100,8	-275,0 to 73,49	No	ns	0,4165	D-E		
Test details	Mean 1	Mean 2	Mean Diff,	SE of diff,	n1	n2	q	DF
P3 vs. P7	100	174,8	-74,75	56,42	4	4	1,874	15
P3 vs. P14	100	192	-92	56,42	4	4	2,306	15
P3 vs. P28	100	253,5	-153,5	56,42	4	4	3,847	15
P3 vs. Adult	100	354,3	-254,3	56,42	4	4	6,372	15
P7 vs. P14	174,8	192	-17,25	56,42	4	4	0,4323	15
P7 vs. P28	174,8	253,5	-78,75	56,42	4	4	1,974	15
P7 vs. Adult	174,8	354,3	-179,5	56,42	4	4	4,499	15
P14 vs. P28	192	253,5	-61,5	56,42	4	4	1,541	15
P14 vs. Adult	192	354,3	-162,3	56,42	4	4	4,067	15
P28 vs. Adult	253,5	354,3	-100,8	56,42	4	4	2,525	15

	**P3**	**P7**	**P14**	**P28**	**Adult**
Number of values	4	4	4	4	4
Minimum	97	156	140	163	208
25% Percentile	97,25	156,3	142	167,8	224
Median	100	158,5	182,5	248	366
75% Percentile	102,8	209,5	251,5	344,8	472,8
Maximum	103	226	263	355	477
Mean	100	174,8	192	253,5	354,3
Std. Deviation	2,944	34,21	58,61	95,33	134,7
Std. Error of Mean	1,472	17,1	29,31	47,67	67,33
Lower 95% CI	95,32	120,3	98,74	101,8	140
Upper 95% CI	104,7	229,2	285,3	405,2	568,5

Table S31: One-way ANOVA analysis of Mb expression.

P-H3

Tukey's multiple comparisons tes	Mean Diff,	95,00% CI of diff,	Significant?	Summary	Adjusted P Value	
P3 vs. P7	-31,75	-61,61 to -1,889	Yes	*	0,0346	A-B
P3 vs. P14	79,75	49,89 to 109,6	Yes	****	<0,0001	A-C
P3 vs. P28	100	70,14 to 129,9	Yes	****	<0,0001	A-D
P3 vs. Adult	100	70,14 to 129,9	Yes	****	<0,0001	A-E
P7 vs. P14	111,5	81,64 to 141,4	Yes	****	<0,0001	B-C
P7 vs. P28	131,8	101,9 to 161,6	Yes	****	<0,0001	B-D
P7 vs. Adult	131,8	101,9 to 161,6	Yes	****	<0,0001	B-E
P14 vs. P28	20,25	-9,611 to 50,11	No	ns	0,2724	C-D
P14 vs. Adult	20,25	-9,611 to 50,11	No	ns	0,2724	C-E
P28 vs. Adult	0	-29,86 to 29,86	No	ns	>0,9999	D-E

Test details	Mean 1	Mean 2	Mean Diff,	SE of diff,	n1	n2	q	DF
P3 vs. P7	100	131,8	-31,75	9,67	4	4	4,643	15
P3 vs. P14	100	20,25	79,75	9,67	4	4	11,66	15
P3 vs. P28	100	0	100	9,67	4	4	14,62	15
P3 vs. Adult	100	0	100	9,67	4	4	14,62	15
P7 vs. P14	131,8	20,25	111,5	9,67	4	4	16,31	15
P7 vs. P28	131,8	0	131,8	9,67	4	4	19,27	15
P7 vs. Adult	131,8	0	131,8	9,67	4	4	19,27	15
P14 vs. P28	20,25	0	20,25	9,67	4	4	2,961	15
P14 vs. Adult	20,25	0	20,25	9,67	4	4	2,961	15
P28 vs. Adult	0	0	0	9,67	4	4	0	15

	P3	P7	P14	P28	Adult
Number of values	4	4	4	4	4
Minimum	91	105	13	0	0
25% Percentile	93,25	108,5	13,75	0	0
Median	100	125	20	0	0
75% Percentile	106,8	161,8	27	0	0
Maximum	109	172	28	0	0
Mean	100	131,8	20,25	0	0
Std. Deviation	7,348	28,86	6,946	0	0
Std. Error of Mean	3,674	14,43	3,473	0	0
Lower 95% CI	88,31	85,83	9,197	0	0
Upper 95% CI	111,7	177,7	31,3	0	0

Table S32: One-way ANOVA analysis of P-H3 expression.

Aurora B

Tukey's multiple comparisons test	Mean Diff,	95,00% CI of diff,	Significant?	Summary	Adjusted P Value	
P3 vs. P7	39,75	9,095 to 70,40	Yes	**	0,0086	A-B
P3 vs. P14	97	66,35 to 127,7	Yes	****	<0,0001	A-C
P3 vs. P28	98,75	68,10 to 129,4	Yes	****	<0,0001	A-D
P3 vs. Adult	99,5	68,85 to 130,2	Yes	****	<0,0001	A-E
P7 vs. P14	57,25	26,60 to 87,90	Yes	***	0,0003	B-C
P7 vs. P28	59	28,35 to 89,65	Yes	***	0,0002	B-D
P7 vs. Adult	59,75	29,10 to 90,40	Yes	***	0,0002	B-E
P14 vs. P28	1,75	-28,90 to 32,40	No	ns	0,9998	C-D
P14 vs. Adult	2,5	-28,15 to 33,15	No	ns	0,999	C-E
P28 vs. Adult	0,75	-29,90 to 31,40	No	ns	>0,9999	D-E

Test details	Mean 1	Mean 2	Mean Diff,	SE of diff,	n1	n2	q	DF
P3 vs. P7	100	60,25	39,75	9,927	4	4	5,663	15
P3 vs. P14	100	3	97	9,927	4	4	13,82	15
P3 vs. P28	100	1,25	98,75	9,927	4	4	14,07	15
P3 vs. Adult	100	0,5	99,5	9,927	4	4	14,17	15
P7 vs. P14	60,25	3	57,25	9,927	4	4	8,156	15
P7 vs. P28	60,25	1,25	59	9,927	4	4	8,405	15
P7 vs. Adult	60,25	0,5	59,75	9,927	4	4	8,512	15
P14 vs. P28	3	1,25	1,75	9,927	4	4	0,2493	15
P14 vs. Adult	3	0,5	2,5	9,927	4	4	0,3561	15
P28 vs. Adult	1,25	0,5	0,75	9,927	4	4	0,1068	15

	P3	P7	P14	P28	Adult
Number of values	4	4	4	4	4
Minimum	97	32	1	0	0
25% Percentile	97,5	33	1	0	0
Median	100	57,5	1	0,5	0,5
75% Percentile	102,5	90,25	7	3,25	1
Maximum	103	94	9	4	1
Mean	100	60,25	3	1,25	0,5
Std. Deviation	2,582	30,97	4	1,893	0,5774
Std. Error of Mean	1,291	15,48	2	0,9465	0,2887
Lower 95% CI	95,89	10,98	-3,365	-1,762	-0,4187
Upper 95% CI	104,1	109,5	9,365	4,262	1,419

S33: One-way ANOVA analysis of Aurora B expression.

Figure S1: Uncropped Western blot corresponding to Figure 2B and 2G

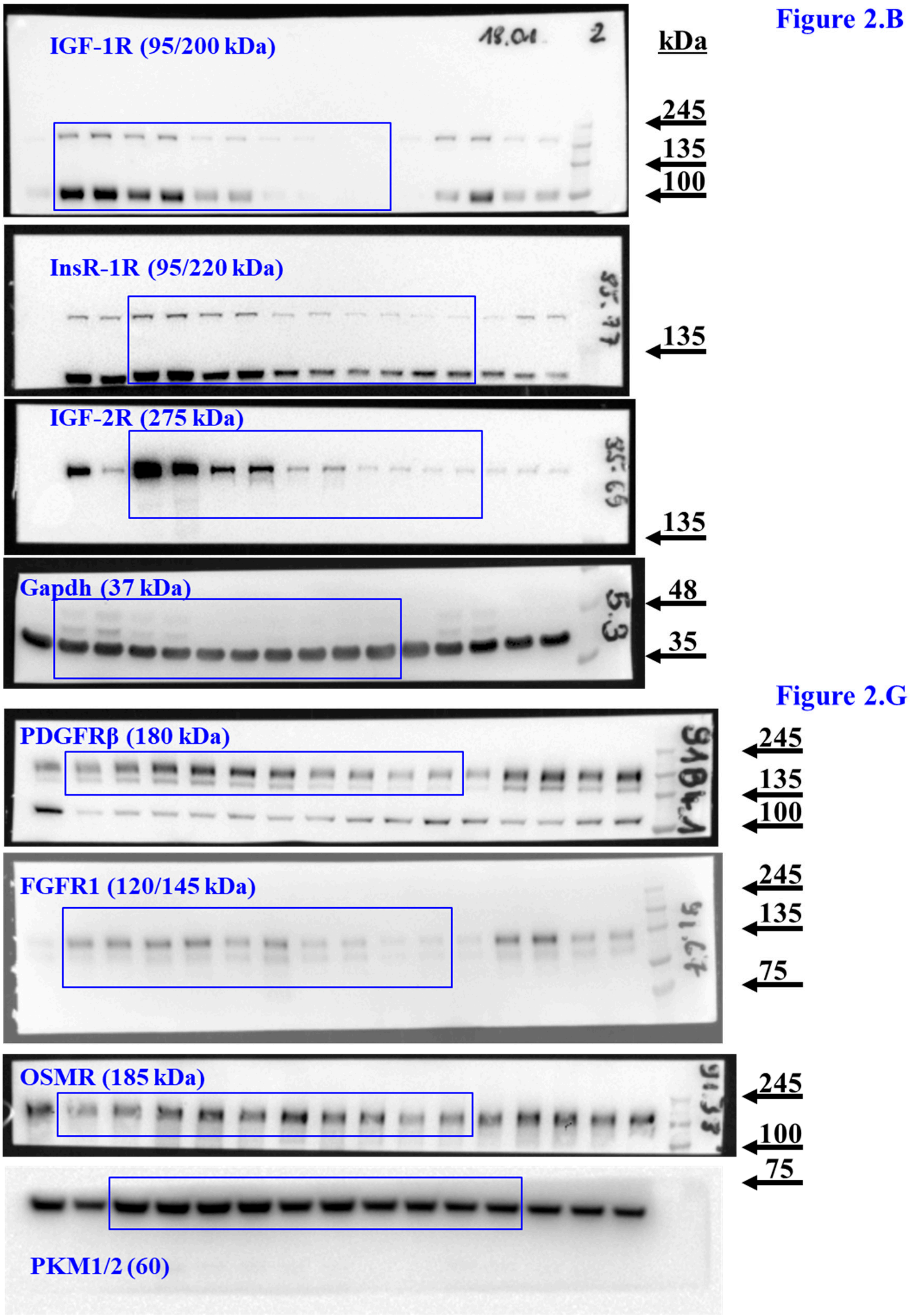

Figure S2: Uncropped Western blot corresponding to Figure 3A.

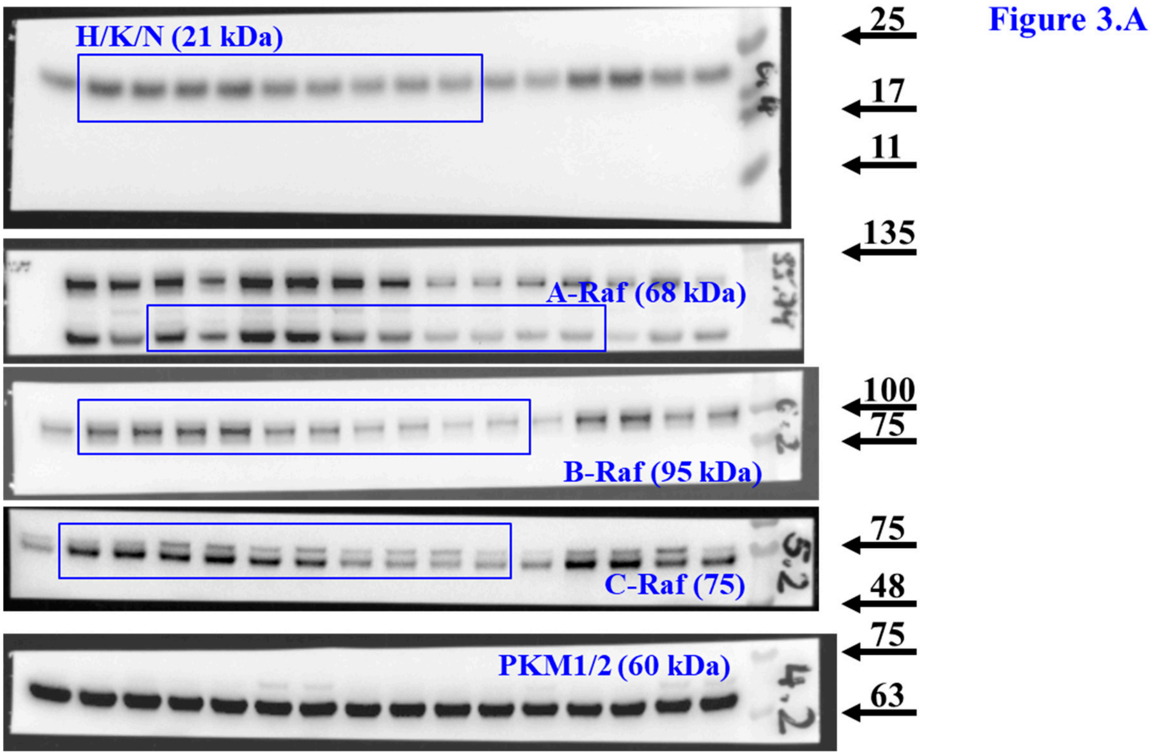

Figure S3: Uncropped Western blot corresponding to Figure 3F.

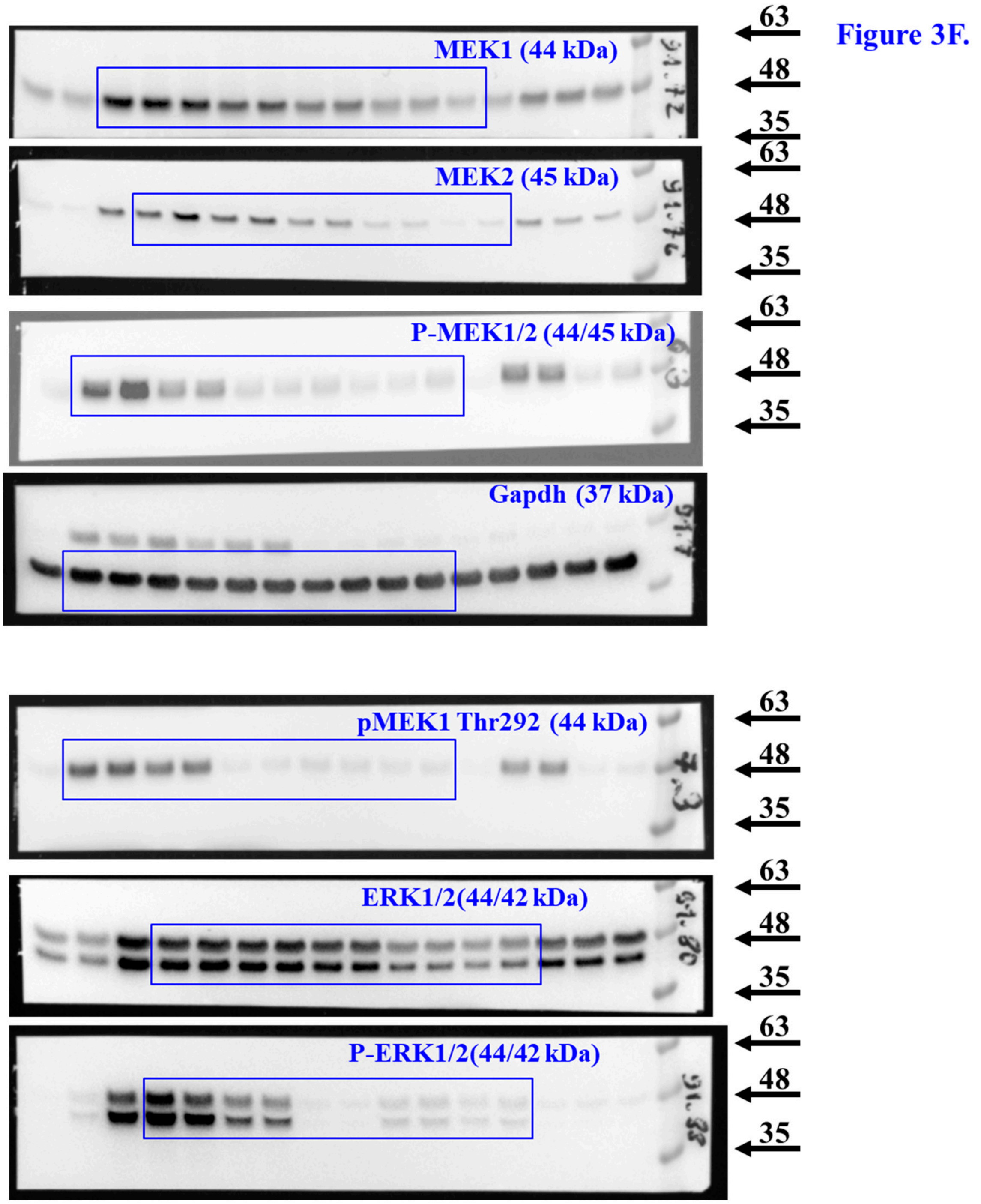

Figure S4: Uncropped Western blot corresponding to Figure 4.

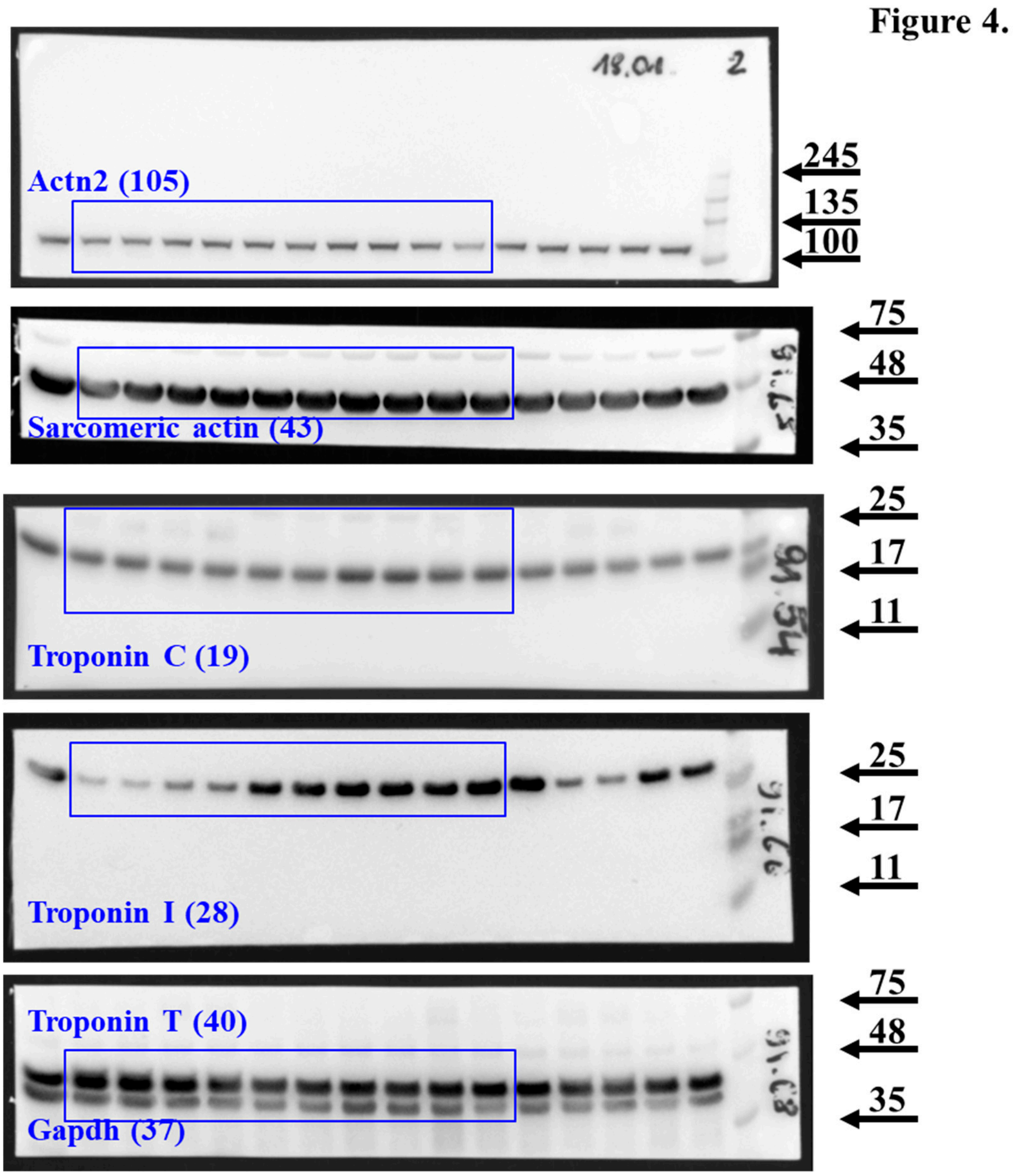

Figure S5: Uncropped Western blot corresponding to Figure 5.

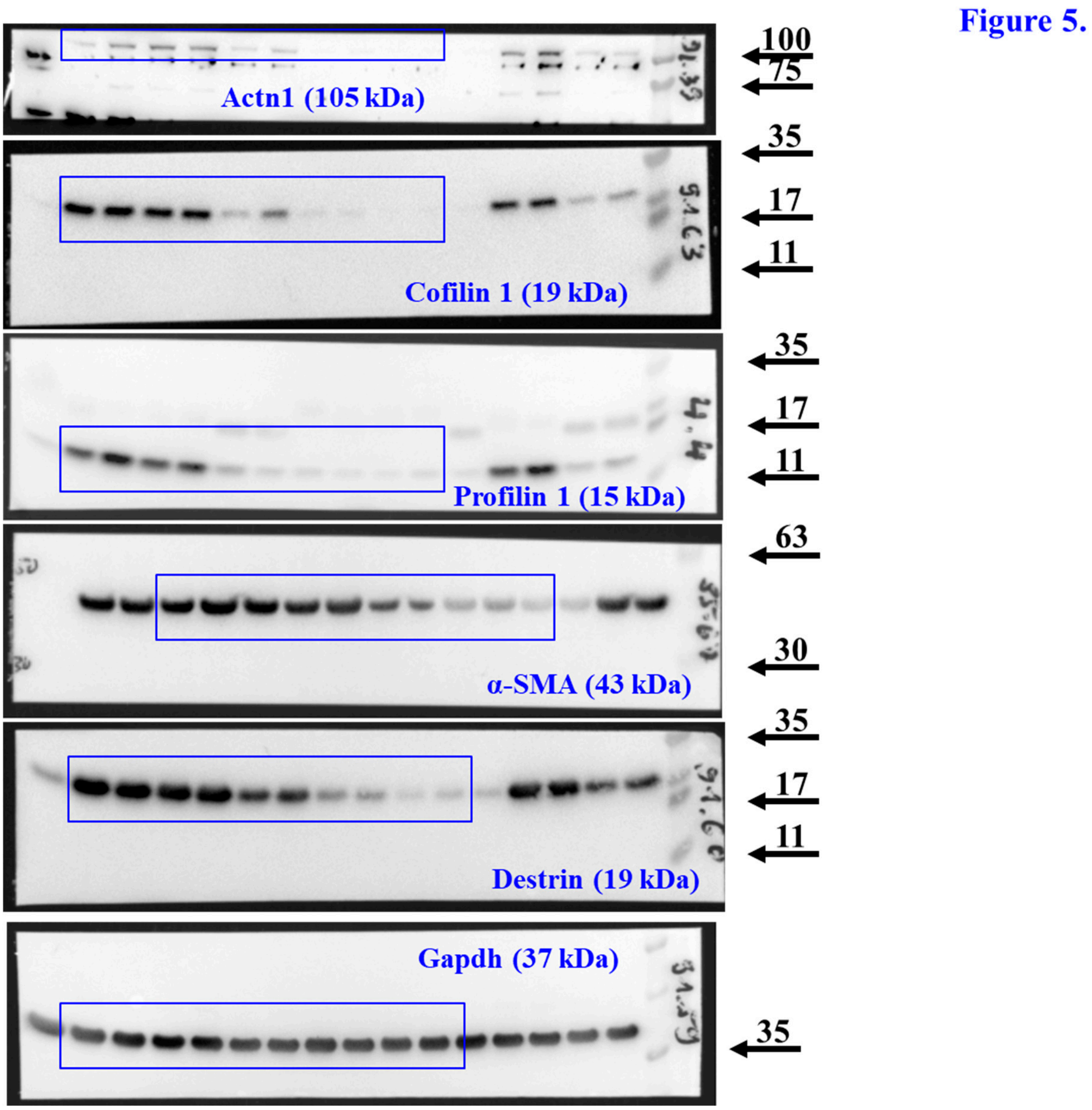

Figure S6: Uncropped Western blot corresponding to Figure 6.

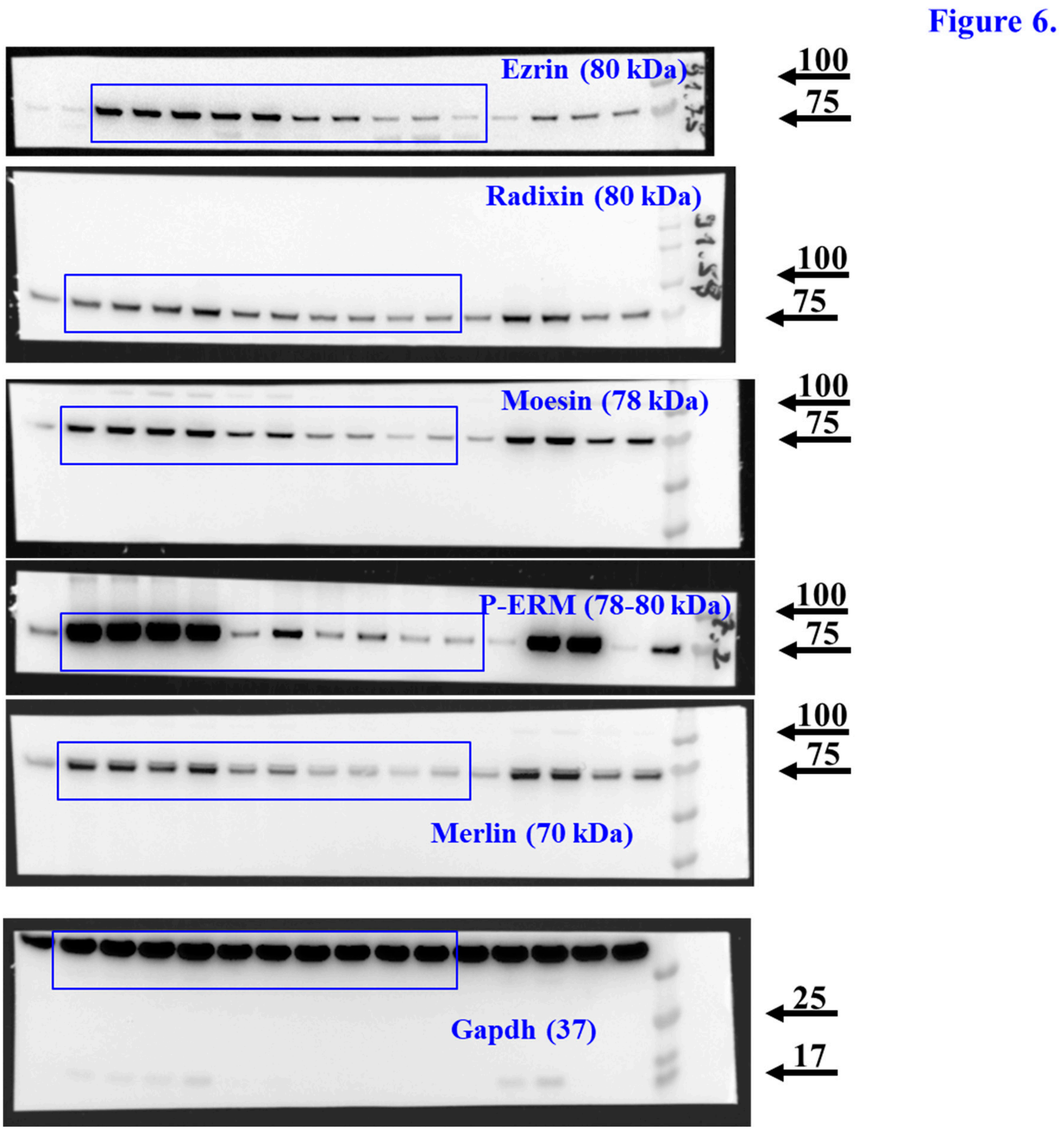

Figure S7: Uncropped Western blot corresponding to Figure 7.

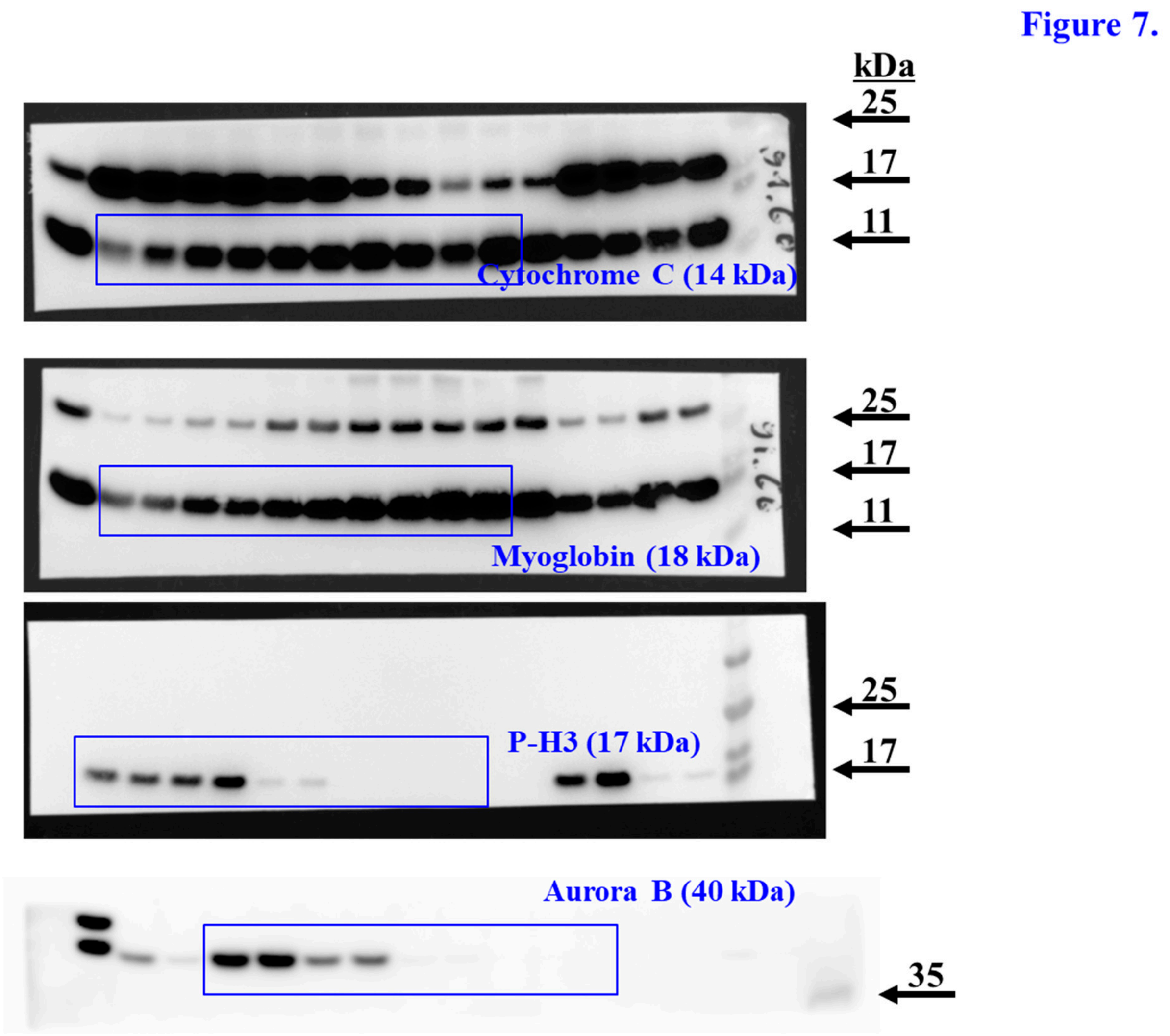

Supplement: Supplementary file 1 [file cells-15-00873-s001.zip › cells-4242667-supplementary.pdf]
